# Speeding Up Ecological and Evolutionary Computations in R; Essentials of High Performance Computing for Biologists

**DOI:** 10.1371/journal.pcbi.1004140

**Published:** 2015-03-26

**Authors:** Marco D. Visser, Sean M. McMahon, Cory Merow, Philip M. Dixon, Sydne Record, Eelke Jongejans

**Affiliations:** 1 Departments of Experimental Plant Ecology and Animal Ecology & Ecophysiology, Radboud University Nijmegen, Nijmegen, The Netherlands; 2 Program for Applied Ecology, Centre for Tropical Forest Science, Smithsonian Tropical Research Institute, Balboa, Ancón, Panamá, Republic of Panamá; 3 Smithsonian Environmental Research Center, Edgewater, Maryland, United States of America; 4 Department of Ecology and Evolutionary Biology, University of Connecticut, Storrs, Connecticut, United States of America; 5 Department of Statistics, Iowa State University, Ames, Iowa, United States of America; 6 Harvard University, Harvard Forest, Petersham, Massachusetts, United States of America; 7 Bryn Mawr College, Bryn Mawr, Pennsylvania, United States of America; Ontario Institute for Cancer Research, CANADA

## Abstract

Computation has become a critical component of research in biology. A risk has emerged that computational and programming challenges may limit research scope, depth, and quality. We review various solutions to common computational efficiency problems in ecological and evolutionary research. Our review pulls together material that is currently scattered across many sources and emphasizes those techniques that are especially effective for typical ecological and environmental problems. We demonstrate how straightforward it can be to write efficient code and implement techniques such as profiling or parallel computing. We supply a newly developed R package (*aprof*) that helps to identify computational bottlenecks in R code and determine whether optimization can be effective. Our review is complemented by a practical set of examples and detailed Supporting Information material (S1–S3 Texts) that demonstrate large improvements in computational speed (ranging from 10.5 times to 14,000 times faster). By improving computational efficiency, biologists can feasibly solve more complex tasks, ask more ambitious questions, and include more sophisticated analyses in their research.

*This is part of the PLOS Computational Biology Education collection.*

## Introduction

Emerging fields such as ecoinformatics and computational ecology [1,2] bear witness to the fact that biology is becoming more quantitative and interdisciplinary. Such research often requires intensive computing, which may be limited by inefficient code that confines the size of a simulation model or restricts the scope of data analysis. It is therefore increasingly necessary for biologists to become versed in efficient programming [3], as well as in mathematics and statistics [4].

Computer scientists have developed many optimization methods (e.g., [5]), however, the efficient translation of mathematical models to computer code has received very little attention in biology [2]. Here we present an overview of techniques to improve computational efficiency in a wide variety of settings. Much of the information we present is currently scattered throughout various textbooks, articles, or online sources, and our goal here is to provide a convenient summary for biologists interested in improving the efficiency of their computational methods. In short, we 1) highlight the processes that slow down computation; 2) introduce techniques, which, via an R package, help to decide whether and where optimization is needed; 3) give a step-by-step guide to implementing various basic, but powerful techniques for optimization; and 4) demonstrate the speed gains that can be achieved. We supplement this with more background information and detailed examples in the Supporting Information (S1–S3 Texts). The widespread adoption in the biological sciences of the R programming language has motivated a focus on techniques that are directly applicable to R—although many principles hold for other platforms.

## Focus

**Fig 1:**
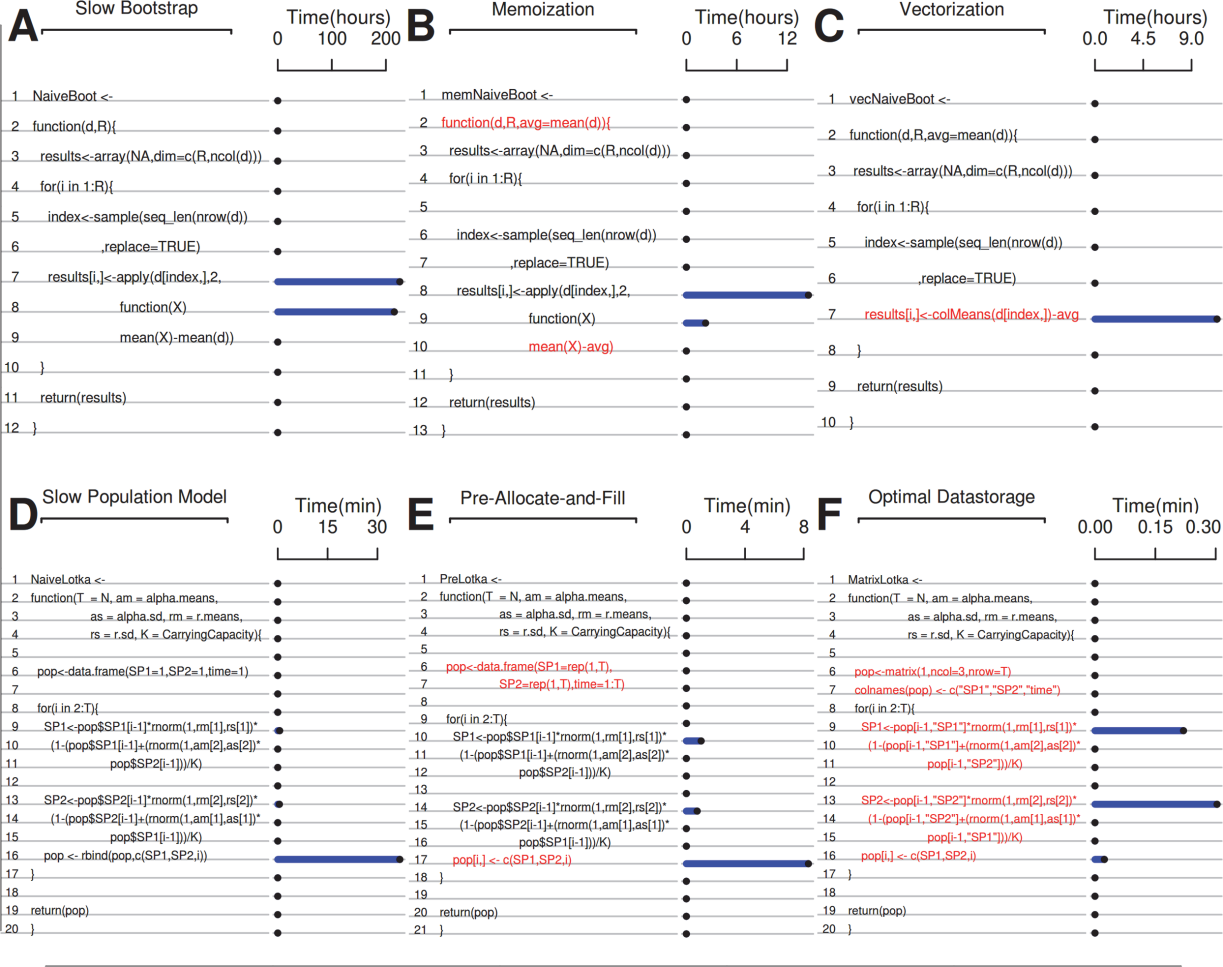
Visualization of profiling output using the *aprof* package for R code, where the amount of time spent in each line of code is indicated by the blue bars. In A, B and C, a bootstrap algorithm is shown, and in D, E, and F, a stochastic Lotka-Volterra competition model is shown. The consecutive optimizations described in the text are indicated with the red lines in B, C, E, and F indicating the altered pieces of code. (A) An inefficiently coded bootstrap algorithm, with most time spent in lines 7–8. This algorithm shuffles the values of a large matrix (750,000 x 1000) stored in object "d", and then calculates columnwise the difference between the mean column values and the overall mean. (B) A slightly improved code where the overall mean calculation is stored in object "avg." (C) A further improved version of the code where column means are calculated by a specialized and vectorized function (*colMeans*). (D) A slow running stochastic Lotka-Volterra model of species coexistence that runs a simulation over T years where species have normally distributed intrinsic growth rates (r ∼ Norm(rm,rs)) and competition coefficients (a ∼ Norm(am,as)). (E) the Lotka-Volterra model is more efficient when the pre-allocation-and-fill method is applied. (F) Switching to a matrix to store results further decreases run time. A detailed description of each optimization step with profiling analysis is given online (S1 Text, sections 2 and 6).

Many analyses in biology are computationally demanding. Examples include large matrix operations [6], optimizing likelihood functions with complex functional forms [7], many applications of bootstrapping or other randomization-based inference, network analysis [8], and Markov Chain Monte Carlo fits of hierarchical Bayesian models (e.g., [9]). Here, we focus on common issues with large databases and stochastic simulation models, applying general approaches for optimizing code to two simple examples:

1. Bootstrapping mean values 10,000 times in a moderately large dataset of 750 million records. This example is highly suited for parallel computation and employs common data protocols: indexing and grouping, resampling and calculating means, and formatting and saving output (Fig. 1A–C).
2. A simple stochastic two-species Lotka-Volterra competition model, which utilizes basic mathematical operations, randomly sampling statistical distributions, and saving fairly large simulation results. Additionally, as change depends on the state of the population in a previous time step (a Markov process), a single run cannot be conducted in parallel (Fig. 1D–F).

**Fig 2:**
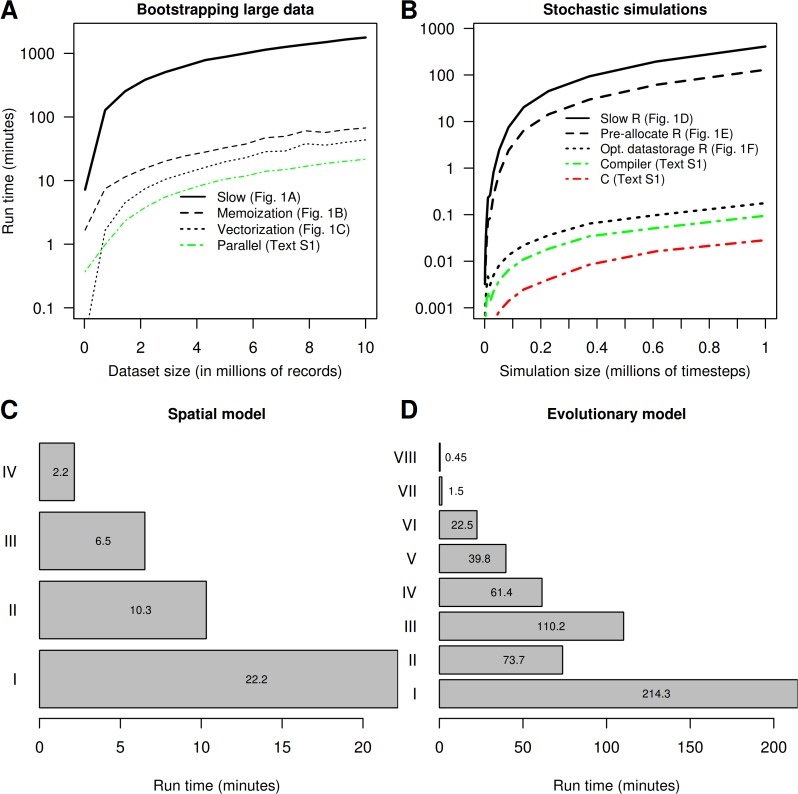
Execution time in minutes, required to complete various computational problems, using the optimization techniques discussed. Panels A and B show the execution time as a function of problem size for 10,000 bootstrap resamples conducted on datasets varying in size (A) and time required to run a stochastic population model against the number of time steps (B). "Naive" R code, in which no optimizations are applied, uses most computing resources (solid lines in A and B). Optimized R code, with use of efficient functions and optimal data structures pre-allocated in memory (dashed lines in A and B), is faster. In both panels A and B, the largest speed-ups are obtained by using optimal R code (black lines). Subsequent use of parallelism causes further improvement (dot-dashed green line) in A. In panel B, using R's byte compiler improved execution time further above optimal R code (dotted lines in green) while the smallest execution times were achieved by refactoring code in C (red dot-dashed lines). Panels C and D give the computing time (in minutes) needed to conduct the calculations from (C) Merow et al. [10] and (D) the calculations represented by Fig. 3 in Visser et al. [11]. Bars in panel C represent the original unaltered code from [10] (I), the unaltered code run in parallel (II), the revised R code where we replaced a single data.frame with a matrix (III) and the revised code run in parallel (IV). Bars in panel (D) represent the original unaltered code [11] (I), original run in parallel (II), optimized R code (III), optimized R code using R's byte compiler (IV), optimized R code run in parallel (V), optimized R code using byte compiler run parallel (VI), code with key components refactored in C (VII), and parallel execution of refactored code (VIII). All parallel computations were run on 4 cores, and code is provided in S1 Text, section 3, S2 and S3 Texts.

The optimization of these examples can be followed in detail in S1 Text. In all cases, we obtained speed-ups of 10.5 to 14,000 times with benefits that increase with the amount of computation (Fig. 2). Finally we show the relevance of these techniques when applied to two previously published problems concerning spatial models [10] and the analysis of fitness landscapes [11] (documented in S2 and S3 Texts).

## When (Not) to Optimize?

One should consider optimization only after the code works correctly and produces trustworthy results [12]. Correct code should be the primary goal in any analysis. Before optimizing, it is important to recall a fact that is recognized by programmers: “Everyone knows that debugging is twice as hard as writing a program in the first place. So if you're as clever as you can be when you write it, how will you ever debug it?” [13]. Optimized code may be faster but tends to lose robustness and generality, be more complex and less accessible, introduce new bugs, and have limited portability and maintainability. Loosely written code, in a high-level language, may be slow, but it will be faster to develop and easier to prototype. In concurrence, it is sensible to prioritize robust, general, and simple code above “fast code”—robust and general programs work in multiple situations (S1 Text: examples 2.14 and 2.15), are reusable, and hence save development time, while clear simple code saves time when revisiting old code (or when sharing among peers). Clearly, slower code will lead to lower total project time if it is more generally applicable, or when additional development and debugging time exceeds what is saved in run time. Therefore, before attempting to optimize code, one should first determine if it will be worthwhile.

## What to Optimize?

**Fig 3:**
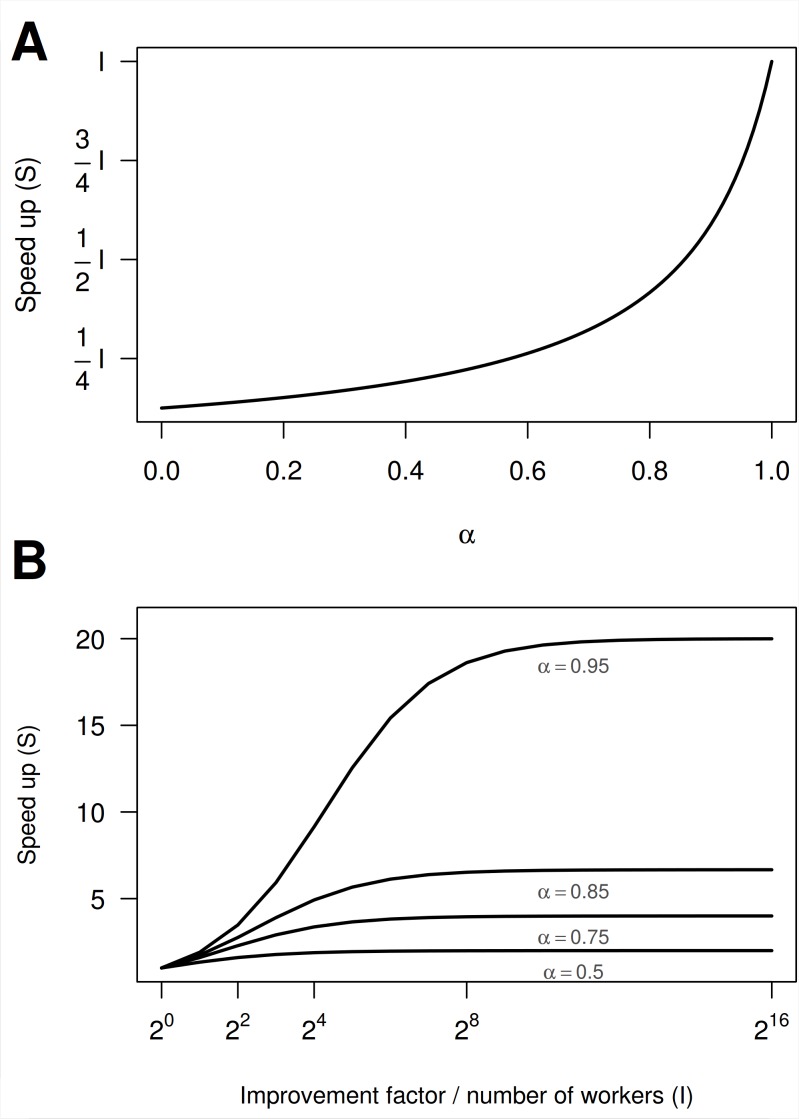
Projected improvements in total program run time using Amdahl's law. (**A**) Realized total speed up when a section of code, taking up a fraction α of the total run time, is improved by a factor *I* (i.e., the expected program speed-up when the focal section runs *I* times faster). We see that optimization is only effective when the focal section of code consumes a large fraction of the total run time (α). (B) Total expected speed-up gain for different levels of α as a function of *I* (e.g., the number of parallel computations). Theoretical limits exist to the maximal improvement in speed, and this is crucially and asymptotically dependent on α—thus code optimization (and investment in computation hardware) are subject to the law of diminishing returns. All predictions here are subject to the scaling of the problem (S1 Text, section 2).

Amdahl’s law (Fig. 3) [14] provides insight into the value of making a specific section of code more efficient: unless this code section uses a very large fraction of the overall execution time, the reduction in run time for the whole program may be modest. For example, consider code that requires 120 minutes to run, but one section can be sped up by a factor of 2. If that section consumes 95% of the original run time, optimization will improve total run time to 64 minutes. If that section consumes only 50% of the original run time, total run time will only improve to 90 minutes (Fig. 3A). Amdahl’s law also shows that increased effort in optimization has diminishing returns (Fig. 3B).

Empirical studies in computer science show that small sections of code often consume large amounts of the total run time [15]. Identifying these code sections allows effective and targeted optimization. “Code profilers” are software engineering tools that measure the performance of different parts of a program as it executes [16]. When dealing with large data sets or large matrices, where memory storage is limiting, memory profilers (e.g., *Rprofmem*) provide statistics to gauge memory efficiency. We illustrate the value of profiling in S1 Text, sections 1–3, using R’s profiler (*Rprof*) and a newly developed R package (*aprof*: “Amdahl's profiler”). This package helps to rapidly (and visually) identify code bottlenecks and potential optimization gains (as illustrated in Fig. 1).

## How to Optimize?

After bottlenecks have been identified, the precise nature of any optimization depends on the specific properties of the programming language. However, generally large gains can be achieved by avoiding common inefficiencies. (See S1 Text, section 1.4 for more background information.)

### 1) Nonessential operations

Eliminating unnecessary function calls, printing statements, plotting, or memory references can increase efficiency. Many functions in high-level languages (see below) like R have default options enabled that may incur unnecessary cost. When profiling identifies a specific function as a bottleneck, check its inputs. For example, using unlist() on a list with named vectors can be sped up considerably with use.names = FALSE, while loading large datasets with read.table() or read.csv() is expedited by setting the colClasses input.

### 2) Memoization

Store the results of expensive function calls that are used repeatedly. For instance, transpose a matrix or calculate a mean once prior to entering a loop rather than repeatedly within a loop. Replacing the repeatedly recalculated mean(d) in line 10 of Fig. 1A, with an object “avg” to store the mean of d, results in a drastic improvement in efficiency with a speedup of ∼ 28 times (red lines in Fig. 1B).

### 3) Vectorized operations

Writing a loop to calculate elements of a vector or rows of a matrix is inefficient. In R, vectorized functions are faster because the actual loop has been pre-implemented in a lower-level, compiled language (in most cases C; [17]). Replacing the operation of calculating the mean differences over columns in lines 8–10 of Fig. 1B with its vectorized and highly specialized equivalent “colMeans(d[index,])-avg” (Fig. 1C, line 7), the overall execution speed is improved an additional 1.4 times. Note that very large vectors will be inefficient in R. In those cases chunk-based iteration is an effective compromise (see section on large data below).

### 4) Growing data

“Growing data” refers to adding values incrementally to data frames, matrices, or vectors. When a new value is added and the object is lengthened, the new, longer, object must be written to free space in the memory. In the next iteration this process repeats itself, becoming ever more time-consuming. It is much faster to pre-allocate memory that is sufficiently large for the final object than to fill in new values as they are computed. Replacing line 16 from Fig. 1D with a pre-allocate-and-fill operation (lines 6, 7 and 17 in Fig. 1E) results in an ∼ 5 times speed-up.

### 5) Dispatch overhead

Another potential speed-up strategy is to create custom functions to avoid overhead in base- or package-provided functions. The object-oriented philosophy of R encourages general purpose functions; these perform a large number of checks prior to doing the desired task. Custom-written functions perform only the desired task, without these checks, and can lead to significant speed-ups. Another strategy would be to use lower-level functions (see S1 Text, section 1.4) instead of their default counterparts (e.g., *lm*.*fit* vs *lm)*. Note that custom and lower-level functions should be used cautiously as they provide speed at the cost of requiring much stricter compliance to input rules (e.g., S1 Text: examples 2.14–2.15). For example, the Lotka-Volterra competition model code in Fig. 1F stores results in a data.frame. In R, data.frames are used for storing multiple types of data (e.g., integers, characters, factors etc.) however this functionality is not needed when only using numeric data. Switching to an efficient way of storing a single data type (a matrix) speeds up computation by a factor of ∼20 (compare Fig. 1E,F, Fig. 2B).

After each optimization step confirm that new code versions produce identical results compared to previous slower versions. Some simple functions for formal results checking in R include *identical()* and *all*.*equal()*.

### Parallelization

Parallel computing divides calculations into smaller problems and solves these simultaneously, using multiple computing elements (hereafter “workers”). In the biological sciences, many computationally intensive problems are “embarrassingly parallel” [18], where almost all calculations can be completed in parallel. Common examples are Monte-Carlo simulation and bootstrapping (S1 Text, section 3). Popular parallel computing systems include computations on single multi-cored machines or “distributed computing” on clusters of workstations connected via a network. Our focus here is on modern multi-cored machines, where parallel computing has become relatively easy to implement, and which most people have access to—though we highlight where distributed computing will be particularly useful. Users should note before implementing a parallel algorithm that parallel code can be more challenging to debug. Accordingly, a handful of basic rules are worth reviewing (details in S1 Text: section 3):

1. There is a start-up cost to initializing a collection of jobs to run in parallel, so a collection of small jobs may run faster sequentially (e.g., Fig. 2A), and more parallel processes do not necessarily lead to faster program execution [5] (i.e., parallel algorithms are also subject to Amdahl’s law, see Fig. 3B). When finalizing a parallel run, results need to be copied back to the parent process and collated from each worker; this can be expensive, especially when results are large.
2. In most computing devices, random access memory (RAM) is shared among parallel processes [17]. Ensure that enough memory is available for each worker, so parallel workers do not have to wait for memory to become available. Because shared memory decreases geometrically with each added worker, such systems are unsuited for big data. Parallel computing on a cluster, where memory is distributed (i.e., increases proportionately with the number of threads), or an algorithm that partitions the data proportionally to each worker, will be more feasible.
3. Independence of random number sequences must be ensured for valid scientific results (e.g., [18,19]). Ensure that random numbers sequences are unique, reproducible, and will not overlap (examples in S1 Text, section 3.4).
4. Avoid load imbalances, where one processor has more work than the others causing them to wait. Attempt to split jobs equally. This is especially challenging on a cluster where jobs should match the available resources on each host machine.

Starting with the optimized but serial R-bootstrap code (Fig. 1C) we created a parallel algorithm for use on a single machine (S1 Text, section 3), with which we achieved a speed-up by a factor of 2.5 with 4 cores (Fig. 2A).

### Calling Low-Level Languages

Parallel computing can reduce run time, but it essentially does not make code run any faster. In other cases parallel computing may not be possible (e.g., Fig. 1D–F). Substantial improvements in execution time can still be made by rewriting key sections of code in a “lower-level” or compiled language. Beginning R-programmers with limited familiarity with compiled languages are advised to pursue other “R-specific” routes of optimization first. These are more straightforward and lead to the greatest relative speed-ups (Fig. 2), while C is more complicated to develop and debug (requiring memory management and missing data (NA) handling).

In general, there are two types of programming languages: interpreted (R, MATLAB) and compiled (C, Fortran). In interpreted languages, like R, code is indirectly evaluated by an evaluation program (hereafter the R-interpreter; [12]). In compiled languages, like C, code is first translated to machine language (i.e., machine-specific instructions) by a compiler program and then directly executed on the central processing unit (CPU). The differences in the type of programming language used can have large effects on execution speed [12].

Compiled and interpreted languages exhibit a trade-off in run time versus programmer time, respectively. Interpreted languages have the benefits of being relatively easy to understand, debug, and alter. However, there is usually much higher CPU overhead as each line must be translated (i.e., “interpreted”) every time it is executed. Compiled languages tend to be more challenging to code and debug, but are highly efficient when executed, as “translation overhead” occurs just once, when the source code is compiled.

In the Lotka-Volterra code in Fig. 1F, we find no clear bottlenecks, with most time consumed by the repeated interpretation of mathematical operators (*, +, etc, S1 Text, section 6.15) and random number generation (“rnorm”). We were able to remove such translation overhead by rewriting critical parts of the program in in C and calling the compiled code from R. With this we created a six-times–faster “vectorized” version of the model (Fig. 2B). In S1 Text (section 5) we give practical advice on extending R with C using the most common interfaces for extending R (through the .C and .Call interfaces; [19]). In S3 Text, our applied example, we use *Rccp* [20] and *RccpArmadillo* [21] to speed up a matrix-multiplication by a factor of 400.

Many interpreted languages also provide special compilers for finished programs, which are simple to use. These represent a compromise between a true compiler and an interpreter. In the R *compiler* package a byte-code compiler is used, which translates R code into more compact numeric codes. It does not produce machine-language code, but instruction sets designed for efficient execution by the interpreter. This may be a quick fix to speed up some code, but most functions are already distributed in byte-compiled form, so further speed gains using byte-compiling are modest. In our examples, we did find that using this compiler decreases execution time (Fig. 2B and S1 Text, section 6.5.1).

### Large Data

R loads data into memory by default: datasets comparable in size to the amount of memory available will slow R to a crawl while datasets exceeding the memory space will fail altogether. In these cases researchers can either 1) use databases stored outside R, accessing these in R via languages like SQL (via, for example, RSQLite) or 2) use more memory-efficient algorithms. The latter usually involves sequential algorithms, which restrict memory usage to one block of data at a time. Many statistics can be calculated sequentially (e.g., [22]), but problems will take longer to solve as accessing data from a storage disk is slower than from memory. We provide a short example on how to do this for the bootstrap example in S1 Text (section 4), using the *ff* package [23].

### Using More Efficient Algorithms

A final method to speed up computations is to use a more efficient algorithm. These are mathematically equivalent, but computationally smaller, methods (i.e., they use fewer operations). Although this is highly problem specific, we nevertheless highlight this point, as it is worth scrutinizing the efficiency of the algorithm in use since substantial speed-up may be gained when alternatives exist [2]. For example, matching m values in a table of n elements requires on the order of m × n operations with a loop and on the order of m + n options when a hash table is constructed first [12]. Subsequent matches will be even faster if the hash table is stored, as in the *fastmatch* library [24]. Additional examples include using the turning bands algorithm [25,26] instead of a Cholesky (variance-covariance) decomposition when simulating a large spatially correlated random field or using an algorithm like Broyden-Fletcher-Goldfarb-Shanno in non-linear optimization, which requires fewer evaluations of the objective function because the Hessian matrix is built up from information about the first derivatives.

## Recommendations

Optimizing code can provide efficiency gains of orders of magnitude, as our benchmark results show (e.g., Fig. 2). However, we do not recommend optimizing immediately. Realize that one will inevitably sacrifice clarity, generality, and robustness for speed. At the start of a project, the most productive approach (e.g., [3]) is often to write code in the highest-level language possible ensuring the program runs correctly. High-level languages enable rapid decision-making and prototyping, and correct code enables checking of more optimized versions. When a performance boost is deemed worthwhile, for example, through profiling, only optimize those parts identified as bottlenecks to avoid sacrificing development time in favour of optimization [3,12]. The primary route for optimization should be efficient R code which, as we show in Fig. 2, yields the largest gains for the least effort.

The fastest running code examples shown here are the instances where we called compiled code from R (Fig. 2, S1 Text: section 6). This technique is especially powerful when one can use the vast libraries of algorithms that already exist in C (and Fortran), which are often optimized and efficiently coded [12]. However, a programming language like C has a steeper learning curve and when learning C requires too much time, we encourage biologists to collaborate with computer scientists in their research or to include contracts for computational consultation in grant budgets [3].

## Conclusion

Learning how to program and efficiently use computational resources is not only convenient. Computing has become fundamental to the practice of science (e.g., [1–3,27]). In biology, research is striving toward ever more accurate projections to inform public leaders on nature management or make predictions regarding how ecosystems respond to change (e.g., [28–30]). More often than not, such accurate predictions will require high levels of detail as natural systems are variable and include intricate levels of biotic and abiotic interactions (e.g. [31–32]). With these challenges ahead, the use of computationally intensive analyses in the biological sciences should not be constrained by programming practices.

## Supporting Information

S1 Text Tutorial with background information and detailed examples on, e.g, profiling, optimal R coding, parallel computation, working with large datasets, and extending R with C. (PDF)

S2 Text Optimization of code from [10]. (PDF)

S3 Text Optimization of code from [11]. (PDF)
