# Supplementary material for: Speeding Up Ecological and Evolutionary Computations in R; Essentials of High Performance Computing for Biologists
Source: PLoS Comput Biol. 2015 Mar 26;11(3):e1004140. doi: 10.1371/journal.pcbi.1004140 (PMC4374948; doi:10.1371/journal.pcbi.1004140)
Supplement: S1 Text — (PDF) [file pcbi.1004140.s001.pdf]

S1 Text: Speeding up ecological and evolutionary computations in R; essentials of high performance computing for biologists.

Marco D. Visser*[1,2], Sean M. McMahon†[3], Cory Merow‡[4,5], Philip Dixon§[5], Sydne Record¶[6,7], and Eelke Jongejans‖[1]

[1]Departments of Experimental Plant Ecology and Animal Ecology & Ecophysiology, Radboud University Nijmegen, the Netherlands
[2]Smithsonian Tropical Research Institute, Panama
[3]Smithsonian Environmental Research Center, Edgewater, USA
[4]Department of Ecology and Evolutionary Biology, University of Connecticut, USA
[5]Department of Statistics, Iowa State University, USA
[6]Department of Biology, Bryn Mawr College, USA

January 28, 2015

Contents

1 Code optimization in R **3**
1.1 Introduction . . . 3
1.2 General guidelines for optimization . . . 5
1.3 Profiling . . . 6
1.4 What slows down computation in R . . . 12
1.4.1 Dispatch overhead and nonessential operations . . . 12
1.4.2 Vectorization . . . 13
1.4.3 Growing data . . . 14
1.4.4 An interpreted language . . . 15

*m.visser@science.ru.nl
†mcmahons@si.edu
‡cory.merow@gmail.com
§pdixon@iastate.edu
¶srecord@brynmawr.edu
‖e.jongejans@science.ru.nl

2 Optimizing R code: a bootstrap example **17**
2.1 Bootstrapping a moderately large biodiversity dataset 17

3 Parallel computing **34**
3.1 Parallel computing in R 35
3.2 Parallel calculations using forking (Unix systems including Mac) 35
3.3 Number of workers 35
3.4 Random numbers 36
3.4.1 The parallel bootstrapping algorithm via forking 39
3.5 Alternative parallel bootstrapping algorithm (including Windows) 40
3.6 The end gains 42
3.7 Closing remarks 42

4 Bootstrapping large data in R **44**

5 Extending R: High performance with compiled code **48**
5.1 Getting started with extending R 48
5.1.1 Getting started under Windows 48
5.2 Writing C code 49
5.2.1 C coding in a nutshell 50
5.3 Compiling C code and creating shared libraries 52

6 Calling C from R: Optimizing a population model **56**
6.1 A stochastic 2-species population model 56
6.2 The final step: the population model in compiled code 70
6.2.1 Working with R objects 71
6.2.2 SEXPTYPES 72
6.2.3 Random number generators 74
6.2.4 The final model 77
6.3 The end gains 80
6.4 Summary 81
6.5 Useful tools 82
6.5.1 The R Byte Compiler 82
6.5.2 Rccp 83
6.5.3 Profilers for compiled code 83
6.6 Closing remarks 84

1 Code optimization in R

1.1 Introduction

The use and advancement of mathematical and computational tools in ecology has seen considerable headway since its pioneering days (Lotka, 1925; Volterra, 1926; Gause, 1934). Today, computation and computer code are fundamental parts of our science and the computational needs of biologists are extensive (Fig. 1.1); emerging fields such as ecoinformatics and computational ecology (see e.g. refs [1,2] in the main text) bear witness to the fact that biologists are collecting and distributing data on larger scales than ever before (e.g. GenBank, NEON; Jones *et al.*, 2006; Kelling *et al.*, 2009) Such data sources are used to inform, validate and construct ever more elaborate models (e.g. Clark *et al.*, 2010; Nathan *et al.*, 2011; Luo *et al.*, 2011). These complex models are often analytically intractable and require intensive numerical calculations (e.g. refs [31] in the main text).

In such an intense setting, computational issues should not limit the scope of the science conducted. That is why we decided to compile this guide for improving computational efficiency in a wide variety of settings. Here, we provide background information on the topics discussed in the main paper. We review the scattered information on computational efficiency found online, in text books and other sources, and provide an overview which includes some basic theory from computer science, but is mostly a hands-on tutorial for learning how to identify bottlenecks and speed-up the corresponding R code. We hope to show that fast and efficient code is often straightforward and easily implemented.

In the examples to follow, we will focus solely on R (R Core Team, 2013) [1] . Using some realistic programming examples from biology, we give guides on profiling, general inefficiencies in R, parallel computing and extending R with C.

[1] We feel that it makes sense to focus on techniques that are directly applicable to R because of its widespread adoption in the biological sciences. This greatly facilitates code sharing as such widespread use is leading to a reverse "tower of babel" situation, where ecologists are moving away from a diverse suite of programming languages towards speaking a common computational tongue.

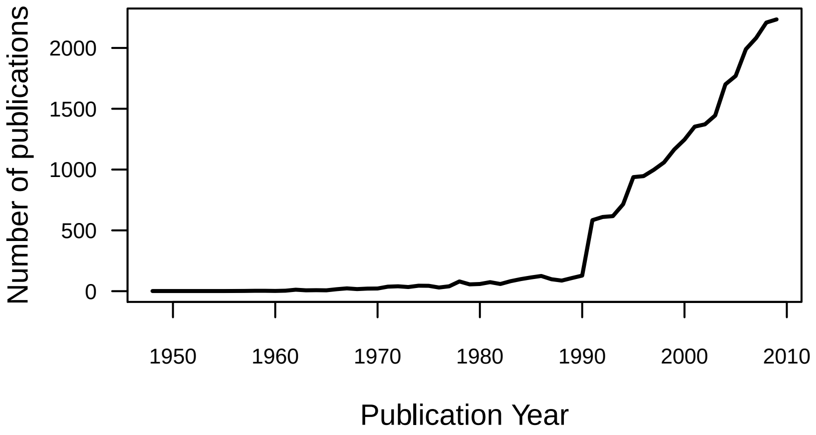

Figure 1.1: The rate of increase in computationally intense problems in biology. The vertical axis denotes the number of publications for a given year. Starting slowly in 1950 with the advent of computers, there has been a consistent exponential increase in computational power, leading to a true explosion of computationally heavy research during the past 20 years in virtually every research topic in ecology and evolutionary biology. Data was obtained through the Web of Science, and includes the fields of biology, ecology, evolutionary ecology, fisheries and plant sciences. The search was conducted to include all papers with at least one of the following keywords: Stochastic simulations, Markov chain Monte Carlo, agent based model, individual based model, Monte Carlo, bootstrap, randomization, simulation.

All code examples and benchmarks in this document were run on a single test machine (MSI model gt680R, with CPU Intel i7-2639QM, 8GB DDRII RAM running Fedora 17), code was also tested separately on other systems (Windows and Mac). This should ensure that code examples supplied here work on all platforms (unless indicated separately). We assume that you have a working R installation (R > 3.1.0), and have modified little to nothing about the installation yourself [2]. We also supply each code example as an online textfile (indicated by the "OnlineRcodeXX.R" comment within each code box), for users who prefer to work with textfiles [3].

1.2 General guidelines for optimization

Learning how to code neatly and efficiently is key to solving complex computational problems quickly and in the long-run we feel that everyone will be better off learning the fundamentals of efficient coding (see programming guides for scientists, e.g. ref [3] in the main text). In general, however, the following steps are advocated by computer scientists when attempting to optimize code (refs [3,12] in the main text). They are meant to ensure that you are working correctly and productively. We will follow these steps throughout this document.

1. Your first objective should always be to make sure that your program does what you intended it to do correctly. Start by using easy to debug R code, without worrying too much about efficiency initially. Once you have a working program with results you can trust, you have a solid basis to compare more complex optimized code against.

2. If you believe performance should and can be improved, start profiling your program (as shown in section 1.3) or a smaller version of your program. This will help you to discover what is using most of the resources. Find out whether it is worthwhile to spend time optimizing using Amdahl's law (see the main document) to calculate your expected gains (we use our R package *aprof* for this).

3. Once you find bottlenecks, and are certain that optimizing your code is worthwhile, you should start by asking yourself if you can speed up your

[2] A number of replacement "packages" can be downloaded off the web, including optimized replacements of your "BLAS" libraries (for matrix operations) to entire replacements for R such as pqR. The good news is that the techniques shown here, will also work well when you are using such packages. However, if you have installed any general all purposes speed-up packages, we advice you to take caution especially with parallel code. In general, unless you know exactly what you are doing, or more specifically what these packages are doing, you may end up unwittingly paralyzing your code. Indeed, if you are unaware that a package automates the parallel computations for you, you may end-up running its parallel operations within your own parallel script. This quickly leads to over-parallelism (multiple threads within multiple threads), causing CPU's to park threads and increasing execution time to extremes. It is our experience that in many cases you can achieve speed-ups beyond what these packages provide, with directed and focussed optimization - focusing on the unique bottlenecks of your program will, generally speaking, be far more effective

[3] Download these from https://github.com/MarcoDVisser/SpeedUpR

R code itself (section 1.4). In most cases looking for R-specific solutions will yield the greatest speed-ups with minimal effort. For example, are you conducting any unnecessary operations? Is there something that you can vectorise? Can you drop a higher level function with more overhead for a lower level one (e.g. lm vs lm.fit)? Can you replace a slow function with a custom one?

4. If you are still not satisfied with the program's performance, then ask yourself if your code can run in parallel (section 3)? Or you may be able to rewrite certain key parts in C, C++ or Fortran (section 5).

5. At each step, repeatedly confirm for yourself that each version of code is giving the same answers as the correct, but slower, R code.

1.3 Profiling

Identifying which part of the code takes the most time to run can allow effective and targeted optimization efforts. "Code profilers" help towards this end, which is why we describe how to use a statistical profiler (called *Rprof* in R) next. R's statistical profiler uses "operating system interrupts" to probe which routines are active at regular intervals, counting which R expressions are consuming the most resources. For example, profiling a program consisting of two commands will tell you exactly how much time is spent in each command, and therefore which of the two you should take a closer look at.

To help organize the sometimes confusing output from the R profiler, we have developed software in the form of an R package (*aprof*: "Amdahl's profiler") which uses visual tools to identify bottlenecks (as illustrated in Figure 1.2). This makes it much easier to rapidly identify bottlenecks in your program code. An example of using *aprof* is given below. The package is available at CRAN. In cases where random-access memory (RAM) storage, rather than processor time, poses the most pressing limit, one can use a memory profiler (e.g. Rprofmem) to obtain similar statistics for memory efficiency.

Simpler, though less informative, tools to time and track resource use include the R functions system.time() and object.size(). The former returns the time used by both R and the operating system (for communication between devices, file writing, etc.), while the latter gives an approximation of the memory usage of objects. These can be useful when you only want a rough idea of resource use, and don't want to go through a complete profiling exercise.

To use the R profiler, we first need to make a simple program which we can then profile. Here is an example of a program (InterpreterQuirks) that executes the calculation $N/(1+N)$ many times with either parentheses or brackets, and different amounts of each. The function highlights some of the quirks of an interpreted language as explained in section 1.4.

```
## OnlineRcode01.R

InterpreterQuirks<-function(N){
  for (i in 1:N) { N/(1+N) }
  for (i in 1:N) { (((N/(1+N))))}
  for (i in 1:N) { N/{1+N} }
  for (i in 1:N) { {{{N/{1+N}}}} }
}

## Save the function to a source code file
dump("InterpreterQuirks",file="InterpreterQuirks.R")
```

Next we use *Rprof* to start profiling *InterpreterQuirks*. As we will be using "line profiling" we will first reload our saved file. This ensures that the version in R and the saved file on the disk match up exactly (the function "*dump*" used above can sometimes change the layout slightly).

```
## OnlineRcode02.R

source("InterpreterQuirks.R")
```

Then we switch on R's profiler *Rprof*, and because we want to know which lines of *InterpreterQuirks* are the slowest, we need to make sure the option *line.profiling* is set to TRUE. Here we use a time interval between samples of 0.02.

```
## OnlineRcode03.R

Rprof(file="InterpreterQuirks.out",interval = 0.02,
          line.profiling =TRUE)

InterpreterQuirks(N=1e5) # run 100 thousand times

Rprof(append=FALSE) # stop profiling
```

Now lets summarize the profiling output with the function *summaryRprof*:

```
## OnlineRcode04.R

summaryRprof("InterpreterQuirks.out")

## $by.self
##                     self.time self.pct total.time total.pct
## "InterpreterQuirks"      0.34    89.47       0.38    100.00
## "/"                      0.04    10.53       0.04     10.53
##
## $by.total
##                     total.time total.pct self.time self.pct
## "InterpreterQuirks"       0.38    100.00      0.34    89.47
## "/"                       0.04     10.53      0.04    10.53
##
## $sample.interval
## [1] 0.02
##
## $sampling.time
## [1] 0.38
```

This gives us information on the program run time, and the percentages of time various functions took, identifying which function call is taking the most time. However, in this example such output is not very informative as functions or operators are repeated in multiple lines of code.

Here we would rather visualize the time spent in each line of code, and therefore we need to load our package *aprof* ("Amdahl's profiler"). Next we will use *aprof*'s standard plot function on our program *InterpreterQuirks* (see ?*plot.aprof* for details). However, before we start we need to make an "*aprof object*" using the function *aprof*. This object will contain the profiling information and the source file "*InterpreterQuirks.R*".

```
## OnlineRcode05.R

require(aprof)

## Loading required package:  aprof

##make an object of the aprof class
IntQuirksAprof <- aprof("InterpreterQuirks.R","InterpreterQuirks.out")
```

Now that we have a standard "*aprofobject*" we can display some basic information about the profiling exercise by simply typing the name of the

"*aprofobject*" and hitting return. In contrast to the *summaryRprof* which focuses on specific functions, this gives you information on which lines of code took the most time to execute.

```
## OnlineRcode06.R

IntQuirksAprof

##
## Source file:
## InterpreterQuirks.R (7 lines).
##
##  Call Density and Execution time per line number:
##
##       Line  Call Density  Time Density (s)
## [1,]  3     4             0.08
## [2,]  5     4             0.08
## [3,]  6     5             0.1
## [4,]  4     6             0.12
##
##  Totals:
##  Calls  20
##  Time (s)  0.46  (interval =   0.02 (s))
```

Next we can use the standard *plot* function on this object to display the execution time per line. The following code should return figure 1.2.

```
## OnlineRcode07.R

plot(IntQuirksAprof)
```

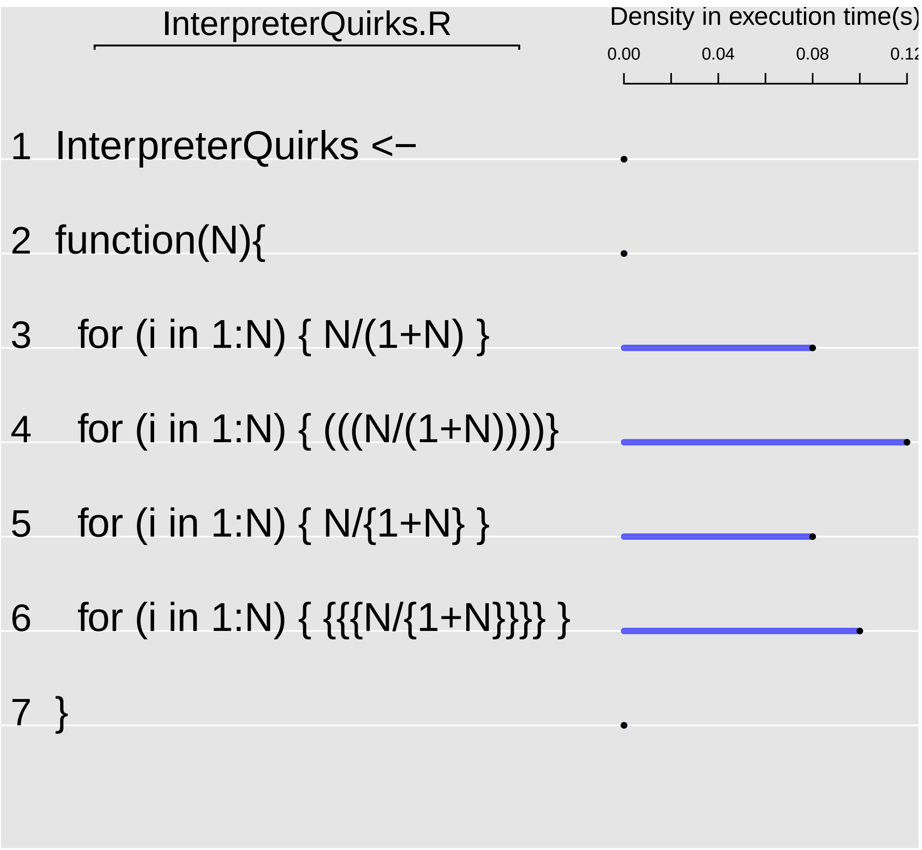

Figure 1.2: Output from *aprof*, showing the source code for our function *InterpreterQuirks* in the left panel with the execution time per line of code in the right panel. We see a classical interpreted language problem, where more parentheses and brackets add little to the program, but ensure that the R interpreter has to evaluate more expressions. We also see that some symbols (here "{" and "}") are interpreted faster (i.e. the lookup speed for these symbols is likely faster or they infer less "overhead").

Plotting an *aprof* object is useful when your program is relatively small however, when your code consists of hundreds of lines, a better function would be *profile.plot*. Go ahead and use it on our *aprof* object "IntQuirksAprof" to see what it does (type ?*profile.plot* in the command line for details). Another useful feature is to summarize our *aprof* object, which gives us the theoretical maximum attainable speed-up for each line of code (see ?*summary.aprof* for details).

```
### OnlineRcode08.R

summary(IntQuirksAprof)

## Largest attainable speed-up factor for the entire program
##
##         when 1 line is sped-up with factor (S):
##
##   Speed up factor (S) of a line
##            1     2     4     8     16    S -> Inf**
## Line*: 4 :  1.00  1.15  1.24  1.30  1.32  1.35
## Line*: 6 :  1.00  1.12  1.19  1.23  1.26  1.28
## Line*: 3 :  1.00  1.10  1.15  1.18  1.19  1.21
## Line*: 5 :  1.00  1.10  1.15  1.18  1.19  1.21
##
## Lowest attainable execution time for the entire program when
##
##              lines are sped-up with factor (S):
##
##   Speed up factor (S) of a line
##            1       2       4       8       16
## All lines   0.4600  0.2300  0.1150  0.0575  0.0288
## Line*: 4 :  0.4600  0.4000  0.3700  0.3550  0.3475
## Line*: 6 :  0.4600  0.4100  0.3850  0.3725  0.3663
## Line*: 3 :  0.4600  0.4200  0.4000  0.3900  0.3850
## Line*: 5 :  0.4600  0.4200  0.4000  0.3900  0.3850
##
##     Total sampling time:  0.46  seconds
##  *  Expected improvement at current scaling
##  ** Asymtotic max. improvement at current scaling
```

Using this information we can easily decide where to focus our efforts and, maybe more importantly, decide whether it is worth the effort to optimize the code in the first place. These numbers effectively tell us what the predicted overall speed-up of the program would be when we focus on a single line. As we can see in the uppermost table, line 4 would be the most promising to work on, as it shows the greatest improvement for each of the sets of speed-up factors (1 - 16×). That is, if we improve the execution time of a given line by a factor S (S times faster), the table predicts how much this improvement

will affect the overall run-time of the entire program. In the above example we see, however, that the gain is minimal and even when the speed-up factor goes to infinity (effectively when the run time of that line becomes 0; $\lim S \to \infty$) we can only achieve a maximum speed-up of slightly over 1.3. Or in other words, if we were to infinitely improve the code in line number 4 we would only improve the overall program by slightly more than 30%. Infinitely faster is not something we are likely to achieve, but also for the more practical speed-up factors we also see that a factor of 16 improvement is hardly an improvement over a factor of 4. In such cases it may not be worthwhile to either purchase computing resources (parallel machines or execution time on a cluster) or spend time optimizing code. Naturally, in this simple example the overall execution time is so small that we won't spend time optimizing it. However, as we usually would profile a simplified version of a larger program where the execution time may be considerably larger, even a small improvement in execution time may be worthwhile (see the bootstrap problem below).

1.4 What slows down computation in R

1.4.1 Dispatch overhead and nonessential operations

A major benefit of statistical software like R is that it employs a wide variety of statistical tools and programs that work "out of the box". These predefined programs often contain additional features meant to ease their use by auto-formatting data, error checking, and making other checks on user inputs. When dealing with computationally onerous problems these features can cause considerable slowdowns. One potential speed-up strategy is therefore to create custom functions to avoid overhead in base- or package-provided functions. These custom functions can be then be "specialized" to perform only the desired task, and can lead to significant speed-ups. For example, in Fig. 1.3 (i & j) we see that using R's "*mean()*" function is less efficient than a more basic function with the same result:

```
## OnlineRcode09.R
avg <- function(x,...){
  sum(x,...)/length(x)
}
```

The additional features in "*mean*" function ("dispatch overhead") make it slower, but more robust and easier to use. When creating such custom functions one should realize that much of the original robustness will be lost in the more basic function. Which, as pointed out by an anonymous reviewer of this document, can result in serious errors and incorrect results:

```r
## OnlineRcode10.R
x<-c(1, 2, NA)
DefuaultMean<-mean(x, na.rm=TRUE)
CustomMean<-avg(x, na.rm=TRUE)
identical(DefuaultMean,CustomMean)

## [1] FALSE
```

Another strategy to avoid overhead would be to use lower-level functions instead of their default counterparts (e.g. *lm.fit* vs *lm*). For instance, if the function lm is taking most of the time in your calculations, then you can use its lower-level function $lm.fit$. This lower-level function is much faster. However, lower level function should be used cautiously as they are again far less robust to bad user input and require much stricter compliance to input rules. You can often find such lower-level functions by looking at the source code of a "slow" function (using e.g. *page*("*lm*") is one way of doing this). A general guide to find the source code of a function (and any lower-level functions) is given in Ligges (2006).

1.4.2 Vectorization

Loops in code exacerbate inefficient code contained within the loop, and can easily prove to be one of the most basic processing bottlenecks. A valuable feature of R is vectorization, which can be used to avoid the inherent inefficiency of looping. Vectorization involves writing functions that are designed to work on vectors of values in one call. Researchers switching from a different language may at first construct loops that apply a function over a vector (e.g., when adding a series of numbers [a vector] to a single number, one could loop through the series adding each in turn). For instance, in ecological analyses it is often necessary to create a matrix of random numbers (e.g., null model tests for species co-occurrence). If we were to fill in random numbers in a large matrix using a double loop (example 1.1), this would be approximately 38 times slower than a vectorized approach (example 1.2 creating random sequences for entire rows). Avoiding loops altogether in R, by directly filling the appropriate sized matrix (example 1.3) is approximately 63 times faster than the double loop (1.1).

R Example 1.1.

```r
## OnlineRcode11.R


mat <- matrix(ncol=1e3,nrow=1e3)
```

```
for(i in 1:1e3) {
        for(j in 1:1e3) {
        mat[i,j] <- runif(1) } }
```

R Example 1.2.

```
## OnlineRcode12.R

mat <- matrix(ncol=1e3, nrow = 1e3)
for(i in 1:1e3){ mat[i, ] <- runif(1e3) }
```

R Example 1.3.

```
## OnlineRcode13.R


mat <- matrix(runif(1e6), nrow = 1e3, ncol = 1e3)
```

The vectorized version is faster because creation of a long vector of random numbers though a loop is already implemented in a lower-level language (in most cases C; Schmidberger *et al.*, 2009). Vectorization in R is essentially a shift to a lower-level function when the user applies a vectorized function (e.g. functions like *runif*, *sum* and many others). This is far more efficient than using the R interpreter to loop through each instance, and calculations conducted in a vectorized fashion are thus often an order of magnitude faster (example 1.1. v.s. 1.2 v.s. 1.3). Some useful and highly optimized vectorized functions include: *cumsum, diff, rowSums, colSums, rowMeans* and *colMeans*. Note that example 1.2 can also be replaced with a single line of code using the "*apply*" family of functions; "*mat = apply(mat,1, function(X) runif(length(X)))*". In this instance, however, apply is not necessarily faster than a vectorized for loop (it is a myth is that the *apply* family of functions are always faster than for loops). An easy alternative to loops for beginning programmers in R is the function $Vectorize$ which is able to vectorize almost any function in R.

1.4.3 Growing data

Another inefficient coding practice is "growing data", where data frames, matrices, or vectors are created without defining their size a priori, and values (e.g. population states) are added to them incrementally in a loop.

R Example 1.4.

```
## OnlineRcode14.R


vec <- NULL
for(i in 1:n) vec <- c(vec, runif(1))
```

When growing data, instances of memory allocation and run time are significantly increased. In each iteration, the current amount of space dedicated to vec is too small to store the new version of vec (i.e. *c(vec, runif(1))*). Hence, an entirely new object must be written to free space in the memory for each item added. In the next iteration the space will be too small again and the process repeats itself becoming ever more time consuming. A better practice is to pre-allocate space in memory with placeholders. For instance:

R Example 1.5.

```
## OnlineRcode15.R


vec <- numeric(n)
for(i in 1:n) vec[i] <- runif(1)
```

Here the code numeric(n) creates a vector of the correct size n in the memory before values are assigned. Using memory profiling, we found that example 1.4 performs twice as many memory allocations (1890) as example 1.5 (820), with the latter being 8 times faster on average (when n = 1000).

1.4.4 An interpreted language

R is an interpreted language, which mean that code is evaluated by an evaluation program (hereafter the R-interpreter, see ref [12] in the main text). This causes CPU overhead as each line must be translated (i.e. "interpreted") every time it is executed. A classic example of translation overhead can be seen in Fig. 1.3 (a-h), which shows the execution time of two sets of four identical functions that simply evaluate the quantity N/N1 (code in section 1.3), only differing in the number of parentheses [4] and whether the language was compiled or interpreted.

[4] This is an example that has been used in the R community to show the quirks of an interpreted language

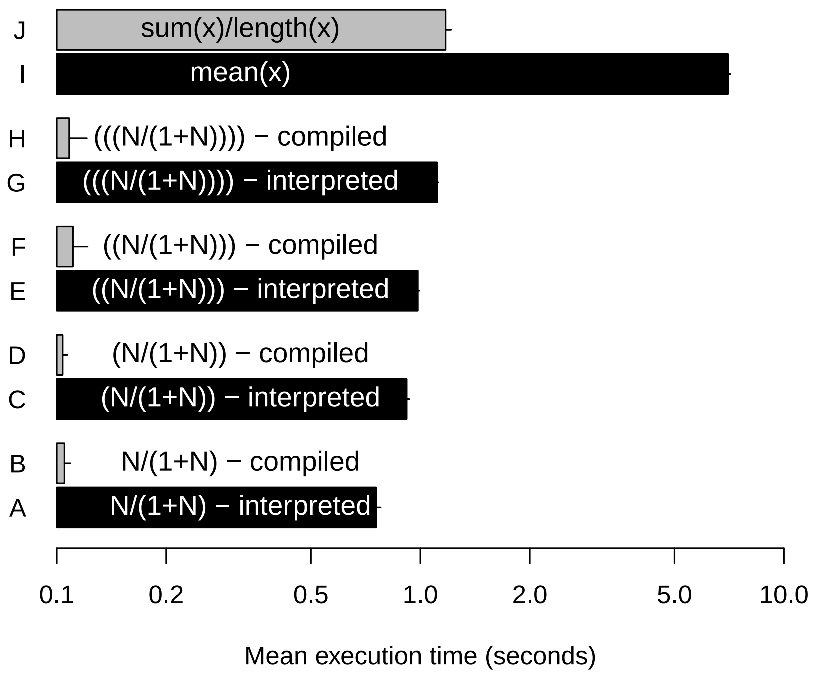

Figure 1.3: Execution time (on a log scale) for pairs of functions that differ in coding, but return exactly the same answer. Functions in bars a,c,e,and g (black bars) were run within a for loop 1 million times and differ in the number of parentheses used (example adapted from Radford Neal, University of Toronto). Functions in b, d, f and h (grey bars) are identical to a, c, e, and g but were coded in C and compiled. The examples in a, c, e and g show that adding additional parentheses "()" can noticeably influence execution time when using the interpreted language R. Here adding extra parentheses slowed down execution time by 15% per added parenthesis pair. Compiled code did not suffer from the same quirks, and is on average almost 8.2 times faster (examples b, d, f and h). Functions j and i calculate mean values for 1 million random vectors of length 100. In example i, calculation of the mean of a vector x is done by using the function *mean(x)* in R, while in j the equivalent calculation is performed by dividing the sum of x by its length, using the R functions *sum(x)/length(x)*. Using method j is on average 6 times faster than using the function "mean" in i. The bar in j is also colored grey (compiled) as these functions give more direct access to their lower-level C equivalents (termed "primitives") while the function "*mean*" contains more overhead which slows it down, but increases its ease of use.

We immediately see that the examples using compiled code in C are approximately 8 times faster, on average. The reason for this is simple: the compiled function has already been processed by the compiler into a readily executable form (machine language), while for the pure R code, some processing is required even at run time to interpret the instructions at every iteration. Additionally, we can see that simply adding parentheses, which causes extra work for the interpreter, increases computation time in R (Fig. 1.3 a, c, e & g). The slow-down is non-trivial, with an average increase in execution time of 14.7% per added pair of parentheses on our test machine. Compiled languages, in contrast, are immune to this overhead (Fig. 1.3 b, d, f & h).

2 Optimizing R code: a bootstrap example

Now that we are a little bit familiar with profiling, and what slows down computation in R, let's continue with a more practical example. In the next sections we will be inspecting some methods with which we can speed-up a bootstrap operation similar to the one in the main text. We start with a program that is a typical example of "unpolished" code, and then continue to profile and optimize the code in a series of steps. In the later sections, we run our highly optimized code in parallel on multiple processors to obtain a 18.8 × speed-up (see the main document for more details).

2.1 Bootstrapping a moderately large biodiversity dataset

In the main document we gives examples using a large dataset of 750 million records, however to make this example accessible to readers with a less RAM at their disposal we have chosen to use a smaller dataset here. Imagine we have a moderately large biodiversity dataset, with 750 000 records, which may resemble something like species counts from N plots at S different sites (e.g. Hennekens & Schaminée, 2001). To create such a fake dataset in R, we could use the following code:

```
## OnlineRcode17.R

N<-7.5*10^2 #Number of records per site

S<-1000 # Number of sites
BioData<-data.frame(S=rpois(N*S,15),
                    site=as.factor(rep(1:S,N)))
```

Our goal is to calculate for each site 10 000 bootstrapped estimates of differences between site-specific species richness and the overall mean species richness

across all sites. Using *base* R, however, we have a problem, for although our dataset is not particularly large, the bootstrap routine in R (the function *boot*, in the *boot* package) can't handle its size. After first defining how many bootstrap resamples (10 000) we want:

R Example 2.1.

```
## OnlineRcode18.R

R<-10000 # number of bootstrap resamples

require(boot)

SiteMeans<-function(x,d,){
  tapply(x$S[d],x$site[d],mean)-mean(x)
}

BtResults<-boot(BioData,SiteMeans,R)
```

we get the following error[5].

```
Error: cannot allocate vector of size 55.9 Gb
```

This error occurs because within the boot function all re-samples are generated in one go. This ensures that random numbers will be independent even when *boot* is run in parallel[6], but causes problems when datasets become moderately large (as we reach the limits of the internal R function $sample.int(7.5 * 10^5, 7.5 * 10^5 * 10000, replace = TRUE)$ which is used "behind the scenes" in *boot*).

A way around this problem would be to define some function that will execute a standard amount, say 10 000 re-samples of the dataset and calculate our statistics of interest. The following code will conduct a bootstrap as required by our goal, albeit not quite optimally. Let's call this function $NaiveBoot$ and save it as a file called $NaiveBoot.R$.

[5]Note: depending on your system, and available memory you may get another error message than the one we got here using R 3.1.0.

[6]See the main document on why this is important (section *How to optimize: parallelization*).

R Example 2.2.

```
## OnlineRcode19.R

##Naive bootstrap function in R
NaiveBoot<-function(x,R){
  results<-NULL
        for(i in 1:R){
        index<-sample(seq_len(nrow(x)),replace=TRUE)
          results<-rbind(results,
          tapply(x$S[index],x$site[index],
          function(X) mean(X)-mean(x)))
        }
        return(results)
}

##Save the function to a source code file
dump("NaiveBoot",file="NaiveBoot.R")
```

Before we run the full bootstrap operation, let's see how fast our program runs by profiling it using a smaller problem. In essence a bootstrap problem has two aspects that will influence run time: 1) the size of the dataset that needs to be reshuffled and 2) the amount of resamples. So let's reduce the problem by conducting only 10% of the 10 000 resamples on only 10% of the dataset. We can now be confident that we have decreased the size of our problem considerably.

R Example 2.3.

```
## OnlineRcode20.R

## make a small subset of the data to work with
subBioData<-BioData[1:7.5e4,]

# 10% of the 10 000 resamples
subR<-1000
```

Next we start the profiling operation (again, we should not forget to switch on line-profiling in the *Rprof* function call).

R Example 2.4.

```
## OnlineRcode21.R

## reload our program so everything matches up exactly
source("NaiveBoot.R")

## Switch on R's profiler
Rprof(file="NaiveBoot.out",line.profiling =TRUE)

## set the random seed so we can compare results
## later.
set.seed(123)

## Run NaiveBoot on a subset of data 1000 times
ResultsNB<-NaiveBoot(subBioData,subR)

## stop profiling
Rprof(append=F)
```

Now that we have the R profiler output, we can find out where the bottlenecks are in our program.

```r
## OnlineRcode22.R

## Load Amdahl's profiler
require(aprof)

## make an object of the aprof class
NaiveBootAprof <- aprof("NaiveBoot.R","NaiveBoot.out")

## Plot the execution time per line
plot(NaiveBootAprof)
```

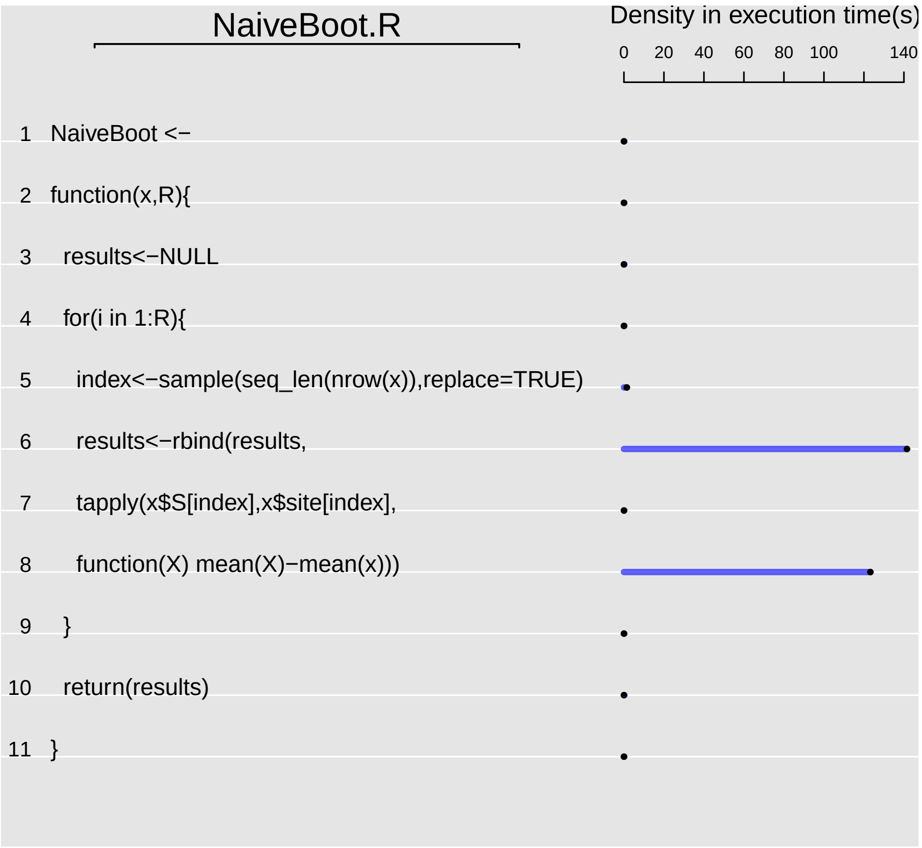

Figure 2.1: Execution-time density plot for the function *NaiveBoot*. Here we clearly see that the largest amount of time is spent in lines 6 and 8.

In this case it seems like it would be fruitful to look carefully at lines 6 and 8 (see Fig. 2.1). First, however, we should evaluate whether its worthwhile to attempt to optimize the code.

R Example 2.5.

```
## OnlineRcode23.R

summary(NaiveBootAprof)

## Largest attainable speed-up factor for the entire program
##
##         when 1 line is sped-up with factor (S):
##
##   Speed up factor (S) of a line
##             1     2     4     8     16    S -> Inf**
## Line*: 6 :  1.00  1.36  1.66  1.87  1.99  2.13
## Line*: 8 :  1.00  1.30  1.53  1.68  1.77  1.86
## Line*: 5 :  1.00  1.00  1.00  1.00  1.01  1.01
##
## Lowest attainable execution time for the entire program when
##
##              lines are sped-up with factor (S):
##
##   Speed up factor (S) of a line
##             1      2      4      8      16
## All lines   266.3  133.1   66.6   33.3   16.6
## Line*: 6 :  266.3  195.5  160.1  142.4  133.6
## Line*: 8 :  266.3  204.7  173.9  158.4  150.7
## Line*: 5 :  266.3  265.6  265.2  265.0  264.9
##
##     Total sampling time:  266.3  seconds
##  *  Expected improvement at current scaling
##  ** Asymtotic max. improvement at current scaling
```

The output from the function *summary* (in the row "All lines") basically confirms what we saw when eye-balling lines 6 and 8 in figure 2.1, that optimizing these lines will lead to meaningful speed-ups (at least theoretically[7]). We will run through the table output again quickly. We see that optimizing line 6 will give the greatest returns for our efforts (which can be expressed by the speed-up factor "S"). For instance if we figure out a way to make line 6 or line 8 execute 4 times faster the overall execution time of the program will speed-up between 53-66% (upper table) depending on whether we speed-up line 6 or 8. If we speed-up both the full program's completion is projected to drop to almost 66 seconds (bottom table, at S = 4; we can safely ignore line 5 in this example). Therefore, we should focus our efforts on lines 6 and 8 at this stage. As discussed in the main document, we can identify the usual suspects slowing down our operations: line 6 contains a reference to *rbind*, and here we are growing an object to store our results. This is highly inefficient. In line 8 we are repeatedly calculating the overal mean, this is completely unnecessary as we only need to do this once. Our next step should therefore be to pre-allocate space in the memory for our results and to calculate the overall mean only once. Our new bootstrap function $LessNaiveBoot$ then looks like this:

R Example 2.6.

```
## OnlineRcode24.R


## Less naive
LessNaiveBoot<-function(x,R){
  avg<-mean(x$S)

  results<-array(dim=c(R,nlevels(x$site)))
        for(i in 1:R){
        index<-sample(seq_len(nrow(x)),replace=TRUE)
          results[i,]<-tapply(x$S[index],x$site[index],
          function(X) mean(X)-avg)
        }
        return(results)
```

[7]However, there is something we should keep in mind when looking at the expected gains returned by the summary function in $aprof$: these expected gains are subject to the scaling of the problem. Scaling involves the question of how the complexity of the calculations change with the size of the problem. For instance, matrix operations will generally scale with the matrix dimensions ($N \times N$) by a factor of N^3, meaning larger matrices will require ever greater amounts of computation. This is important to keep in mind as it means that if you profile your program with a smaller example, due to scaling the actual benefits when you conduct the full-scale problem may be far greater - and vice versa - if you profile your code with a complex problem, you should not be surprised that the gain of any optimization is far less on simpler versions.

```
}

# Save the function to a source code file
dump("LessNaiveBoot",file="LessNaiveBoot.R")
```

Next, let's see what we have achieved by timing the run of our updated code.

R Example 2.7.

```
## OnlineRcode25.R

## reload our saved file
source("LessNaiveBoot.R")

set.seed(123) # set seed for results comparison

# Profile the program
Rprof(file="LessNaiveBoot.out",line.profiling =TRUE)
ResultsLNB<-LessNaiveBoot(subBioData,subR)
Rprof(append=F)

# make an aprof object
LessNaiveBootAprof <- aprof("LessNaiveBoot.R","LessNaiveBoot.out")

# Summarize the gains
summary(LessNaiveBootAprof)
```

R Example 2.8.

```
## Largest attainable speed-up factor for the entire program
##
##         when 1 line is sped-up with factor (S):
##
##   Speed up factor (S) of a line
##              1     2     4     8     16    S -> Inf**
## Line*: 7 :  1.00  1.49  1.97  2.35  2.61  2.92
## Line*: 8 :  1.00  1.17  1.29  1.35  1.39  1.42
## Line*: 6 :  1.00  1.02  1.03  1.04  1.04  1.04
## Line*: 4 :  1.00  1.00  1.00  1.00  1.00  1.00
##
```

```
## Lowest attainable execution time for the entire program when
##
##              lines are sped-up with factor (S):
##
##   Speed up factor (S) of a line
##             1      2      4      8      16
## All lines   29.02  14.51   7.26   3.63   1.81
## Line*: 7 :  29.02  19.48  14.71  12.33  11.13
## Line*: 8 :  29.02  24.70  22.54  21.46  20.92
## Line*: 6 :  29.02  28.42  28.12  27.97  27.89
## Line*: 4 :  29.02  29.01  29.00  29.00  29.00
##
##     Total sampling time:  29.02  seconds
##  *  Expected improvement at current scaling
##  ** Asymtotic max. improvement at current scaling
```

We see that pre-allocation has given us a clear improvement! Starting off with an execution time of about 266 seconds, we were able to shave off 237 seconds (an improvement of roughly 850%) from the execution time by removing some obvious causes of slowdown in our code. Before we continue we should test whether our new function gives the same results as our previous version.

```
## OnlineRcode27.R

## test if results are equal
all.equal(as.numeric(ResultsNB),as.numeric(ResultsLNB))
## TRUE
```

This is indeed the case. We have successfully optimized the function. However, were these changes truly the best candidates for a speed-up? Or, is there more room for improvement? Let's take a detailed look at our improved function to find out.

```
## OnlineRcode28.R

## Plot the execution time per line
plot(LessNaiveBootAprof)
```

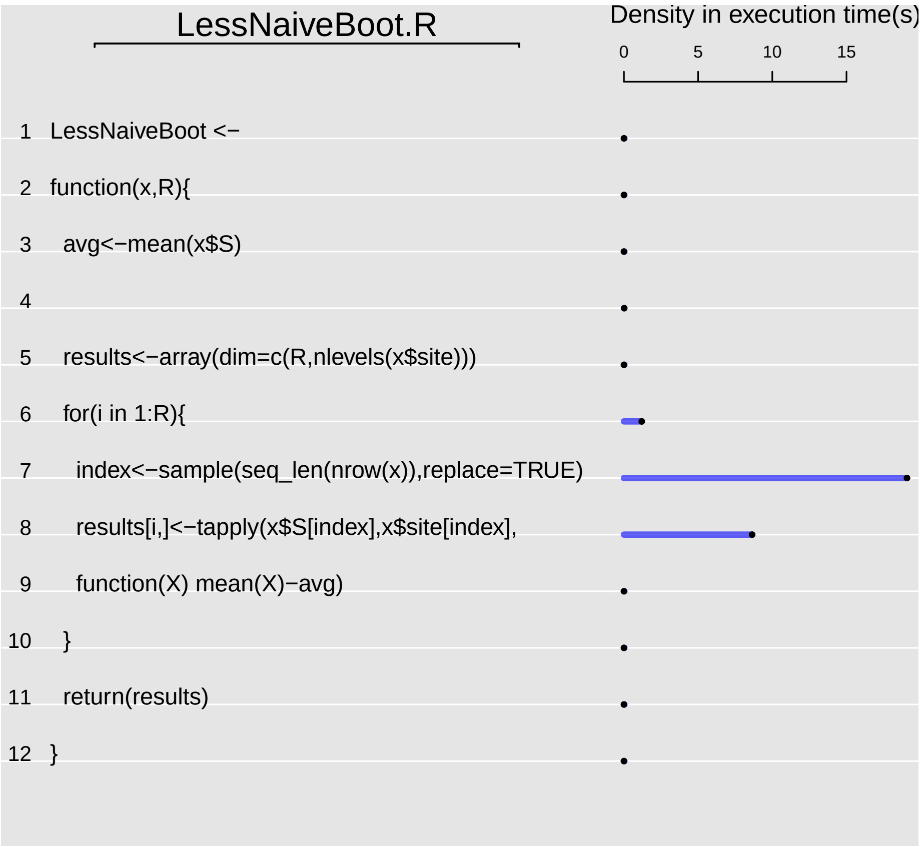

Figure 2.2: Execution-time density plot for the function *LessNaiveBoot*. Here we clearly see that the largest amount of time is again spent in line 7. However, on which function in line 7 should we focus on now?

Eye-balling figure 2.2, we see that most time is spent in line 7, and the table returned by *aprof* above shows that it should be worthwhile to further optimize the functions in line 7. However, we now also see that it is no longer quite as obvious where we should focus our optimization efforts in line 7, because there are several functions active in that line (see figure 2.2).

To help identify which function is slowing down our calculations in line 7, we will next take a targeted look at the functions in line 7. To achieve this we can use *aprof's* function *targetedSummary* and take a more detailed look at the functions being called in this line.

The function *targetedSummary* returns the execution time spent within each function, expressed both in the amount of calls in the record (samples) returned by the R profiler and in run time. Please, note that when a function within another function is 'called' by the R profiler, both functions get a hit ('Calls'). In this case, therefore, the outermost function *tapply* gets the most 'Calls'. Let's run *targetedSummary* and print the first ten elements, which are the ten most-frequently called functions:

R Example 2.9.

```
## OnlineRcode29.R

## check if the target line is also 7 on your system
head(targetedSummary(target=7,LessNaiveBootAprof),10)

##         Function Calls  Time
## 1         tapply   953 19.06
## 2         lapply   771 15.42
## 3             L8   443  8.86
## 4            FUN   442  8.84
## 5           mean   402  8.04
## 6          split   297  5.94
## 7  split.default   296  5.92
## 8      as.factor   161  3.22
## 9   mean.default   129  2.58
## 10        unlist    77  1.54
```

Using *targetedSummary* we can clearly see which functions are taking the most time within line 7. The vast majority of time is spent within such R functions as *tapply*, *lapply*, *split*, *split.default*, *mean*, *mean.default* and *as.factor*. However, as we can see in figure 2.2, we never used the functions *lapply*, *split.default* or *as.factor* in line 7. Upon investigation we see that these functions show up in the output from *targetedSummary* because they are called within *tapply* (type *page*(*tapply*) to use R's pager to confirm this in the code of *tapply*). The function *tapply* is their parent function. We can get this information directly from *targetedSummary* by setting the "findParent" option to "TRUE":

R Example 2.10.

```
## OnlineRcode30.R

## check if the target line is also 7 on your system
head(targetedSummary(target=7,LessNaiveBootAprof,findParent=TRUE),10)

##          Function        Parent Calls  Time
## 1          tapply            L7   953 19.06
## 2          lapply        tapply   771 15.42
## 3              L8           FUN   443  8.86
## 4             FUN        lapply   442  8.84
## 5            mean            L8   402  8.04
## 6           split        lapply   297  5.94
## 7   split.default         split   296  5.92
## 8       as.factor split.default   161  3.22
## 9    mean.default          mean   129  2.58
## 10         unlist        tapply    77  1.54
```

This shows the nesting of all functions within their parent functions. We see that *tapply* is called in line 7 (L7), *lapply* within *tapply*, line 8 (L8) is called from within our function FUN (this is where we set function(X) in *tapply*, which in turn calls the function mean and so on). We now can conclude that most time is spent in the function *tapply* which calls *lapply*. And looking further we see that within *lapply* a large amount of time is spent executing the function *mean*. Interestingly, we also see that *mean* is the parent function of $mean.default$ (and $mean.default$ is therefore a lower-level function of *mean*). As we have stated in 1.4.1, higher level functions are easy to use (they do auto-formatting of data, error checking, and other useful checks). However, these checks can be seen as overhead that slow down computation, and we can avoid some overhead by using the lower-level version of *mean*: $mean.default$. Lets adapt your previous code and call $mean.default$ directly:

R Example 2.11.

```
## OnlineRcode31.R


## Less naive
fastBoot<-function(x,R){
  avg<-mean(x$S)

  results<-array(dim=c(R,nlevels(x$site)))
        for(i in 1:R){
        index<-sample(seq_len(nrow(x)),replace=TRUE)
```

```
            results[i,]<-tapply(x$S[index],x$site[index],
            function(X) mean.default(X)-avg)
        }
        return(results)
}

# Save the function to a source code file
dump("fastBoot",file="fastBoot.R")
```

Next lets find out if our code is any faster and, importantly, see if your results are still the same.

R Example 2.12.

```
## OnlineRcode32.R

## reload our saved file
source("fastBoot.R")

set.seed(123) # set seed for results comparison

# Profile the program
Rprof(file="fastBoot.out",line.profiling =TRUE)
ResultsFB<-fastBoot(subBioData,subR)
Rprof(append=F)

## test if results are equal
all.equal(as.numeric(ResultsNB),as.numeric(ResultsFB))
## TRUE

# make an aprof object
fastBootAprof <- aprof("fastBoot.R","fastBoot.out")

# Summarize the gains
summary(fastBootAprof)
```

R Example 2.13.

```
## Largest attainable speed-up factor for the entire program
##
##         when 1 line is sped-up with factor (S):
##
##   Speed up factor (S) of a line
##             1     2     4     8     16    S -> Inf**
## Line*: 7 :  1.00  1.62  2.35  3.03  3.55  4.28
## Line*: 8 :  1.00  1.07  1.10  1.12  1.13  1.14
## Line*: 6 :  1.00  1.05  1.08  1.10  1.10  1.11
## Line*: 4 :  1.00  1.00  1.00  1.00  1.00  1.00
##
## Lowest attainable execution time for the entire program when
##
##              lines are sped-up with factor (S):
##
##   Speed up factor (S) of a line
##             1       2       4       8       16
## All lines   15.660   7.830   3.915   1.958   0.979
## Line*: 7 :  15.660   9.660   6.660   5.160   4.410
## Line*: 8 :  15.660  14.670  14.175  13.928  13.804
## Line*: 6 :  15.660  14.870  14.475  14.277  14.179
## Line*: 4 :  15.660  15.650  15.645  15.643  15.641
##
##     Total sampling time:  15.66  seconds
##  *  Expected improvement at current scaling
##  ** Asymtotic max. improvement at current scaling
```

Indeed, by using the lower-level version of *mean* we eliminated much overhead. We are able to cut the execution time by roughly 50% which is good, and at the same time less good. We should realize that we are now using a lower-level function, which tends to be much less robust, and must now be more cautious. For instance, *mean* is versatile and can be used on e.g. dates:

R Example 2.14.

```
## OnlineRcode34.R

dates<-as.Date(c("02/27/99", "02/27/91", "01/14/95"), "%m/%d/%y")
mean(dates)

## [1] "1995-02-12"
```

While $mean.default$ fails:

R Example 2.15.

```
## OnlineRcode35.R

mean.default(dates)

## Warning:  argument is not numeric or logical:  returning NA

## [1] NA
```

This illustrates that there are dangers in optimizing code: optimized code loses generality. A rule seems to be that the more optimized a function becomes it tends to work on an ever narrowing set of conditions. This is important to realize as we optimize further, that the number of problems for which we can use our increasingly faster bootstrap code will now start to decrease.

We can continue with a final optimization step. The table above shows us that line 7 is still the slowest line in our program, and that optimization of this line will likely yield the best returns. Therefore, let's investigate which functions within line 7 are taking up the most time.

R Example 2.16.

```
## OnlineRcode36.R

## check if the target line is also 7 on your system
head(targetedSummary(target=7,fastBootAprof,findParent=TRUE),10)

##         Function        Parent Calls  Time
## 1         tapply            L7   599 11.98
## 2         lapply        tapply   398  7.96
## 3          split        lapply   258  5.16
## 4  split.default         split   258  5.16
## 5      as.factor split.default   125  2.50
## 6             L8           FUN   114  2.28
## 7            FUN        lapply   107  2.14
## 8         unlist        tapply    99  1.98
## 9   mean.default            L8    86  1.72
## 10          sort     as.factor    49  0.98
```

We again see the nesting of all functions within their parent functions: *tapply* is called in line 7 (L7), *lapply* within *tapply*, *split* within *lapply* and *split.default* within *split*. These are now the functions (within line 7) that take the most time. Upon investigation we see that they are concerned with ordering our data into groups, from which we calculate site means. Clearly, it is the grouping of our pseudo-ecological dataset that is now causing the greatest slowdowns.

We are using a "data.frame" to store the data, and in R "data.frames" are special forms of lists, that are very useful as they store multiple types of data (e.g. integers, characters, factors) within one single $data.frame$. However, this usefulness seems to come at a cost: before the site means can be calculated the information in the data has to be extracted out of the *data.frame* and then ordered per site. This is slowing things down. To speed-up execution time here, we could abandon the $data.frame$ class and adopt a N × S matrix, where each column contains the data for a specific site. This will be quicker as we have essentially extracted and ordered the relevant information ourselves prior to starting the bootstrap. We can then calculate means with the highly optimized function $colMeans$ (see the main document for an example). This is a form of "memoization", where we remove the need to repeatedly order and extract data. This would likely work in this simple example, but not all datasets will be easily converted to matrices (a matrix can only contain one type of data). Therefore, if we did this we will make our program far less general.

We have already achieved an impressive speed-up (from 266 seconds to 16) and therefore we can be confident that we have a sufficiently efficient program in serial. In the next section (3) we start our final speed-up exercise for this example; running our code in parallel. One thing to note, as pointed out by an anonymous reviewer of this document, is that we could replace our function $fastBoot$ with a version that contains no loops at all:

R Example 2.17.

```
## OnlineRcode37.R

NoLoopsfastBoot <- function(x, R) {
  avg<-mean(x$S)
  t(replicate(R, {
         index <- sample(seq_len(nrow(x)), replace=TRUE)
        tapply(x$S[index], x$site[index],
                function(X) mean.default(X)-avg)
    }))
}
```

This version is the equivalent of $fastBoot$, it's not particularly faster or slower.

R Example 2.18.

```
## OnlineRcode39.R

set.seed(123)
system.time(noloops<-NoLoopsfastBoot(x=subBioData,R=100))
```

```
##    user  system elapsed
##   1.712   0.048   1.765

set.seed(123)
system.time(loop<-fastBoot(x=subBioData,R=100))

##    user  system elapsed
##   1.620   0.000   1.623

all.equal(as.numeric(noloops),as.numeric(loop))

## [1] TRUE
```

However, it has as explicit benefit that you don't have to worry about preallocation: the R functions used already do this efficiently behind the scenes. This can be an efficient way of programming, though some may find the code harder to read and less clear than a for loop.

3 Parallel computing

Parallel computing divides calculations into smaller problems and solves these simultaneously using multiple computing elements (hereafter "workers"). Once prominently used only in super-computing (ref [5] in the main text), it is today commonplace as most desktop computers and laptops have CPUs with multiple "cores". Parallel computing has also become relatively straightforward to implement, but is not implemented by default in R (Knaus *et al.*, 2009). In the next sections we provide an introduction to parallel programming. The benefits of parallelism can be great, however, before implementing parallel-programming or investing in hardware, a handful of basic rules are worth reviewing.

1. A common misconception is that when more processes are run in parallel the faster the problem will be solved (ref [5] in the main text). This misconception is easily seen by applying Amdahl's Law (Fig. 3 in the maintext), which shows that the possible speed-up is asymptotically related to the amount of parallel processes. Even when a large fraction of the code can be run in parallel the maximum speed-up is limited (and each additional worker offers diminishing returns). Applying both profiling and Amdahl's law can identify the optimal amount of parallel processes beforehand. We provide this functionality in the package *aprof.*

2. Parallel computation incurs overhead. Initializing a parallel computation takes some time, including communication between devices or across nodes to copy code and data. When individual jobs are not reasonably intensive compared to the time it takes to initiate the parallelization (i.e. the ratio of computation to communication is low), repeatedly starting parallel threads will deteriorate overall performance. This sometimes means that one should not use all workers available, especially for smaller repetitive tasks.

3. Avoid over-parallelization, and do not assign more workers than available. If the machine is used for additional tasks besides calculation, leave some resources (i.e. physical cores) for other processes. If not, the system may become unstable.

4. In most computing devices, memory (RAM) is shared among parallel processes (Schmidberger *et al.*, 2009). Therefore, each of N processes running in parallel will only receive 1/N of the available memory. If memory is not available within a thread, threads will automatically attempt to "borrow" memory from another process. While parallel workers wait for memory to become available, overhead is drastically increased to the point that execution of the problem in serial may be faster. One can use R functions such as *object.size* to determine memory usage in an algorithm.

5. Independence of random number sequences must be ensured for valid scientific results (e.g. L'Ecuyer, 2012). A crucial consideration when conducting operations that rely on random numbers is that all parallel calculations

have unique sequences that will not overlap and can be reproduced. Specific techniques are available that ensure independence of random number sequences within R (e.g. L'Ecuyer, 1999, section 3.4)).

3.1 Parallel computing in R

Programming in parallel in short, involves starting a main process, often called the parent process, which (1) initializes the calculations, (2) divides them and sends these as "jobs" to "child" processes and (3) waits for the "child" processes to return results once they have completed.

R has multiple packages to execute parallel code. These packages include *foreach*, *multicore* and *snow* to name a few (Weston & Computing, 2013; Urbanek, 2011; Tierney *et al.*, 2013), and some packages for parallel calculations on a graphics processing unit or "GPU" (e.g. gputools Buckner *et al.*, 2013). In this tutorial we will use the package *parallel* which is shipped with the core version of R (since R version 2.14.0). In this document we will focus on parallel computing on a single machine (i.e. a laptop) with two or more cores (these techniques will also work on servers with many more cores). We do not focus on parallel computing via clusters or using several computers connected via ethernet, though the reader should be aware that these operations are readily available and easy to implement in R (see e.g. the *snow* package Tierney *et al.*, 2013).

Take special care when using programs (e.g. a custom BLAS library, pqR) that automatically make use of parallel operations, as these can easily cause the proliferation of jobs far beyond the amount of workers if used within parallel algorithms.

3.2 Parallel calculations using forking (Unix systems including Mac)

One of the easiest ways of conducting parallel computations in R is through forking. However, this can only be done on Unix machines (essentially anything but Windows). In the next section (3.5) we give an example for Windows. Windows users should not skip this part, as we address some key issues for parallel computing. They may, however, skip running most of the code examples. More important considerations for parallel computing are given in the main document.

3.3 Number of workers

One thing that needs to be ensured in parallel computing is that we avoid over-parallelization. We should not assign more jobs then there are "workers", or we will surely paralyze our code. Therefore, it is important to find out how many physical workers are available (for details see the main document). In R we can do this using the following code:

R Example 3.1.

```
## OnlineRcode40.R

require(parallel)

## Loading required package:  parallel

ncores<-detectCores()
print(ncores)

## [1] 8
```

We see that the computer on which this document was made has 8 cores. However, we don't want to use all the cores when running this code, as we are using our machine for more than just computation (e.g. writing this document). This is why we adjust the output of *detectCores* below, to ensure we don't use all the resources (physical cores and dual-threaded capabilities) for computation. We do this to ensure stability of the system by leaving enough computing power for the other things we need to do. If you are using a dedicated machine (e.g. on a server) you can of course assign all the workers available to you (though even then it may be smart to leave some idle).

R Example 3.2.

```
## OnlineRcode41.R

require(parallel)
ncores<-detectCores()/2
print(ncores)

## [1] 4
```

3.4 Random numbers

Another thing we need to consider, before we start, is that bootstrapping relies on random number generation. We must ensure that sequences of random numbers generated, within each child process, are truly independent. To do so, we need each parallel stream to have a separate random seed. However, not all seeds are equally good as pseudo Random Number Generators (RNG) - as they are called - are typically also periodic, meaning that sequences will eventually repeat. If seeds are not chosen well, streams within each parallel calculation may overlap and will therefore no longer be independent. Lastly, we would also

like our random numbers to be repeatable when we keep the same seed. To make sure all this takes place we change the random number generator in use to the L'Ecuyer RNG (L'Ecuyer, 1999) with $RNGkind("L'Ecuyer - CMRG")$, which is a random number generator specifically designed for use in parallel computations. This ensures that we will have independent and reproducible random numbers.

R Example 3.3.

```
## OnlineRcode42.R

## set RNG to "L'Ecuyer-CMRG"
RNGkind("L'Ecuyer-CMRG")
```

Now that we know how many workers we have available and we know which RNG to use, let's build our first parallel algorithm to test if we can reproduce random numbers. We will generate random values, in parallel, from a normal distribution using *rnorm*. To do this, we will utilize the function *mcparallel* in R. You will see that its usage is remarkably simple. Note that when you build parallel algorithms you should avoid using any GUI elements (e.g. graph plotting, printing) as they may lead to problems. The code for Windows users is given in 3.5.

R Example 3.4.

```
## OnlineRcode43.R

require(parallel)

## number of workers
ncores<-2

## make an object to save the output from each child process
children<-vector("list", ncores)

# change the random number generator
RNGkind("L'Ecuyer-CMRG")

## set an initial seed
set.seed(20130808)

## The following will make runs from mcparallel reproducible
mc.reset.stream()
```

```
## initialize each child process
for(i in 1:ncores){
        children[[i]]<-mcparallel(rnorm(4))
}

# collect results
randomnumbers<-parallel::mccollect(children)
```

Let's see if we can reproduce these results by re-running the code with the original seed:

R Example 3.5.

```
## OnlineRcode44.R

## reset an initial seed
set.seed(20130808)
mc.reset.stream()

## re-initialize each child process
    for(i in 1:ncores){
        children[[i]]<-mcparallel(rnorm(4))
        }

## collect results
randomnumbers2<-parallel::mccollect(children)
```

Now lets see if the random numbers are identical:

```
## OnlineRcode45.R

print(randomnumbers)

## $`31758`
## [1] -0.6172  0.1962  0.6680 -1.0425
##
## $`31759`
## [1]  0.4541 -0.1770  0.3327 -0.4369

print(randomnumbers2)
```

```
## $`31760`
## [1] -0.6172  0.1962  0.6680 -1.0425
##
## $`31761`
## [1]  0.4541 -0.1770  0.3327 -0.4369
```

Success! We now know how to conduct a parallel computation via forking, and ensure we have reproducible results. Let's apply this to our bootstrap problem and see what our gains are.

3.4.1 The parallel bootstrapping algorithm via forking

Building a parallel algorithm to conduct our bootstrap will be relatively easy. However, we will need to find a way to split our calculations. In this case the most logical place to split the calculations into equal parts would be to divide the number of re-samples among workers. The following is one way to do this:

R Example 3.6.

```
## OnlineRcode46.R

subR<-1000

## Set the number of workers we will use
ncores<-3

## Split the jobs
splitR<-table(cut(1:subR,ncores,labels=F))
print(splitR)

##
##   1   2   3
## 334 333 333
```

As can be seen, we have now produced an almost equal split among 3 workers. Next, let's start our parallel bootstrap - by sending our (almost) equal job splits to the different children. As each of the children are independent processes we opt to not use a profiler to time the execution of this process. Instead we record the start and end times. The equivalent of the below code for Windows users is given in section 3.5. Note that the Windows code is slightly simplier, and also works well on Unix systems.

R Example 3.7.

```r
## OnlineRcode47.R

## load the parallel package
require(parallel)

## save the start time
tp0 <- structure(.Internal(Sys.time()))

## initialize a list where we can store the id of each child
children<-vector("list", ncores)

## send the division of work in splitR to each of the cores
for(i in 1:ncores){
        children[[i]] <- mcparallel(fastBoot(subBioData,
                                    splitR[i]))
}

## Wait for the child processes named in "children" to finish
results <- mccollect(children)

## Record end time
tp1 <- structure(.Internal(Sys.time()))

## Calculate execution time
tp <- tp1-tp0
```

Which gives us:

```
print(tp)
[1]  5.529923
```

That's it. We completed a parallel execution of the bootstrap and the final execution time of our program was about 5.5 seconds. This means that we gained a relative speed-up of 280% above our fastest serial code!

3.5 Alternative parallel bootstrapping algorithm (including Windows)

Adapting the above guidelines for Windows is straight forward.

R Example 3.8.

```
## OnlineRcode48.R

require(parallel)

## number of workers
ncores<-2

## set an original seed
set.seed(20130808)
mclapply(rep(4,ncores),rnorm)

## [[1]]
## [1] -0.6172  0.1962  0.6680 -1.0425
##
## [[2]]
## [1]  0.4541 -0.1770  0.3327 -0.4369

## reset the original seed
set.seed(20130808)
mclapply(rep(4,ncores),rnorm)

## [[1]]
## [1] -0.6172  0.1962  0.6680 -1.0425
##
## [[2]]
## [1]  0.4541 -0.1770  0.3327 -0.4369
```

Again, as in section 3.4, we succeeded in generating repeatable random numbers that are independent with each parallel process. Next let's finish the parallel bootstrap for Windows. The following deploys the same parallel bootstrap as we did before:

R Example 3.9.

```
## OnlineRcode49.R

## load the parallel package
require(parallel)

##initialize the problem
subR<-1000
```

Program	NaiveBoot	LessNaiveBoot	fastBoot	Parallel
Execution time	1 hour and 3 minutes	38 minutes	12 minutes	3.35 minutes

Table 3.1: The end gains. Required execution time for each of the program versions to conduct 10 000 bootstrap resamples on a dataset of 750 000 records.

```
## Set the number of workers we will use
ncores<-3

## Split the jobs
splitR<-table(cut(1:subR,ncores,labels=F))

## save the start time
tp0 <- structure(.Internal(Sys.time()))

## send the division of work in splitR to each of the cores
results <- mclapply(splitR,function(X)
                    fastBoot(subBioData,X))

## Record end time
tp1 <- structure(.Internal(Sys.time()))

## Calculate execution time
tp <- tp1-tp0
```

3.6 The end gains

The final question that needs to be answered is, in the end, how much did we gain when we conduct the full problem? That being 10 000 bootstrap resamples on the full dataset with 750 000 records. We have summarized the full run time of each program in table 3.1, here we see that in the end we have sped-up execution of our problem by a factor of ≈ 18.8 (from 1 hour 3 minutes to 3.35 minutes). This is a significant improvement.

3.7 Closing remarks

Parallel computation via cloud computing and computing clusters are also becoming common at many institutions (e.g. Harvard University's Odyssey Cluster), increasing the value to learn parallelization techniques for the future. Beyond this, it is now possible for scientists to implement massively parallelized

code on graphics cards (or graphics processing units, GPUs), which have hundreds to thousands of processors, and are relatively cheap (starting at only a few hundred dollars). New developments, such as the CUDA platform and programming model (Nvidia, 2007), which enables execution of C, C++ and Fortran code on GPUs, are freely available to scientists. Such prospects show that we are only starting to scrape the surface of computational feasibility.

Most scientists use desktop or laptop computers (Hannay *et al.*, 2009), and in recent years most of these computers have become capable of parallel computing (Wilson, 1995). Coincidentally, many computationally intensive problems in the biological sciences rely on so-called 'embarrassingly parallel computations' (Grama, 2003), where a very large fraction of all calculations can be completed in parallel. Consequently, parallel computing is potentially the most useful technique highlighted in this document. Implementing code in parallel is also one of the simplest ways of speeding up an analysis. We hope that we have shown here that there is no reason not to take advantage of all the computing power available at our fingertips today.

4 Bootstrapping large data in R

R loads and stores objects in memory, and when an object is comparable in size to the size of the (virtual) memory, R slows to a crawl. Worse, when a dataset is larger than the available memory, R will fail to load it. When this happens, one can either buy more RAM, or use a more memory efficient algorithm (see the section on "large data" in the main document for more details). In this section we give an example on how to implement the previously optimized bootstrap code in a memory efficient way using a package designed to store objects on hard drives rather than RAM: package *ff* (ref 23 in the main text). This package only "maps" to the data stored on a disk, and thus it allows working with datasets that do not fit in memory.

We chose to highlight this package as it is relatively easy to use. For instance, the data structures it provides behave (almost) like normal R objects (as if they were stored in RAM), making it more intuitive for R users. In combination with the package *ffbase* (de Jonge *et al.*, 2014), users have - to a large extent - the functionality from the R's *base* package (almost all basic functions will work):

1. support for multiple data types 'double', 'logical', 'raw' and 'integer' (and others)
2. basic operators as $(+, -, *, /, <, >, ==$ and more)
3. basic mathematical functions (*sqrt, log10, log2, log1p, exp, cos, sin, cumsum*, etc)
4. most data manipulation tools (*subset, with, within, table, tabulate, merge, ffdfdply* and more)
5. and summary statistics (*sum, min, max, range, quantile, hist*)

The package also works well with other "big data" packages such as *bigglm* and *ff* files (on the disk) can be shared by multiple ff R objects (in the RAM) making it possible to exploit parallelism.

We will start our short example by loading data. As an example we will write our previously created dataset to the hard drive, and then load it as an "*ff* object". This illustrates the steps required to "load" a large dataset into R such as a "flatfile". Before we start, however, lets see how large our dataset is:

R Example 4.1.

```
## OnlineRcode51.R

## Biodata size in MB
object.size(BioData)[1]/1048600

## [1] 5.776
```

We see that BioData is pretty small, only about 6 mb, but it will suite our purposes for illustration. Next let's write it to the disk and create a *ff* object (which creates an associated *ff* file on the disk).

R Example 4.2.

```
## OnlineRcode52.R

## Write the data to the disk
write.table(BioData, file = "biodata.txt", sep=";", row.names=FALSE)

## load packages and create a ff object.
require(ff)
require(ffbase)
ffBioData <- read.table.ffdf(file="biodata.txt",
                             sep=";", header=TRUE,
                             colClasses=c("numeric","factor"))
```

Here we use *read.table.ffdf* to read the semi-colon seperated file from the disk and create a *ff* object. The function basically reads in the data sequentially, in chunks, and creates a *ff* file (on the disk) which it then links to the R object *ffBiodata* (in the RAM). The creation of a *ff* object may take a long time if your data is really big. Note that we specify the colClasses to ensure the process of reading and creating the *ff* object and file goes quicker and smoother. A *ff* object can also be saved for later use with the *ffdf()* function, so you don't have to do this every time. Next, let's see how much smaller the *ff* object is in R's memory:

R Example 4.3.

```
## OnlineRcode53.R

## Biodata size in MB
object.size(ffBioData)[1]/1048600

## [1] 0.06248
```

It is considerably smaller, but notice that at the same time it ”acts” exactly the same as our object *BioData* (which is loaded in the memory) in that we can use many of the standard R functions normally.

R Example 4.4.

```
## OnlineRcode54.R

dim(ffBioData)

## [1] 750000      2

nlevels(ffBioData$site)

## [1] 1000

max(ffBioData$S)

## [1] 37

quantile(ffBioData$S)

##   0%  25%  50%  75% 100%
##    0   12   15   18   37
```

The only difference is that in these cases, the data are not read from memory, but sequentially in blocks from the disk. One drawback of this method is that it is much slower: conducting operations on objects stored in the memory can be an order of magnitude faster.

R Example 4.5.

```
## OnlineRcode55.R

system.time(replicate(100,mean(BioData$S)))

##    user  system elapsed
##   0.088   0.000   0.088

system.time(replicate(100,mean(ffBioData$S)))

##    user  system elapsed
##   0.896   0.184   1.086
```

With this in mind, let's continue to the actual implementation of the bootstrap code. In this example we took care to use common functions from base R that should be familiar to most. You can go ahead and run this function, but note that it takes some time to complete.

R Example 4.6.

```
## OnlineRcode56.R


## Less naive
ffBoot<-function(x,R,N=N){
  avg<-mean(x$S)
  results<-array(dim=c(R,nlevels(x$site)))

  for(i in 1:R){
  results[i,]<-sapply(levels(x$site),function(X)
  mean(x$S[ffBiodata$site==X][sample(1:N,replace=TRUE)])-avg)
   }

  return(results)
}

# Save the function to a source code file
dump("ffBoot",file="ffBoot.R")
```

The above example code implements a simple bootstrap algorithm, however it is possible to implement more complex versions where the user has much finer control over the operation of reading the data sequentially from disk. For instance, using the function *"ffdfdply"* (instead of *sapply*) we can specify exactly how much data can be extracted in one chunk and loaded in RAM (using the option BATCHBYTES). This could allow one to optimize the function depending on your machine and data. We refer the reader to the *ff* and *ffbase* package manuals for greater detail and more examples if more control is needed. However, as many base functions will already work on *ff* objects, you now already have the basic tools to conduct the most common operations in R with this short tutorial.

5 Extending R: High performance with compiled code

Sometimes the only way to make your code run faster is to rewrite it in a lower-level language. In fact many high-level languages, like R, have modules written in C or Fortran for greater speed (e.g., R's vectorized functions). Programs written in lower-level languages sometimes require more development time and tend to be more complex to code and debug, but when performance bottlenecks are caused by language limitations (Fig. 1.3), refactoring may be the only option. In the final sections of this document, we will show you how to make C do the heavy lifting by extending R with C. This can be a complex operation for those not too familiar with programming in C. However, even those with little programming experience in C should be able to follow the simple examples outlined here. We do, however, advise you to expand your experience with C a bit before trying this on your own examples.

In this section we provide what is hopefully a user-friendly guide to writing R-extensions in C [8]. Before we start with another ecological example, we will run through the setup of the system and extend the simple example we used in section 1.3 to C. Readers should take care because calling compiled code from R can be rough and small errors in your C code may cause R to crash (e.g. segfaults - or memory access violations - are common). So, do this at your own risk, and make sure to save crucial analyses and data before you start. We refer to the "Writing R Extensions" manual for detailed information [9]. All the examples given below were tested on Windows, Mac and Linux machines.

5.1 Getting started with extending R

5.1.1 Getting started under Windows

Unfortunately Windows is not the most friendly environment for extending your R code with C, and you will have to take a few extra steps to get things done compared with your *UNIX based brethren. Luckily it is not difficult, as the good people developing R have bundled all the tools you need in one zip-file "Rtools", which you will need to download [10] and install [11]. This installation contains all the tools you need to build packages and compile C code for use in R. Once you have downloaded and installed the *Rtools*, you will need to take the following steps to change your environment variables. More detailed help

[8] At the end of this tutorial some readers may wish to try Rccp (Eddelbuettel & François, 2011), which is an R-package that attempts to ease the integration of R and C. As we show, *Rccp* is in no way required to write your own extensions, but for those who feel uneasy with writing C extensions or require easy transfer of data between R and C, the *Rccp* package will provide help.

[9] `http://stuff.mit.edu/afs/sipb/project/r-project/arch/i386_rhel3/lib/R/doc/manual/R-exts.html`

[10] `http://cran.r-project.org/bin/windows/Rtools/`

[11] the documentation for these tools can be found on this webpage `http://cran.r-project.org/doc/manuals/R-admin.html#The-Windows-toolset`

can be found in the R FAQ [12].

1. Change your PATH variables properly. To do this in the newer versions of Windows (e.g. Windows 7) go to the 'Control Panel', then click on 'User Accounts' and select 'Change my environmental variables' in the left panel [13].

2. Now we create a new environment variable with 'Variable name' PATH. And set as 'Variable value' the following paths '...\Rtools\bin;...\Rtools\gcc-4.6.3\bin;...\R-3.1.0\bin', where ... refers to the directories on your machine where *Rtools* and *R* are located, respectively. These paths correspond to the versions of *Rtools* (31) and *R* (3.1.0) on our Windows test machine.

3. Restart your computer.

5.2 Writing C code

Providing a comprehensive guide on coding in C is far beyond the scope of this tutorial. Luckily there are many online resources on C and C++ freely available. If you are a beginner at C, these guides will help you write simple C and C++ code within only a few hours of self study. This is fortunate, because, as we will show below, often only very simple programs are needed to speed-up code considerably. In this tutorial, we will give some basic pointers that should help to get you started with C code meant for use in R. We will start, step by step, by rewriting a simplified version of the R code in section 1.3. We use this code as a means to illustrate the steps you need to take to "load a dynamic library" to call C functions from R. It is not an example of a typical program that could benefit from calling C from R (in contrast to the program in section 6).

The code in R looks like this:

R Example 5.1.

```
## OnlineRcode57.R

SimpleR<-function(N){
  answer<-numeric(N)
  for (i in 1:N) { answer[i]<-(N/(1+N))}
  return(answer)
}
```

[12] http://cran.r-project.org/bin/windows/base/rw-FAQ.html#How-do-I-set-environment-variables_003f

[13] For other versions of Windows see http://cran.r-project.org/bin/windows/base/rw-FAQ.html#How-do-I-set-environment-variables_003f

The same function in C looks like this:

R Example 5.2.

```
## OnlineRcode58.R

#include <R.h>
#include <Rmath.h>

/* Rewrite of  InterpreterQuirks.R */
void SimpleC(int *nc, double *dnc, double *answerc) {

  int n = nc[0];
  double dn = dnc[0];
  int i;

  for (i=0; i<n; i++){
    answerc[i] = (dn/(1+dn));
  }
}
```

Before we can time the execution of the program in C we need to compile it, load it into R and create a wrapper function. However, before we do so, perhaps we should provide a very quick run through of the meaning of the different code elements in C, compared to how we code in R. Those familiar with C who don't feel the need for a short refresher, can skip the next part.

5.2.1 C coding in a nutshell

C code is surely different from R, but even with no C experience one should be able to comprehend exactly what is going on. Therefore, let's have a look at the individual elements of the program *SimpleC* and highlight the differences with R. In the first line we find a "preprocessor" directive, the "#include" part. This tells the compiler that we want to use functions from the "header files" called *R.h* and *Rmath.h* in our program. Header files are libraries that contain functions. When coding in C many function are not available by default as in R, but we can gain access to many C functions by including correct header files in the first lines of our code. Think of it as loading a package in R. Note that we don't need these header files in this example (it will run perfectly well without it). We just added the header files it as an example to show how you can include functions, even a bunch of functions you know and love from R, by loading pre-made libraries in your C functions. *R.h* and *Rmath.h* are among the most useful to include when extending R.

Next we see sections of code between "/*" and "*/", we can use these as we use # in R code, to comment sections for our convenience. The next important

line is where we start our C function, with "void". This line tells the compiler that there is a function named *SimpleC*, with an integer input "*int *nc*" and double (i.e. floating point) inputs "*double *answerc*" and "*double dnc*", and that the function should not return anything ("void"). Usually in C we would start with something like a main function, for instance the command "int main()" would make a function called main that returns an integer. However, R requires that C functions start with *void*, as they should not return anything [14]. The operations work by sending an empty vector (e.g. full of zeroes) from R to *SimpleC* called $*answer$ which is modified by *SimpleC*. We then access it in R in its modified state. It is called "$*answer$" (not "answer") because the "*" specifies that it is a pointer, which specifies the location of an R object in the memory [15].

We also see that, just as in R, our functions are encapsulated by "curly braces" ({ and }) which signal the start and end of functions or "if" statements. Inside the "curly braces", we see that as in R, input and data are stored in variables, however, in C one must declare the specific type of each variable before it is used. As seen above for instance, we create two integers variables with "*int n*" and "*int i*", where we explicitly declare not only the name, but also the type of data that it will contain (integers, or whole numbers without decimals). Some basic types include *char*, *int*, $float$ and *double* (*char* for characters, *int* for integers and $float$ and *double* for numbers with decimals). We then continue to assign values from the pointers N and dN, because we need real values for n and N for calculation. Remember that a pointer only tells us where an R object is in the memory, not its value (and we can't really calculate with memory locations of objects). Also, one should always double check the math in C code, so that we are sure that we are not dividing a double by an integer (which is why we created *double dnc* in addition to int *nc* in the above example).

One other thing you will notice is that the indexing of vectors starts at 0, so the first element of a vector V is not found by the command V[1], but with the command V[0]. In C and C++ vectors always start with 0, this is actually customary in computer science and many other programming languages (with R being the odd one in this sense). Next we see that for loops are initiated in a slightly different fashion with "$for(i = 0; i < n; i++)$". This specifies that the loop starts its iteration with i = 0 (corresponding to the first element of the vector), continues while i<n and is incremented at each iteration with 1 (i++). Finally, we end each line of code within a C program with a semi-colon ";".

[14]This is the case when we use C and $.C$ to call our function, as you will see later in a slightly more complex version: we can manipulate R objects and return them if we want to.

[15]Pointers literally "point" to locations in memory. So the vectors are first created in R, and then instead of copying every element of a potentially very large vector, we simply pass its location to the C function and proceed with our calculations.

5.3 Compiling C code and creating shared libraries

Now that we have some code written in C, we need to compile it and create a "shared library" from which it can be dynamically loaded into R. To do this we will need the standard compilers and libraries for our operating system [16]. Windows users will have acquired them in section 5.1.1. Mac users should download the Xcode application followed by the Command Line Tools package. Linux users should acquire the correct packages for doing so. On Ubuntu the correct packages can be installed with:

```
sudo apt-get update
sudo apt-get install build-essential r-base-dev
```

In many cases these will be already installed on a *UNIX system, so this should work without extra effort. Once you are certain all tools are ready, we can start. One advantageous way to code in C for usage in R, is to do so "inline" within your R scripts. This way everything is in one place that needs to be. One way to code C in R is through using the *sink* and *cat* commands. In this way we use R to create the files.

R Example 5.3.

```
## OnlineRcode59.R

## Open a connection to a file on the system and in
## the working directory
sink("Simple.c")

## Send C code to this file with "cat"
cat("
/* Rewrite of  InterpreterQuirks.R */
void SimpleC(int *nc, double *dnc, double *answerc){

 int n = nc[0];
 double dn = dnc[0];
 int i;

    for (i=0; i<n; i++){
      answerc[i] = (dn/(1+dn));
    }
}
")
```

[16] We need a compiler as it will translate the programs we write into an executable that the computer can "understand" and execute.

```
#stop writing Simple.c
sink(NULL)
```

If you copy and paste the above, you should have a file in your working directory called "*Simple.c*" that has the above C-code as its contents. You can use the command *file.show*("*Simple.c*") in R to confirm this. Now that we have this stored as a file, let us compile it and create a shared object that we can load in R. Again we can do this from R if we want to (or you can use any command line tools you have [17]. If you are working from the command line, which may be the easiest approach if you are a Mac user, you can create a shared library for R by typing (in the directory you saved *Simple.c*) "R CMD SHLIB -o Simple.so Simple.c". This commands calls R to create shared objects (for loading into R). It accepts as arguments a list of files with extensions .o .c, .cpp, and .f (which are object files, C, C++, or FORTRAN sources, respectively). For more details see the R documentation for writing R extensions [18]. We can do the same from R by invoking the system command (the following code is for Unix and Mac users and the next is for Windows users).

R Example 5.4.

```
## OnlineRcode60.R

system("R CMD SHLIB -o Simple.so Simple.c")
```

When using Windows you should change the ".so" into ".dll" or you may receive a warning:

R Example 5.5.

```
## OnlineRcode61.R

system("R CMD SHLIB -o Simple.dll Simple.c")
```

Now that we have compiled *Simple.c* into a shared object (*Simple.so*) or a "dynamic-link library" (Microsoft's implementation of shared objects: *Simple.dll*), which can be dynamically loaded in R, all we need to do before using it is to create a wrapper function in R. Crucial components of the wrapper function are 1) the R function that calls compiled C code *.C*() and 2) the *dyn.load*()

[17] These tools are included in the *Rtools* download for Windows see 5.1.1

[18] `http://stuff.mit.edu/afs/sipb/project/r-project/arch/i386_rhel3/lib/R/doc/manual/R-exts.html`

function which loads shared objects. A typical wrapper function (in Unix/Mac) will look like this:

R Example 5.6.

```
## OnlineRcode62.R


## change to dyn.load("Simple.dll") when on windows
dyn.load("Simple.so")

SimpleCWrapper <- function(N){

       out <- .C("SimpleC",
           nc     = as.integer(N),
           dnc    = as.double(N),
           answer = as.double(rep(0,N))
           )

       return(out$answer)
}
```

Within the function *.C* we call our C function *SimpleC*, followed by all the commands which need to be passed to it. However, Windows users will have created a *Simple.dll* file instead of a *Simple.so* file, so the wrapper function for Windows is:

R Example 5.7.

```
## OnlineRcode63.R

dyn.load("Simple.dll")

SimpleCWrapper <- function(N){

       out <- .C("SimpleC",
           nc     = as.integer(N),
           dnc    = as.double(N),
           answer = as.double(rep(0,N))
           )

       return(out$answer)
}
```

One thing that is very important is that we ensure that the variables we send to the compiled code correspond exactly to what is expected (e.g. types and names), or the code may crash. The final bit of the wrapper organizes the output of the function and returns what we are interested in. If you change your C code, always make sure to use *dyn.unload* to remove it. That's it! Now that we have a wrapper function, we are ready to test it:

R Example 5.8.

```
## OnlineRcode64.R

system.time(Canswers<-SimpleCWrapper(1e6))

##    user  system elapsed
##   0.008   0.000   0.008

system.time(Ranswers<-SimpleR(1e6))

##    user  system elapsed
##   1.396   0.004   1.400

identical(Canswers,Ranswers)

## [1] TRUE
```

We see that we have succesfully called a C function from R that gives us identical results to our R-version. Now that we have all the tools to extend R, we can start with a more serious example.

6 Calling C from R: Optimizing a population model

In the next and final section, we will apply all skills we learned so far to a practical problem in ecology and evolution: a stochastic population model. As specified in the general guidelines in section 1.2, we will start with slow code, then apply profiling, optimize our function first fully in R and only then continue towards compiled code. This is always the recommended approach (ref [3] in the main text), for any optimization problem.

6.1 A stochastic 2-species population model

We will start by building a simple model of the population dynamics of two species which compete for a common resource, where competition and growth rates are subject to random stochastic environmental variation. The equations describing the competitive dynamics are given by a standard Lotka-Volterra 2-species competition model:

$$\begin{aligned} \frac{dx_1}{dt} &= r_1 x_1 \left(1 - \left(\frac{x_1 + \alpha_{12} x_2}{K}\right)\right) \\ \frac{dx_2}{dt} &= r_2 x_2 \left(1 - \left(\frac{x_2 + \alpha_{21} x_1}{K}\right)\right). \end{aligned} \tag{6.1}$$

Here, (x_1) and (x_2) are the densities of species 1 and 2 respectively, α_{12} is a competition coefficient, representing the competitive effect of species 2 on species 1 (x_1) and α_{21} represents the effect species 1 has on species 2. In this version we will assume that each species has its own growth rate r_1 or r_2, but that the two species have equal constant carrying capacity (K). Growth rates and competition coefficients are distributed as follows:

$$\begin{cases} r_1 \sim norm(\mu_1, \sigma_1) \\ r_2 \sim norm(\mu_2, \sigma_2) \\ \alpha_{12} \sim norm(\mu_{12}, \sigma_{12}) \\ \alpha_{21} \sim norm(\mu_{21}, \sigma_{21}) \end{cases} \tag{6.2}$$

Let us imagine that our goal would be to follow the population changes of these two species on an evolutionary time scale, say 1 million generations. Therefore, before we continue to translate this population model into R, let's first set up some constants and parameters. We will set the simulation time, N, initially to 1% of our goal of 1 million, so we can rapidly profile our code.

R Example 6.1.

```
## OnlineRcode65.R

## Simulation time
N <- 10000
```

Next we need to set the model parameters: mean competition coefficients, variances and carrying capacity.

R Example 6.2.

```
## OnlineRcode66.R

alpha.means<-c(1.29,1.29)
alpha.sd<-c(0.01,0.01)
r.means<-c(1.03,1.03)
r.sd<-c(0.01,0.01)
CarryingCapacity<-1000
```

Now let's define a population model, at first crudely and naively coded.

R Example 6.3.

```
## OnlineRcode67.R

NaiveLotka<-function(T  = N,
                     am = alpha.means,
                     as = alpha.sd,
                     rm = r.means,
                     rs = r.sd,
                     K  = CarryingCapacity){

  pop<-data.frame(SP1=1,SP2=1,time=1)
  for(i in 2:T){
    SP1<-pop$SP1[i-1]*rnorm(1,rm[1],rs[1])*
      (1-(pop$SP1[i-1]+(rnorm(1,am[2],as[2])*pop$SP2[i-1]))/K)
    SP2<-pop$SP2[i-1]*rnorm(1,rm[2],rs[2])*
      (1-(pop$SP2[i-1]+(rnorm(1,am[1],as[1])*pop$SP1[i-1]))/K)
    pop<-rbind(pop,c(SP1,SP2,i))
  }

  return(pop)
```

```
}

## Save the function to a source code file for profiling
dump("NaiveLotka",file="NaiveLotka.R")
```

With the model function *NaiveLotka* defined, we can profile the code as we did earlier to find bottlenecks. At each optimization step we compared the results to earlier versions to ensure that these were correct (see Fig. 6.4). To do this, remember to set the same random seed every time before executing the program (use *set.seed*).

R Example 6.4.

```
## OnlineRcode68.R

## reload our program so everything matches up exactly
source("NaiveLotka.R")

## Switch on R's profiler
Rprof(file="NaiveLotka.out",line.profiling =TRUE)

## Set seed so we can reproduce our results
set.seed(1)

## Run NaiveLotka to start our simulation
ResultsNaive<-NaiveLotka()

## stop profiling
Rprof(append=F)
```

After profiling the *NaiveLotka* function, let's first plot the execution density of each code line to find the rough bottlenecks. See Figure 6.1 for the details.

R Example 6.5.

```
## OnlineRcode69.R

## Load Amdahl's profiler
require(aprof)
```

```r
## make an aprof object
NaiveLotkaAprof <- aprof("NaiveLotka.R","NaiveLotka.out")

## Plot the execution time per line
plot(NaiveLotkaAprof)
```

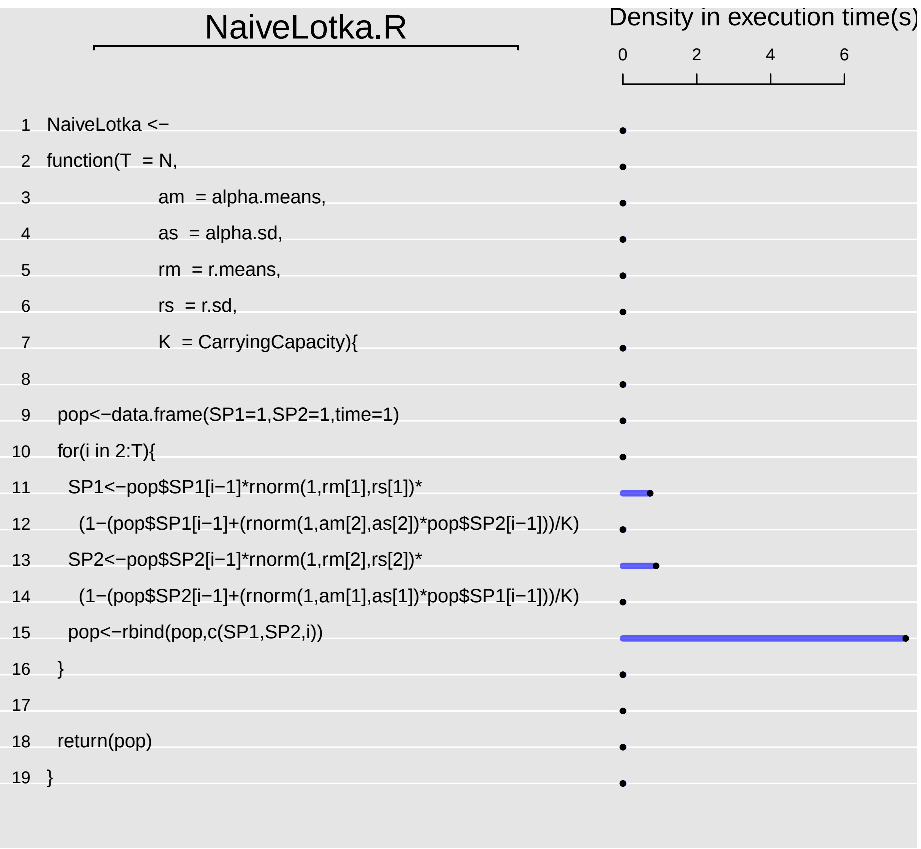

Figure 6.1: The time spent executing each line of the program NaiveLotka. We clearly see that the inefficient operations associated with growing the data.frame (line 15 of the code) are taking most of the system's resources.

Next let's find out whether optimization is theoretically worthwhile:

R Example 6.6.

```
## OnlineRcode71.R

summary(NaiveLotkaAprof)

## Largest attainable speed-up factor for the entire program
##
##         when 1 line is sped-up with factor (S):
##
##   Speed up factor (S) of a line
##              1     2     4     8     16    S -> Inf**
## Line*: 15 :  1.00  1.69  2.59  3.52  4.30  5.51
## Line*: 13 :  1.00  1.05  1.08  1.09  1.10  1.11
## Line*: 11 :  1.00  1.04  1.06  1.07  1.08  1.09
##
## Lowest attainable execution time for the entire program when
##
##              lines are sped-up with factor (S):
##
##   Speed up factor (S) of a line
##              1      2      4      8      16
## All lines    9.360  4.680  2.340  1.170  0.585
## Line*: 15 :  9.360  5.530  3.615  2.657  2.179
## Line*: 13 :  9.360  8.910  8.685  8.572  8.516
## Line*: 11 :  9.360  8.990  8.805  8.712  8.666
##
##     Total sampling time:  9.36  seconds
##  *  Expected improvement at current scaling
##  ** Asymtotic max. improvement at current scaling
```

The combination of the output from Fig. 6.1 and the table returned by *aprof* show clearly that 1) most time is spent in line 15, and 2) that optimization of line 15 is worthwhile. Hence we should stop growing data.frames with the *rbind* function. Therefore, let us improve our code.

R Example 6.7.

```
## OnlineRcode72.R

N=10000
 #start with pre-allocating the end results in the memory
```

```
population <- data.frame(SP1=numeric(N),SP2=numeric(N),T=1:N)
#set initial population
population[1,] <- c(1,1,1)
#define new function
LessNaiveLotka<-function( T   = N,
                          pop = population,
                          am  = alpha.means,
                          as  = alpha.sd,
                          rm  = r.means,
                          rs  = r.sd,
                          K   = CarryingCapacity){

   for(i in 2:T){
     pop$SP1[i]<-pop$SP1[i-1]*rnorm(1,rm[1],rs[1])*
       (1-(pop$SP1[i-1]+(rnorm(1,am[2],as[2])*pop$SP2[i-1]))/K)

     pop$SP2[i]<-pop$SP2[i-1]*rnorm(1,rm[2],rs[2])*
       (1-(pop$SP2[i-1]+(rnorm(1,am[1],as[1])*pop$SP1[i-1]))/K)
   }

   return(pop)
 }

## Save the function to a source code file
dump("LessNaiveLotka",file="LessNaiveLotka.R")
```

Now if we profile our new function *LessNaiveLotka* (go ahead and try it), and plot the output we will see that first we have reduced execution time by approximately 50%, and that most time is now spent within the actual calculations of competition. To be precise, most time is spent in lines 11 and 14. The output from the *aprof* function also suggests that focusing on optimizing both these lines would be worthwhile. However, as we can see (in Fig. 6.2) there are many functions within these lines. So which one is hogging our resources? To find out, we can use the function *targetedSummary* from the package *aprof* on lines 11 and 14.

R Example 6.8.

```
## OnlineRcode74.R

source("LessNaiveLotka.R")
```

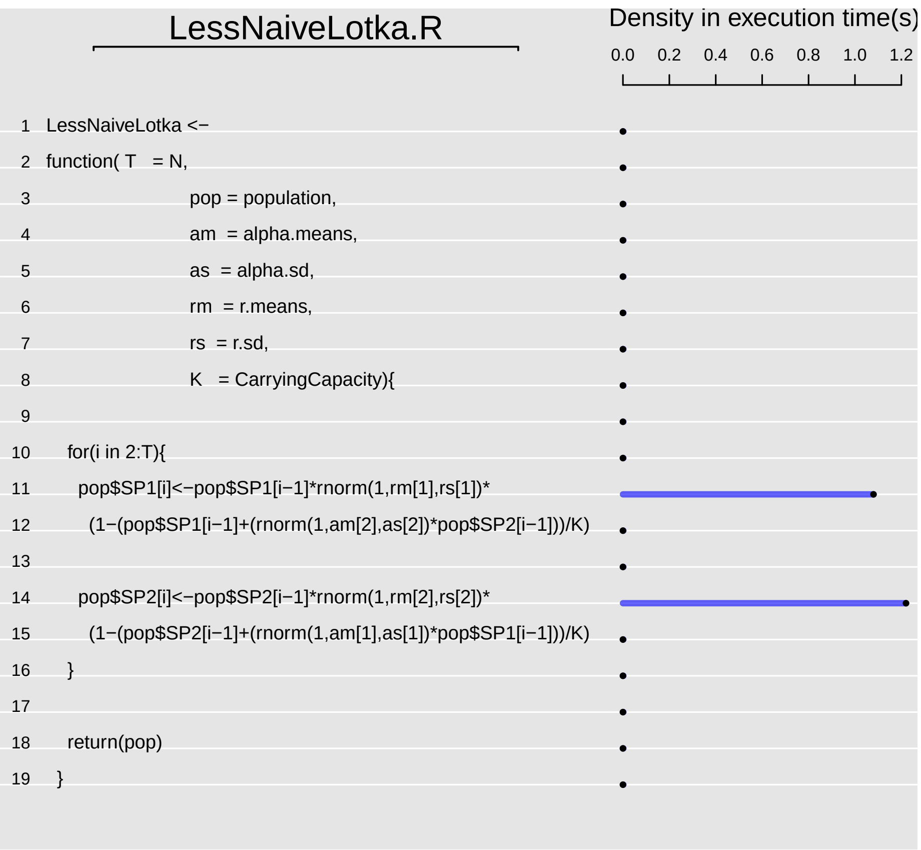

Figure 6.2: The time spent executing each line in our improved program LessNaiveLotka. Now we see that most time is spent in lines 11 and 14. However, which functions are taking up most of the execution time within these lines?

```
## Switch on R's profiler
Rprof(file="LessNaiveLotka.out",line.profiling =TRUE)

## Set seed so we can reproduce our results
set.seed(1)

## Run NaiveLotka to start our simulation
ResultsLessNaive<-LessNaiveLotka()

## stop profiling
```

```
Rprof(append=F)
```

R Example 6.9.

```
## OnlineRcode75.R

## Read in profiler output, and create aprof class
LessNaiveLotkaAprof <- aprof("LessNaiveLotka.R","LessNaiveLotka.out")

## take a closer look at lines 11 and 14
targetedSummary(target=11,LessNaiveLotkaAprof)

##          Function Calls Time
## 1    $.data.frame    30 0.60
## 2              [[    27 0.54
## 3   [[.data.frame    25 0.50
## 4            %in%    15 0.30
## 5           match     8 0.16
## 6     <Anonymous>     4 0.08
## 7           rnorm     4 0.08
## 8             all     3 0.06
## 9  $<-.data.frame     3 0.06
## 10       .subset2     2 0.04
## 11              *     1 0.02
## 12      .External     1 0.02
## 13          nargs     1 0.02
## 14       sys.call     1 0.02

targetedSummary(target=14,LessNaiveLotkaAprof)

##          Function Calls Time
## 1    $.data.frame    34 0.68
## 2              [[    29 0.58
## 3   [[.data.frame    27 0.54
## 4            %in%    11 0.22
## 5           match     9 0.18
## 6           rnorm     5 0.10
## 7     <Anonymous>     4 0.08
## 8       .External     4 0.08
## 9  $<-.data.frame     2 0.04
## 10       sys.call     2 0.04
## 11            all     1 0.02
## 12           NROW     1 0.02
```

Now we can narrow down on the slowest part of our program $LessNaiveLotka$.

We see that the operations surrounding our choice of data format are slowing things down. Just as was the case in section 2.1 our choice for using *data.frame* is causing us trouble. Remember that dataframes in R are very useful for storing multiple type of data (e.g. integers, characters, factors etc.). However we don't really need this functionality of a dataframe because we only use one type of data in this simulation. We can therefore switch to a far more efficient way of storing a single data type: matrices. Our code will require only a few changes:

R Example 6.10.

```
## OnlineRcode76.R

## switching from data.frame to use a matrix
population <- matrix(c(numeric(N),numeric(N),1:N),ncol=3,nrow=N,byrow=F)

## set initial population
population[1,] <- c(1,1,1)

MatrixLotka<-function(T   = N,
                      pop = population,
                      am  = alpha.means,
                      as  = alpha.sd,
                      rm  = r.means,
                      rs  = r.sd,
                      K   = CarryingCapacity){

  for(i in 2:T){
    pop[i,1]<-pop[i-1,1]*rnorm(1,rm[1],rs[1])*
      (1-(pop[i-1,1]+(rnorm(1,am[2],as[2])*pop[i-1,2]))/K)

    pop[i,2]<-pop[i-1,2]*rnorm(1,rm[2],rs[2])*
      (1-(pop[i-1,2]+(rnorm(1,am[1],as[1])*pop[i-1,1]))/K)
  }

  return(pop)
}

## Save the function to a source code file
dump("MatrixLotka",file="MatrixLotka.R")
```

Next we quickly find out how much we have gained, by profiling again.

R Example 6.11.

```
## OnlineRcode77.R
```

```
## reload our program so everything matches up exactly
source("MatrixLotka.R")

## Load Amdahl's profiler
require(aprof)

## Switch on R's profiler
Rprof(file="MatrixLotka.out",line.profiling =TRUE)

## reset seed and start simulation
set.seed(1)

## Run MatrixLotka
ResultsMatrix<-MatrixLotka()

## stop profiling
Rprof(append=F)
```

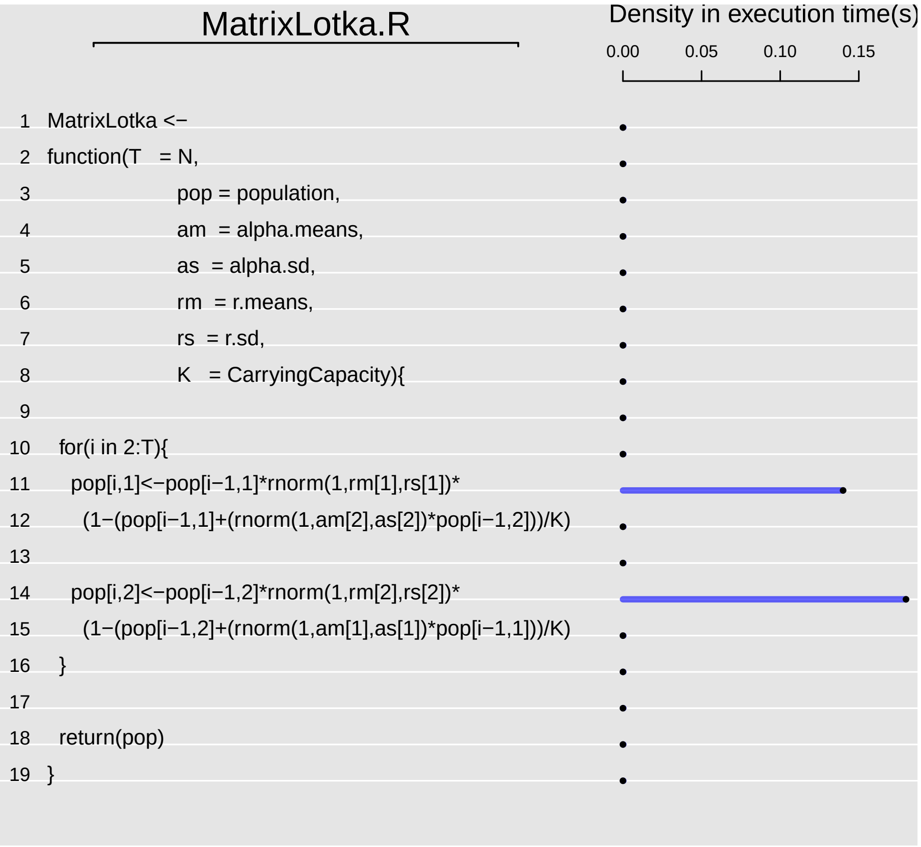

Figure 6.3: The time spent executing each line of our program MatrixLotka. The program has been sped up considerably, but again most time is spent in lines 11 and 14.

R Example 6.12.

```
## OnlineRcode79.R

## See what the potential returns of optimization are
MatrixLotkaAprof <- aprof("MatrixLotka.R","MatrixLotka.out")
summary(MatrixLotkaAprof)

## Largest attainable speed-up factor for the entire program
##
##         when 1 line is sped-up with factor (S):
##
##   Speed up factor (S) of a line
##              1     2     4     8     16    S -> Inf**
## Line*: 14 :  1.00  1.33  1.60  1.78  1.88  2.00
## Line*: 11 :  1.00  1.24  1.41  1.52  1.57  1.64
##
## Lowest attainable execution time for the entire program when
##
##              lines are sped-up with factor (S):
##
##   Speed up factor (S) of a line
##              1       2       4       8       16
## All lines    0.3600  0.1800  0.0900  0.0450  0.0225
## Line*: 14 :  0.3600  0.2700  0.2250  0.2025  0.1913
## Line*: 11 :  0.3600  0.2900  0.2550  0.2375  0.2287
##
##     Total sampling time:  0.36  seconds
##  *  Expected improvement at current scaling
##  ** Asymtotic max. improvement at current scaling
```

We see that these simple changes have sped-up our code by a whopping speed-up factor of 18 compared to the previous version! Clearly, choosing the correct way to store data and simulation results is something one should always consider. At this point our program is already quite efficient and would likely suit most purposes. However, for the sake of the exercise, let us continue to see if the calculations can be further optimized. If we plot the execution time per line of code, we again see in Fig. 6.3 that most time is spent executing lines 11 and 14. However, as these lines contain too many functions to easily identify the slowest parts, we will again look at these lines in detail. In the below code we use the function *readLineDensity* from *aprof* as an automatic way to find the two lines with the most calls (which on our laptop was 11 and 14).

R Example 6.13.

```
## OnlineRcode80.R

## Calculate line density
LineDens<-readLineDensity(MatrixLotkaAprof)
## sort line numbers descending
TopLines<-LineDens$Line.Numbers[order(1/LineDens$Call.Density)]

## take a closer look
targetedSummary(target=TopLines[1],MatrixLotkaAprof)

##    Function Calls Time
## 1     rnorm     6 0.12
## 2 .External     2 0.04

targetedSummary(target=TopLines[2],MatrixLotkaAprof)

##    Function Calls Time
## 1 .External     3 0.06
## 2     rnorm     3 0.06
```

Now we see something interesting, the profiler has only sampled the "call stack" - the list of active R functions - a handful of times (as can be seen in the number of calls). This is because our program executes too fast, we therefore don't have a decent view on what is taking most of the time. Depending on the speed of your machine you may even get an error returned, which relates from the program running so fast that no samples were made by R's profiler. There are two solutions to this; 1) increase the sampling interval in *Rprof* or 2) increase the simulation size. Let us do the latter to get a better picture of what is going on.

R Example 6.14.

```
## OnlineRcode81.R

## Rerun with more simulations so we get enough samples.
N=100000

## Pre-allocate
population <- matrix(c(numeric(N),numeric(N),1:N),
                                            ncol=3,nrow=N,byrow=F)

## Set initial population
```

```
population[1,] <- c(1,1,1)

## Reset seed and start simulation
set.seed(1)

## Switch on R's profiler
Rprof(file="LongMatrixLotka.out",line.profiling =TRUE)

## Run MatrixLotka
ResultsMatrix<-MatrixLotka()

## Stop profiling
Rprof(append=F)
```

Take a look again:

R Example 6.15.

```
## OnlineRcode82.R

## make aprof object
LongMatrixLotkaAprof <- aprof("MatrixLotka.R", "LongMatrixLotka.out")

## Calculate line density
LineDens <- readLineDensity(LongMatrixLotkaAprof)

## sort line numbers descending
TopLines <- LineDens$Line.Numbers[order(1/LineDens$Call.Density)]

## take a closer look
targetedSummary(target = TopLines[1], LongMatrixLotkaAprof)

##   Function Calls Time
## 1    rnorm    38 0.76
## 2        -     3 0.06
## 3        +     3 0.06
## 4        *     2 0.04
## 5        /     1 0.02

targetedSummary(target = TopLines[2], LongMatrixLotkaAprof)

##   Function Calls Time
## 1    rnorm    28 0.56
## 2        -     2 0.04
## 3        *     1 0.02
```

The program now takes slightly longer to finish, and so we have more samples to determine what functions are consuming the most resources in lines 11 and 14. In this case, slow downs are now caused by basic operators in R , $-, +, (,)$, and random number generation (*rnorm*). Usually these operators are very fast, at least when we use these in a vectorized fashion.

In this example the change of the population depends on its previous state, which must be calculated first, so we can't avoid the loop. However, since the distributions of the random numbers do not depend on the population state, the calls to *rnorm*() can be vectorized, which does speed up the simulation somewhat.

R Example 6.16.

```
## OnlineRcode83.R

MatrixLotka2 <- function(T = N, pop = population, am = alpha.means,
    as = alpha.sd, rm = r.means, rs = r.sd, K = CarryingCapacity) {

    r1 <- rnorm(T, rm[1], rs[1])
    a1 <- rnorm(T, am[1], as[1])
    r2 <- rnorm(T, rm[2], rs[2])
    a2 <- rnorm(T, am[2], as[2])

    for (i in 2:T) {
        pop[i, 1] <- pop[i - 1, 1] * r1[i] * (1 - (pop[i - 1, 1] +
            (a2[i] * pop[i - 1, 2]))/K)

        pop[i, 2] <- pop[i - 1, 2] * r2[i] * (1 - (pop[i - 1, 2] +
            (a1[i] * pop[i - 1, 1]))/K)
    }

    return(pop)
}
```

If more speed-up is needed, the only thing left to do is to drop towards a lower-level and compiled language (i.e. creating our own vectorized function). This is exactly what we will do in the next section.

6.2 The final step: the population model in compiled code

In the example in section 5.2 we made a very simple C program which we called in R with the function *.C*. Using the *.C* function was traditionally the way to call C code in R, in this our final optimization step, we will however use R's *.Call*

functionality [19]. You can think of *.Call* as the more sophisticated nephew of *.C*, which allows you do to much more within your C functions. In fact it allows a user to write C code using internal R data structures. Through the *.Call* interface, we can pass R objects directly to C, manipulate them, use functions to extract the information from them, return them to R, even create R objects in C and finally call R functions from C to use on our R object directly [20]. If one needs this functionality *.Call* is the way to go. However, the drawback is that it is more complex than using the *.C* interface. Therefore, before we continue to produce a replacement compiled code for our population model in R, we will first discuss some basic aspects. If any further questions arise on this issue we advise readers to consult the official ”Writing R Extensions” manual [21].

6.2.1 Working with R objects

Programming C code for usage via *.Call* is very similar to programming for the *.C* interface, but there are some clear differences. First, we need to call a specific header file, *Rinternals.h*, which includes definitions for using R's internal structures (in short ”the low level coding” behind R objects and variables) [22]. We will need this to create and manipulate R objects in C. The next thing to know is that functions used with *.Call* should always accept and return *SEXPs* (*SEXP* stands for ”S Expression” and is a structure originally defined by the R developers). Remember that in our previous example in 5.2 our C function started with *void*, which meant that the function did not return anything. Now, as we need to return a *SEXP*, all our C functions should therefore start with *SEXP*, as follows:

R Example 6.17.

```
## OnlineRcode84.R

/* Rcode72.R */
#include <R.h>
#include <Rmath.h>
#include <Rinternals.h>

SEXP OurCFunction(SEXP A, SEXP B, SEXP N) {

...
```

[19] It is strongly recommended to always start writing in pure R, then *.C* and then *.Call*.

[20] There are a couple of extra reason *.Call* would be preferred above *.C*, for instance when manipulating character vectors. See the ”Writing R Extensions” manual for more examples: http://cran.r-project.org/doc/manuals/R-exts.html

[21] http://cran.r-project.org/doc/manuals/R-exts.html.

[22] Readers who would like more information should consult http://cran.r-project.org/doc/manuals/R-ints.html.

```
}
```

After loading some key header files, our functions start with $SEXP$ and all inputs (A, B and N) are also declared as $SEXP$. We include the *Rinternals.h* header file (which is crucial for manipulating R internal structures) while the other header files (*Rmath.h*, *R.h*) include functions that we will use later. More information on all the header files available can be found here [23]. The next step is to put some operations into our C function. We will do this in the following two sections, where we will pass R objects to C, create R objects in C, return them to R and dabble with random numbers. After that we will have all the information we need to create our population model in C.

6.2.2 SEXPTYPES

Before we can pass R objects to C and create them in C, we need to know a little more about the different types of $SEXPs$. Table 6.2.2, shows a selection of some common data variables as they are known in R, C and their $SEXPTYPE$ equivalents. We will now use these in a C function below.

R Example 6.18.

```
## OnlineRcode85.R

/* Rcode73.R */
#include <R.h>
#include <Rmath.h>
#include <Rinternals.h>

SEXP OurCFunction(SEXP A, SEXP B) {

/* assign pointers to R objects */
double *a=REAL(A);
double *b=REAL(B);

/* create new R objects in C for result storage. */
SEXP RESULT;

/* protect and allocate R objects in C. */
/* and assign pointer. */
```

[23] http://cran.r-project.org/doc/manuals/R-exts.html#Organization-of-header-files. - Note that by reviewing the source code of a header file, one can also find out which functions are available.

R type	C equivalent	SEXPTYPE
character	char	STRSXP
integer	int	INTSXP
logical	int (bool in C99)	LGLSXP
numeric	double	REALSXP

Table 6.1: This table was adapted from a more complete set on `http://cran.r-project.org/doc/manuals/R-exts.html#Details-of-R-types`.

```
PROTECT(RESULT=allocVector(REALSXP,1));
double *result=REAL(RESULT);

....

/* unprotect R objects in C. */
UNPROTECT(1);

/* return our results to R */
return RESULT;

}
```

Here we see a few new commands. First you will notice the "vector accessors" functions also called "*MACROS*" (*REAL, INTEGER*)[24] which do what you expect, they access R objects of the same type. You will need to call them every time you want to access an R object (of the same type). This can get messy, as a bunch of *REAL* calls repeated every time we handle an R object won't improve legibility. A way around this is use pointers, as we do above.

Another important consideration is that after we create a variable in our C code (with *SEXP results*) , we assign its type and size with $allocVector(REALSXP, n)$ [25] within the $PROTECT$ function. This is because we are now working at such a low-level that we have to worry about housekeeping. That is R frequently checks if allocated memory is not being used, and then marks it as as re-usable, in a process called garbage collection [26]. We have to actively protect our new R objects from garbage collection. An important rule is that before we return our results, all the $PROTECTs$ called must be balanced with calls to $UNPROTECT$. Therefore, if we protect ten objects, we must call $UNPROTECT(10)$ before returning anything.

[24] http://cran.r-project.org/doc/manuals/r-release/R-exts.html#Vector-accessor-functions
[25] See the "Writing R Extensions" manual for more commands (e.g. *allocMatrix*).
[26] `http://cran.r-project.org/doc/manuals/R-exts.html#Garbage-Collection`.

6.2.3 Random number generators

The R random number generators and many probability distribution are callable from C [27]. This is an immensely useful feature for our stochastic population model. A key thing to remember when using the R random number generators is that we have to call *GetRNGstate*() and after values have been generated, call *PutRNGstate*(). These functions respectively read (or create) the random seed (the ".Random.seed" in R) and update it after use. Note that when one uses R's *rnorm* or any other rxxxx (random number) functions, as we do below, should also be enclosed in calls to GetRNGstate and PutRNGstate. A range of distribution functions are available [28], along with mathematical functions [29] and mathematical constants [30] required to create custom probability density functions. Note that these familiar distribution functions only become available by including the header file *Rmath.h*. Including this in our above example code we now have:

R Example 6.19.

```
## OnlineRcode86.R

/* Rcode74.R */
#include <R.h>
#include <Rmath.h>
#include <Rinternals.h>

SEXP OurCFunction(SEXP A, SEXP B) {

/* assign pointers to R objects */
double *a=REAL(A);
double *b=REAL(B);

/* create new R objects in C for result storage. */
SEXP RESULT;

/* protect and allocate R objects in C. */
/* and assign pointer. */
PROTECT(RESULT=allocVector(REALSXP,1));
double *result=REAL(RESULT);

/* use rnorm, in which we update the random number generator state*/
```

[27] see http://cran.r-project.org/doc/manuals/R-exts.html#Random_number_generation
[28] http://cran.r-project.org/doc/manuals/R-exts.html#Distribution-functions
[29] http://cran.r-project.org/doc/manuals/R-exts.html#Mathematical-functions
[30] http://cran.r-project.org/doc/manuals/R-exts.html#Mathematical-constants

```
    GetRNGstate();
    result[0] = rnorm(a[0],b[0]);
    PutRNGstate();

/* unprotect R objects in C. */
UNPROTECT(1);

/* return our results to R */
return RESULT;

}
```

The next steps are to compile this code and create a shared object. Then we load it in R to see if it works. After saving the above file as "OurCFunction.c", we ran this from the R console:

R Example 6.20.

```
## OnlineRcode87.R



## create shared object, windows users should remember
## to use OurCFunction.dll
system("R CMD SHLIB -o OurCFunction.so OurCFunction.c")

## load shared object
dyn.load("OurCFunction.so")

## set seed
set.seed(1)

## Execute our function
.Call("OurCFunction",A = as.double(0),B = as.double(1))

## [1] 0.4608

## test it with the equivalent R function
set.seed(1)
rnorm(1)

## [1] 0.4608
```

And that is it: we created our first C function in which we handled an R object, created R objects and used the random number generator. However, in the end, basically what we have done is created a less robust and less useful version of *rnorm*, which is a pretty useless accomplishment. Therefore why don't we continue with a more useful exercise, our population model.

6.2.4 The final model

Without any further delay, let's use all our new knowledge on the *.Call* interface and translate our model into C. The full model in C, coded "in line" for use in R, is:

R Example 6.21.

```
## OnlineRcode89.R

/*Stochastic Lotka example in C*/
#include <R.h>
#include <Rmath.h>
#include <Rinternals.h>


SEXP lotkac(SEXP time, SEXP alphamean, SEXP alphasd,
SEXP rmean, SEXP rsd, SEXP K)
{

 int n=length(time);
 int i;

 /* create new R objects in C. */
 SEXP P1, P2, result;

 /* protect and allocate R objects in C. */
 PROTECT(P1=allocVector(REALSXP,n));
 PROTECT(P2=allocVector(REALSXP,n));


 /* set list that returns results to R*/
 PROTECT(result = allocVector(VECSXP, 2));


 /* assign pointers to R objects */
 double *p1=REAL(P1);
 double *p2=REAL(P2);
 double *am=REAL(alphamean);
 double *as=REAL(alphasd);
 double *rm=REAL(rmean);
 double *rs=REAL(rsd);
```

```
 double *k=REAL(K);
 double R, A;

 /* set initial population sizes*/
 p2[0]=1.0;
 p1[0]=1.0;

 for(i=1; i<n; i++) {
 /* actual simulation, in which we update the random number generator*/

    GetRNGstate();
    R = rnorm(rm[0],rs[0]);
    PutRNGstate();
    GetRNGstate();
    A = rnorm(am[1],as[1]);
    PutRNGstate();

    p1[i] = p1[i-1] * R *(1-(p1[i-1]+(A*p2[i-1]))/k[0]);


    GetRNGstate();
    R = rnorm(rm[1],rs[1]);
    PutRNGstate();
    GetRNGstate();
    A = rnorm(am[0],as[0]);
    PutRNGstate();

    p2[i] = p2[i-1] * R *(1-(p2[i-1]+(A*p1[i-1]))/k[0]);
 }

/* unprotect R objects in C. */

 SET_VECTOR_ELT(result, 0, P1);
 SET_VECTOR_ELT(result, 1, P2);
 UNPROTECT(3);

 return result;
}
```

We saved the above code as ”lotka.c” in our working directory. Most of the above code should be familiar by now, but we included a few new pieces of code. The first we see is *length*(*time*), which returns the length of *SEXP time*, *length*() is one of the many useful R functions we can call in C (when we include the

correct header files [31]). We also see the statement *results allocVector(VECSXP, 2)*, with this we define a new SEXP [32] as a list, with which we are able to return multiple results. We use this to return both population vectors, $P1$ and $P2$, specifying the population state at each time step by setting them as objects in the list *results*. This is done with the command *SET_VECTOR_ELT(result, 0, P1)* and *SET_VECTOR_ELT(result, 1, P2)*. These set $P1$ as the first element of the list and $P2$ as the second. Next, let's continue by executing (e.g. "copying") the above into R. We now should have a file called lotka.c in our working directory. We can now compile our program, and create a shared object.

R Example 6.22.

```
## OnlineRcode90.R

system("R CMD SHLIB -o lotka.so lotka.c")
```

Again, don't forget to change *lotka.so* to *lotka.dll* in the code if you are a Windows user. We then load our shared object, and create a more user friendly wrapper function. Finally, we time it to see what all the effort has gained us.

R Example 6.23.

```
## OnlineRcode91.R

dyn.load("lotka.so")

LotkaCWrapper<-function(time=1:N,alphamean=alpha.means,
alphasd=alpha.sd,rmean=r.means,rsd=r.sd, K=CarryingCapacity)

{
   out <- .Call("lotkac",
   time=as.double(time),
   alphamean=as.double(alphamean),
   alphasd=as.double(alphasd),
   rmean=as.double(rmean),
   rsd=as.double(rsd),
   K=as.double(K))
```

[31] Functions can be found by searching through the source code of the different header files, by looking through C code written by the R developers or by reading the manual - see e.g. `http://cran.r-project.org/doc/manuals/R-exts.html#Utility-functions`

[32] See for more types `http://cran.r-project.org/doc/manuals/R-exts.html#Details-of-R-types`

Program	NaiveLotka	MatrixLotka	LotkaCWrapper
Execution time	18 hours and 24 minutes	28.7 seconds	4.6 seconds

Table 6.2: The end gains. Required execution time for each of the previously defined programs to conduct a single simulation of the stochastic Lotka-Volterra population model over 1 million generations.

```
          return(out)
}

## reset seeds for simulations
set.seed(1)

#Set N to 10000 to compare with previous results
N=10000
#time the function

LotkaCT<-system.time(
ResultsC <- LotkaCWrapper()
)[3]

print(LotkaCT)

## elapsed
##    0.01
```

We see that when N is 10000, using C proved to be $\pm$ 34 times faster than our most efficient R program. When comparing it to our inefficient first naive R code, we actually sped things up by a factor 1016 times! This a serious speed-up, that shows that exactly the same results are possible in only a fraction of the time (see Fig. 6.4).

6.3 The end gains

We have gone through much effort to optimize our code, so let's see if it was worth it. Table 6.2 below shows the benchmark results when executing the full problem (our model for 1 000 000 years) for our code versions NaiveLotka, MatrixLotka and our final C version called through the wrapper function *LotkaCWrapper*.

In the end we gained a spectacular improvement above our original code for the full problem, with the C version finishing within 4.6 seconds compared to 18.4 hours for the original R version. That is a speed-up factor of more than

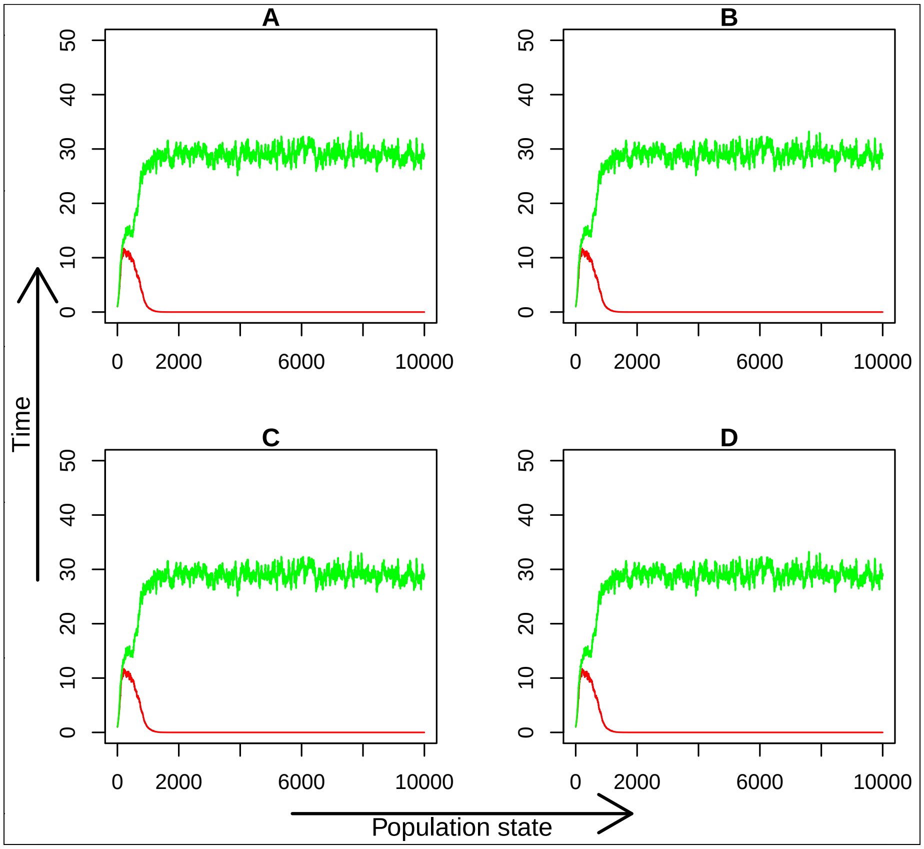

Figure 6.4: The results for all four version of our population model, the panels A, B, C and D show the results from our program version NaiveLotka, Less-NaiveLotka, MatrixLotka and LotkaC(Wrapper), respectively. The green and red lines indicate the populations for species 1 and 2. We see that in each different version of our program the results are identical and reproducible. The only thing that differs greatly is the time you need to wait to get these results.

14 000! Regardless of the C execution we also see that by applying simple corrections to our code (pre-allocating data and using an appropriate data storage class: matrix), we already went from 18.4 hours to 28.7 seconds. This really shows that learning how to program efficiently is worthwhile: non-trivial gains in efficiency are possible with relatively very little effort.

6.4 Summary

In this example, we optimized a simple stochastic two-species Lotka-Volterra competition model. It includes the operations used in many ecological and

evolutionary simulations: basic mathematical operations, randomly sampling statistical distributions, and saving simulation results in large datasets. Additionally, as change depends on the state of the population in a previous time step (a Markovian process), a single run cannot be conducted in parallel. We start with a naively coded example where we grow a dataset and consecutively profiled and optimized the algorithm in the following steps:

1. Profiling showed that most time was spent growing our results, we therefore switched to pre-allocating a data.frame in the memory.
2. Most time was then spent manipulating a data.frame, as the problem concerns numeric data we don't need the functionality of a data.frame (which can store many data types in one object). We therefore switched to the simpler matrix class for data storage.
3. Finally, we refactored the model in C, calling our C function from R.

As with the bootstrap example, we record the execution time with increasing time-steps and illustrate that the potential gains increase well beyond this with the number of computations (Table 6.3).

Naive R	R	Byte Compiler	C
1 minute	37.81 ms	18.08 ms	5 ms
1 hour	3 sec	1.5 sec	38 ms
1 day	15 sec	7.75 sec	2 sec
1 month*	83.89 sec	44.98 sec	11 sec
1 year*	4.95 min	2.73 min	1 min

Table 6.3: Observed and projected execution times for the different code versions of a two species stochastic population model. Each row shows the expected execution time for each code version using the slowest code as a baseline. Time units are denoted by milliseconds ("ms"), seconds ("sec") and minutes ("min"). *Statistics in these rows are predicted from fitted trends and were not timed directly.

6.5 Useful tools

At the end of this tutorial, we list a handful of very useful tools. We hope that these tools may be of use when trying to speed-up code or working with compiled code in R.

6.5.1 The R Byte Compiler

A very neat and relatively new tool with which to quickly speed-up many (but sadly not all) problems, is the use of the R-byte compiler. This tool translates R

code into more compact numeric ”byte codes”. These form instruction sets that are specifically designed for efficient execution by the interpreter. If we were to use the byte compiler on our function $MatrixLotka$ from earlier we would have obtained a speed-up of roughly 200%, which is not bad at all, but still much slower than calling our C function.

R Example 6.24.

```
## OnlineRcode93.R

N=10000

## reset seed and start simulation
set.seed(1)

ByteCompileLotka<-compiler::cmpfun(MatrixLotka)

## Time its execution
system.time(ResultsByteCompile <- ByteCompileLotka())

## user  system elapsed
##  0.180   0.000   0.179
```

6.5.2 Rccp

Once you start moving on from this tutorial and start extending your R code with more complex functions in C, you may run into issues with the translation or transferring of more complex data (than in our example) from R to your C and C++ programs. A great tool to makes this (and more) much easier is *Rcpp*. *Rcpp* is an R package that strives to make it easy for anyone who wants to connect C or C++ to R. *Rcpp* makes it straightforward to pass data from R to C++, and vice versa.

Additionally the *Rccp* community provides some informative tutorials on how to use *Rccp* to extend R; we recommend these by the *Rccp* developers [33] and Hadley Wickam [34]. We use *Rcpp* to optimize an evolutionary model in online text S3, achieving a considerable speed-up, as shown in the main document.

6.5.3 Profilers for compiled code

For those who will continue to write more seriously compiled code there are a few profilers available for compiled code. These should prove useful if you really

[33] http://cran.r-project.org/web/packages/Rcpp/vignettes/Rcpp-introduction.pdf
[34] http://adv-r.had.co.nz/Rcpp.html

need to squeeze every bit of inefficiency out of a program [35].

6.6 Closing remarks

Writing efficient code poses both obvious and subtle challenges. However, making the most of available computing resources can provide tremendous improvements in speed for a wide variety of common problems. Our benchmark results show that efficiency gains of orders of magnitude are possible (e.g. Tables 3.1 & 6.2). Code optimization can therefore contribute more to research projects than simply decreasing the waiting time for an analysis to finish. With greater efficiency in code, researchers can explore more complex models and datasets, better test the sensitivity of models to assumptions, and extend models to larger scales (spatial, taxonomic, or temporal).

If you are wondering where to go from here? How can you futher improve your code and programming skills? Our answer would be: share your code! Rocchini & Neteler (2012) advocated open code in ecology and active sharing among ecologists and we underscore this here. Free repository hosting services, such as GitHub (github.com), in combination with open-source and popular platforms like R help facilitate code sharing. Code sharing, in turn, provides an opportunity to reach out to other scientists for code review, a highly cost-effective way to eliminate bugs and improve code (ref [3] in the main text). Simultaneously, these sources enable discussing and learning coding approaches with other scientists with a range of coding expertise. Code sharing is therefore a highly effective way to keep track of advances in computational strategies that are constantly evolving. As these advances are more accessible today than ever before, we encourage biologists to benefit from code sharing by sharing ecological code on repositories like GitHub (where the code for the package *aprof* can be freely accessed).

The goal of this document was to show the reader some basic techniques on how to identify bottlenecks and speed-up R code. We hope that we have succeeded in this aspect and that some knowledge can be gained from the examples we gave, enough for researchers to apply these methods to their own work. We hope to have showed that the tools presented here can expand the feasibility of large, computationally complex and sophisticated modeling projects today. Thinking bigger is possible.

References

Adler, D., Gläser, C., Nenadic, O., Oehlschlägel, J. & Zucchini, W. (2014) *ff: memory-efficient storage of large data on disk and fast access functions.* R package version 2.2-13.

[35] `http://cran.r-project.org/doc/manuals/r-release/R-exts.html#Profiling-compiled-code`

Bohrer, G., Katul, G.G., Walko, R.L. & Avissar, R. (2009) Exploring the effects of microscale structural heterogeneity of forest canopies using large-eddy simulations. *Boundary-Layer Meteorology*, **132**, 351–382.

Buckner, J., Seligman, M. & Buckner, M.J. (2013) Package "gputools".

Chambers, J.M. (2009) *Software for Data Analysis: Programming with R.* Springer.

Clark, J.S., Bell, D., Chu, C., Courbaud, B., Dietze, M., Hersh, M., HilleRisLambers, J., Ibáñez, I., LaDeau, S., McMahon, S. *et al.* (2010) High-dimensional coexistence based on individual variation: a synthesis of evidence. *Ecological Monographs*, **80**, 569–608.

de Jonge, E., Wijffels, J. & van der Laan, J. (2014) *ffbase: Basic statistical functions for package ff.* R package version 0.11.3.

Eddelbuettel, D. & François, R. (2011) Rcpp: Seamless r and c++ integration. *Journal of Statistical Software*, **40**, 1–18.

Gause, G.F. (1934) *The Struggle for Existence.* Baltimore, MD: Williams & Wilkins.

Grama, A. (2003) *Introduction to Parallel Computing.* Pearson Education.

Hager, G. & Wellein, G. (2010) *Introduction to High Performance Computing for Scientists and Engineers.* CRC Press.

Hannay, J.E., MacLeod, C., Singer, J., Langtangen, H.P., Pfahl, D. & Wilson, G. (2009) How do scientists develop and use scientific software? *Proceedings of the 2009 ICSE Workshop on Software Engineering for Computational Science and Engineering*, pp. 1–8. IEEE Computer Society.

Hennekens, S.M. & Schaminée, J.H.J. (2001) TURBOVEG, a comprehensive data base management system for vegetation data. *Journal of Vegetation Science*, **12**, 589–591.

Jones, M.B., Schildhauer, M.P., Reichman, O.J. & Bowers, S. (2006) The new bioinformatics: Integrating ecological data from the gene to the biosphere. *Annual Review of Ecology, Evolution, and Systematics*, **37**, 519–544.

Kelling, S., Hochachka, W.M., Fink, D., Riedewald, M., Caruana, R., Ballard, G. & Hooker, G. (2009) Data-intensive science: a new paradigm for biodiversity studies. *BioScience*, **59**, 613–620.

Knaus, J., Porzelius, C., Binder, H. & Schwarzer, G. (2009) Easier parallel computing in r with snowfall and sfcluster. *The R Journal*, **1**, 54–59.

L'Ecuyer, P. (1999) Good parameters and implementations for combined multiple recursive random number generators. *Operations Research*, **47**, 159–164.

L'Ecuyer, P. (2012) *Random number generation.* Springer.

Ligges, U. (2006) R help desk: Accessing the sources. *R News*, **6**, 43–45.

Lotka, A.J. (1925) *Elements of Physical Biology.* Baltimore, MD: Williams & Wilkins.

Luo, Y., Ogle, K., Tucker, C., Fei, S., Gao, C., LaDeau, S., Clark, J.S. & Schimel, D.S. (2011) Ecological forecasting and data assimilation in a data-rich era. *Ecological Applications*, **21**, 1429–1442.

Michener, W.K. & Jones, M.B. (2012) Ecoinformatics: supporting ecology as a data-intensive science. *Trends in Ecology and Evolution*, **27**, 85–93.

Nathan, R., Katul, G.G., Bohrer, G., Kuparinen, A., Soons, M.B., Thompson, S.E., Trakhtenbrot, A. & Horn, H.S. (2011) Mechanistic models of seed dispersal by wind. *Theoretical Ecology*, **4**, 113–132.

Nvidia (2007) Compute unified device architecture programming guide.

Petrovskii, S. & Petrovskaya, N. (2012) Computational ecology as an emerging science. *Interface Focus*, **2**, 241–254.

R Core Team (2013) *R: A Language and Environment for Statistical Computing.* R Foundation for Statistical Computing, Vienna, Austria. ISBN 3-900051-07-0.

Rocchini, D. & Neteler, M. (2012) Let the four freedoms paradigm apply to ecology. *Trends in Ecology and Evolution*, **27**, 310–311.

Schmidberger, M., Morgan, M., Eddelbuettel, D., Yu, H., Tierney, L. & Mansmann, U. (2009) State-of-the-art in parallel computing with r. *Journal of Statistical Software*, **47**.

Tierney, L., Rossini, A., Li, N. & Sevcikova, H. (2013) Snow: simple network of workstations. *R package version 03-12, URL http://CRAN R-project org/package= snow.*

Urbanek, S. (2011) multicore: Parallel processing of r code on machines with multiple cores or cpus. *R package version 01-3, URL http://www rforge net/multicore.*

Volterra, V. (1926) Variazioni e fluttuazioni del numero d'individui in specie animali conviventi. *Mem R Accad Naz dei Lincei*, **2**, 31 − 113.

Weston, S. & Computing, R. (2013) foreach: Foreach looping construct for r. *R package version*, **1**.

Wilson, G., Aruliah, D., Brown, C.T., Hong, N.P.C., Davis, M., Guy, R.T., Haddock, S.H., Huff, K., Mitchell, I.M., Plumbley, M.D. *et al.* (2012) Best practices for scientific computing. *arXiv preprint arXiv:12100530.*

Wilson, G.V. (1995) *Practical Parallel Programming*, volume 15. MIT Press.
