# Supplementary material for: Speeding Up Ecological and Evolutionary Computations in R; Essentials of High Performance Computing for Biologists
Source: PLoS Comput Biol. 2015 Mar 26;11(3):e1004140. doi: 10.1371/journal.pcbi.1004140 (PMC4374948; doi:10.1371/journal.pcbi.1004140)
Supplement: S2 Text — (PDF) [file pcbi.1004140.s002.pdf]

S2 Text: Optimizing a spatial population model of plant invasion

Marco D. Visser*[1,2], Sean M. McMahon†[3], Cory Merow‡[3,4], Philip Dixon§[5], Sydne Record¶[6], and Eelke Jongejans‖[1]

[1]Departments of Experimental Plant Ecology and Animal Ecology & Ecophysiology, Radboud University Nijmegen, the Netherlands
[2]Smithsonian Tropical Research Institute, Panama
[3]Smithsonian Environmental Research Center, Edgewater, USA
[4]Department of Ecology and Evolutionary Biology, University of Connecticut, USA
[5]Department of Statistics, Iowa State University, USA
[6]Department of Biology, Bryn Mawr College, USA

January 28, 2015

Contents

1 Model setup **2**

2 Summary of optimization **3**

3 Functions for simulation **4**

4 Step 1: Initial Run **8**

5 Step 2: Change data frames to matrices **11**

6 Step 3: Parallelize **13**

*m.visser@science.ru.nl
†mcmahons@si.edu
‡cory.merow@gmail.com
§pdixon@iastate.edu
¶srecord@brynmawr.edu
‖e.jongejans@science.ru.nl

The following sections document the steps taken to optimize the code from Merow et al. (ref [10] in the main text), shown in Figure 3-C in the main document. The methods used to speed-up this example have been largely covered in the previous appendices (Text S1, sections 1-6). The interested reader is welcome to follow these steps as a case study.

1 Model setup

This example comes from an individual-based simulation predicting the spread of an invasive plant across a landscape as a function of time (described in detail in Merow et al. 2011). This model predicts how land use, population dynamics, dispersal by birds, and random long distance dispersal interact to determine the annual spread of Oriental bittersweet across New England (northeastern North America). The landscape consists of 3,057 5' by 5' cells and spread is simulated over 90 years using an annual time step. At each step, the population size in each cell is predicted. See Fig. 1.1 for a graphical illustration of the model. The simulation is stochastic, so 100 replicate simulations were run for analysis. For brevity here, we use only 20 replicates and report the percentage increase in efficiency for each iterative modification of the code. The predictions of the model are shown in Fig. 1.2.

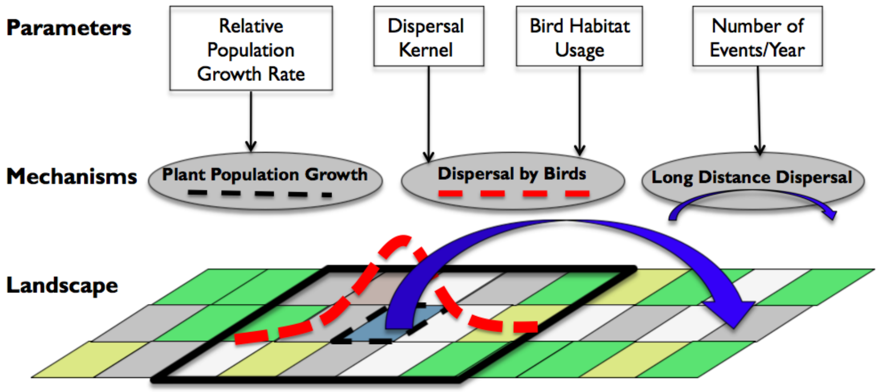

Figure 1.1: A graphical illustration of the simulation model for invasion dynamics.

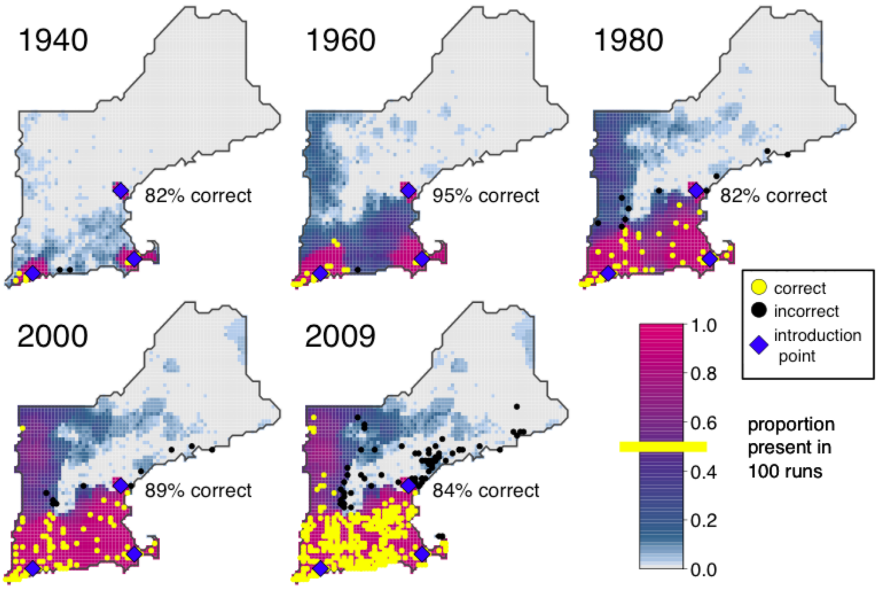

Figure 1.2: Predictions of the simulation model for invasion dynamics.

The outline for this example is as follows: (1) we begin by briefly presenting the code; (2) we show profiling output for the published version of the model (Merow et al. 2011); (3) we change the object storing the population sizes in each landscape cell from a data frame to a matrix to increase speed by approximately 350%; (4) we use a very simple parallelization protocol over replicate simulations to increase speed by an additional $\pm$ 300%.

A critical component of this example is that it requires almost no modification of the fundamental attributes of the part of the code that performs the simulations. That is, the modifications we illustrate change the overall structure of the computation, but do not change the details of the simulation code. This observation illustrates how many computational speed-ups do not require that one become an expert coder focused on optimizing every line of code, but that even structural modifications to 'clunky' and awkward code such as this (C. Merow's first attempt to learn programming) can drastically decrease computation time.

2 Summary of optimization

We made the following improvements, in each step we expedited computation while changing just a few lines of code:

1. Profiling identified the manipulation of *data.frames* as a major bottleneck, and we therefore changed the use of *data.frame* to *matrix*, achieving a speedup factor of 3.5 while changing only a single line of code.

2. We then create a parallel algorithm that executes replicate simulations simultaneously, achieving a speedup factor of 2.99 above the previous improvement. This was 10.5 time faster than the original code.

3 Functions for simulation

Understanding the computational speedups we illustrate fortunately only requires a conceptual understanding of the code (further details are presented in Merow et al. 2011). Below we introduce two functions: the first function (*dispersal.probs*) computes the distance-weighted dispersal probabilities for a seed to disperse from each source cell to each target cell in its respective neighborhood, while the second function (*cmstarling3dd*) performs the simulation of temporal spread by starling birds. The spread function begins by initializing the landscape with three observed populations, and each year simulates population growth, dispersal by birds, and random long distance dispersal. These functions are not modified during our illustration of computational speed-ups.

```
#The following file contains a matrix called 'nelandscape'.
#The first two columns give coordinates of each cell on the 82 x 79 grid
#overlayed on the landscape with (1,1) being the southwest corner. The third
#column gives the LULC classification (1= water, 2=developed, 3=agriculture,
#4=deciduous, 5=coniferous).

#included data file from supplementary materials
(load('nelandscape-appendix.RData'))

## [1] "nelandscape"

  #FUNCTION FOR DISPERSAL PROBABILITIES
        # determines the probability of being dispersed to each site from site i,j
dispersal.probs=function(landscape,maxdist,rate){
                # produces a distance weighted dispersal probability for each cell.
                #this function is always called outside of the cmstarling function
        lat=max(landscape[,2])
        long=max(landscape[,1])
                #avoids calculation at unsuitable sites
        good.sites=which(!landscape[,'lulc']==1)
                #make a matrix of distance weights
        weights=matrix(0,2*maxdist+1,2*maxdist+1)
        center=c(maxdist+1,maxdist+1)
        for(i in seq(1,2*maxdist+1)){
                for(j in seq(1,2*maxdist+1)){
                        weights[i,j]=dexp( (i-center[1])^2 +(j-center[2])^2-.5,rate )
```

```
                }
        }
        weights[center[1],center[2]]=0
        weights=weights/sum(weights)

        disp.matrix=array(0,c(nrow(weights),ncol(weights),2,lat*long))
        disp.matrix[,,1,good.sites]=weights
        # make another matrix of cell indices to go with each weight
        for( k in good.sites){
                xx=(landscape[k,'xi']-maxdist):(landscape[k,'xi']+maxdist)
                yy=(landscape[k,'xj']-maxdist):(landscape[k,'xj']+maxdist)
            for(i in xx[xx>0 & xx<=long]){
                for(j in yy[yy>0 & yy<=lat]){
                        disp.matrix[which(xx==i),which(yy==j),2,k]=
            which(landscape[,1]==i & landscape[,2]==j)
                }
            }
    }
        return(disp.matrix)
}


  #FUNCTION FOR SIMULATIONS
cmstarling3dd=function(birddisp,landscape,lat0,long0,generations,rate,
  plantprefs, carrying.cap,local.growth=TRUE, long.distance=TRUE,birds=TRUE,
  num.long.dist=1, recordtimes){
        #birddisp:an array giving the probability of bird dispersal from
    #each source cell to its respective bird dispersal neighborhood
        #landscape: matrix with columns giving x coords, y coords and LULC
    #for each cell
        #lat0, long0: initial populations
        #generations: # of time steps to iterate model
        #rate: 1/mean of the exponential dispersal kernel
        #plantprefs: a vector of population growth rates where the first element
    #corresponds to LULC class 1, etc.
        #carrying.cap: carrying capacity in a single cell
        #local.growth: logical - include local growth?
        #long.distance: logical - include LDD?
        #birds: logical - include local bird dispersal?
        #num.long.dist: # LDD events/year
        #recordtimes: steps where a snapshot is take of population distribution
                #########   GENERAL     ############################################
        init=cbind(long0, lat0)
        lat=max(landscape[,2])
```

```
  long=max(landscape[,1])
          #record keeping variables, state variable for each cell
  p=cbind(landscape,rep(0,lat*long))
  #storage
  timeseries=matrix(0,lat*long,length(recordtimes) )
  ts.counter=1 # counter for recording intervals
          # avoids calculation at unsuitable sites (ocean)
  good.sites=which(!landscape[,'lulc']==1)
  for(i in 1:nrow(init)){ #plants 1st populations
          p[which(landscape[,1]==init[i,1] & landscape[,2]==init[i,2]),4]=
carrying.cap/2
  }

          ########## DISPERSE SEEDS ############################
  for (t in 1:generations){
                  #put a population at Durham, NH in 1938
          if(t==14){p[which(landscape[,1]==35 & landscape[,2]==28),4]=
  carrying.cap/2}
                  #LOCAL GROWTH _____________________________________
          populated.sites=which(p[,4]>0)
          new.individuals=p[populated.sites,4]*(plantprefs[p[populated.sites,3]]-1)
          p[which(p[,3]==1),4]=0 #kill at unsuitable (ocean) sites
          if(local.growth){
                  p[populated.sites,4]=ceiling(pmin(carrying.cap,
                  p[populated.sites,4]+
    as.numeric(plantprefs[p[populated.sites,3]]>=1)
    *new.individuals*pexp(.5,rate)
    +as.numeric(plantprefs[p[ populated.sites,3]]<1)
    *new.individuals))
      }
                  #RANDOM LONG DISTANCE DISPERSAL ______________________
          if(long.distance){
                  for(i in 1:num.long.dist){
                          target.site=sample(which(p[,3]>1),1)
                          p[target.site,4]=min(carrying.cap,p[target.site,4]+1)
                  }
          }
                  #BIRD DISPERSAL _____________________________________
                  #set up the # of offspring from each site
          emigrate=rep(0,length(p[,4]))
          emigrate[populated.sites]=new.individuals
          emigrate=pmax(0,emigrate) #get rid of sink pops
          if(birds){
                  num.bird.seeds=emigrate*(1-pexp(.5,rate))
```

```
                    for(k in which(round(num.bird.seeds,0)>0)) {
                            newsite=try(sample(subset(c(birddisp[,,2,k]),
             c(birddisp[,,2,k]>0)),round(num.bird.seeds[k],0),
             prob=subset(c(birddisp[,,1,k]),
             c(birddisp[,,2,k]>0)) ,replace=T),TRUE) #get the new sites
                    p[newsite,4]=pmin(carrying.cap, p[newsite,4]+1) #place the
        #seeds in the new sites
                    }
            }
                    #_____________________________________________________________
                    #for specified time intervals, store the population matrix
            if(any(t==recordtimes)){
                    # timeseries[ ,ts.counter]=p[1:6478 ,4]
                    timeseries[ ,ts.counter]=p[,4]
                    ts.counter=ts.counter+1
            }
  } # time loop
        list(timeseries=timeseries)
}
```

To perform a simulation, we set a variety of parameter values, explained in-line in the code below. Note that the object *bird.disp* multiplies the distance-weighted dispersal probabilities by the bird's habitat preferences to depict a heterogeneous landscape (i.e. seeds are less likely to be dispersed into deciduous habitat than agricultural).

```
landscape=nelandscape
# the following correspond to the LULC types: (ocean,
# developed, agriculture, deciduous, coniferous)
birdtime=c(0,.39,.44,.06,.11)

        # MAKE DISPERSAL PROBABILITIES ----------------------------------------
        # this section takes a minute or two
maxdist=3       #sets size of local bird dispersal neighborhood in units of cells
rate=3.5        #1/mean dispersal distance, in units of cells
        #this calculates the distance weighted probabilities of dispersal
dist.mat=dispersal.probs(landscape,maxdist,rate)
        #this weights distances by bird habitat use
a=0*dist.mat[,,1,]
for( i in which(!landscape[,3]==1 & !landscape[,3]==0)) {
```

```
        for( j in 1:(2*maxdist+1)){
                for(k in 1:(2*maxdist+1)){
                        if(!dist.mat[j,k,2,i]==0){
                                a[j,k,i]=dist.mat[j,k,1,i]*
          birdtime[landscape[dist.mat[j,k,2,i],3]]
                        }
                }
        }
        a[,,i]=a[,,i]/sum(a[,,i])
}
birddisp=dist.mat
birddisp[,,1,]=a

lat=max(landscape[,2]); long=max(landscape[,1])
#PARAMETERS
#these correspond to the LULC types
#(water, developed, agriculture, deciduous, coniferous)
plantprefs=c(0, 2.1, 1.5, 1.4, .5)

 # specify the time steps at which to record the results
recordtimes=c(21,41,61,81,90)
generations=90
num.long.dist=1 # # of seeds per year for long distance dispersal
local.growth=TRUE
long.distance=TRUE
birds=TRUE
long0=c(9,39);  lat0=c(5,9)  #initial conditions
carrying.cap=200
```

4 Step 1: Initial Run

Having set these parameters, we can now run 20 replicate simulations. In doing so, we call the R profiler to keep track of which operations are taking the most time.

```
 # number of replicate model runs. 100 were used in the manuscript
reps=20
d.reps=lapply(1:reps, function(i) 1) # to store output
```

```
Rprof(tmp <- tempfile())
  for(i in 1:reps){
    d=cmstarling3dd(birddisp,landscape,lat0,long0,generations,rate,plantprefs,
      carrying.cap,local.growth,long.distance,birds,num.long.dist,
      recordtimes=recordtimes)
    d.reps[[i]]=d$timeseries
  }
Rprof()
(prof=summaryRprof(tmp)$self)
```

```
##                          self.time self.pct total.time
## "[<-.data.frame"            114.68    41.54     129.76
## "cmstarling3dd"              38.06    13.79     276.08
## "subset"                     21.62     7.83      40.60
## "[.data.frame"               15.92     5.77      23.94
## "subset.default"             14.48     5.24      17.62
## "sample.int"                  8.94     3.24      27.32
## "pmin"                        7.20     2.61      40.40
## "anyDuplicated"               6.62     2.40       8.10
## "match"                       4.32     1.56       6.04
## "tryCatch"                    4.02     1.46      63.04
## "doTryCatch"                  3.62     1.31      56.28
## "vapply"                      2.94     1.06       5.66
## "["                           2.74     0.99      26.68
## "sample"                      2.72     0.99      52.42
## ">"                           2.54     0.92       2.54
## "[<-"                         2.38     0.86     132.14
## "c"                           2.28     0.83       2.28
## "%in%"                        2.16     0.78       8.20
## "length"                      2.06     0.75       2.06
## "names"                       2.06     0.75       2.06
## "FUN"                         1.80     0.65       1.80
## "sys.call"                    1.72     0.62       1.72
## "tryCatchList"                1.50     0.54      58.56
## "anyDuplicated.default"       1.48     0.54       1.48
## ".row_names_info"             0.94     0.34       0.94
```

```
## "match.fun"                    0.92    0.33       0.92
## "NROW"                         0.86    0.31       0.86
## "=="                           0.82    0.30       0.82
## "tryCatchOne"                  0.78    0.28      57.06
## "which"                        0.72    0.26       1.32
## "mostattributes<-"             0.62    0.22       0.62
## "+"                            0.58    0.21       0.58
## "try"                          0.56    0.20      63.60
## "parent.frame"                 0.46    0.17       0.46
## "environment"                  0.44    0.16       0.44
## "pmax"                         0.42    0.15       0.42
## "*"                            0.06    0.02       0.06
## "as.numeric"                   0.02    0.01       0.02
## "pexp"                         0.02    0.01       0.02
##                           total.pct
## "[<-.data.frame"              47.00
## "cmstarling3dd"              100.00
## "subset"                      14.71
## "[.data.frame"                 8.67
## "subset.default"               6.38
## "sample.int"                   9.90
## "pmin"                        14.63
## "anyDuplicated"                2.93
## "match"                        2.19
## "tryCatch"                    22.83
## "doTryCatch"                  20.39
## "vapply"                       2.05
## "["                            9.66
## "sample"                      18.99
## ">"                            0.92
## "[<-"                         47.86
## "c"                            0.83
## "%in%"                         2.97
## "length"                       0.75
## "names"                        0.75
## "FUN"                          0.65
## "sys.call"                     0.62
## "tryCatchList"                21.21
## "anyDuplicated.default"        0.54
## ".row_names_info"              0.34
## "match.fun"                    0.33
```

```
## "NROW"                         0.31
## "=="                           0.30
## "tryCatchOne"                 20.67
## "which"                        0.48
## "mostattributes<-"             0.22
## "+"                            0.21
## "try"                         23.04
## "parent.frame"                 0.17
## "environment"                  0.16
## "pmax"                         0.15
## "*"                            0.02
## "as.numeric"                   0.01
## "pexp"                         0.01
```

It is apparent from the first line of the $by.self element of the R profiling summary that the assignment operator is taking around 47% of the time. This occurs because the state variable for the simulation - the population size in each cell - is frequently updated. The total run time is 276s.

5 Step 2: Change data frames to matrices

To expedite the calculations, we can make a very simple change. The data frame where the population size is stored can be converted to a matrix. We do this below and re-run the same simulations, with profiling.

```
landscape=as.matrix(nelandscape) # the change from the previous version

Rprof(tmp <- tempfile())
  for(i in 1:reps){
    d=cmstarling3dd(birddisp,landscape,lat0,long0,generations,rate,plantprefs,
      carrying.cap,local.growth,long.distance,birds,num.long.dist,
      recordtimes=recordtimes)
    d.reps[[i]]=d$timeseries
  }
Rprof()
(prof=summaryRprof(tmp)$self)
```

```
##                     self.time self.pct total.time
## "subset"                20.94    26.74      37.92
## "subset.default"        16.98    21.68      16.98
## "pmin"                   7.98    10.19      14.56
## "sample.int"             7.42     9.47      24.38
## "sample"                 3.22     4.11      48.94
## "doTryCatch"             3.18     4.06      52.26
## "tryCatch"               3.12     3.98      58.20
## "cmstarling3ddbc"        2.76     3.52      78.32
## "vapply"                 2.58     3.29       5.94
## "FUN"                    2.42     3.09       2.42
## "which"                  1.56     1.99       1.56
## "tryCatchList"           1.36     1.74      54.52
## "match.fun"              0.94     1.20       0.94
## "tryCatchOne"            0.90     1.15      53.16
## "try"                    0.78     1.00      58.98
## "mostattributes<-"       0.62     0.79       0.62
## "parent.frame"           0.56     0.72       0.56
## "environment"            0.54     0.69       0.54
## "pmax"                   0.42     0.54       0.42
## "pexp"                   0.04     0.05       0.04
##                     total.pct
## "subset"                48.42
## "subset.default"        21.68
## "pmin"                  18.59
## "sample.int"            31.13
## "sample"                62.49
## "doTryCatch"            66.73
## "tryCatch"              74.31
## "cmstarling3ddbc"      100.00
## "vapply"                 7.58
## "FUN"                    3.09
## "which"                  1.99
## "tryCatchList"          69.61
## "match.fun"              1.20
## "tryCatchOne"           67.88
## "try"                   75.31
## "mostattributes<-"       0.79
## "parent.frame"           0.72
## "environment"            0.69
## "pmax"                   0.54
```

```
## "pexp"                    0.05
```

Run time has now decreased from 276s to 78.32s.

6 Step 3: Parallelize

The next improvement is to parallelize the replicated simulations. This can be done relatively simply by changing the *for* loop to a *foreach* loop, as shown below. Note that because we use 4 cores, it is most efficient to split the replicated simulations into 4 groups, where each group is computed in sequence on a single core and results are combined only at the end. This avoids delays due to communication between parent and children processes.

```
library(doMC); library(foreach)
registerDoMC(cores=4)

ptm = proc.time()
  #== set up splitting for parallelizing
  ntasks=4 # number of processors to use
  chop.tasks=function(x,n) split(x, cut(seq_along(x), n, labels = FALSE))
  task.split=chop.tasks(1:reps,ntasks)
  #== parallelized loops
  temp=foreach(i = 1:ntasks) %dopar% {
    temp.reps=lapply(1:length(task.split[[i]]), function(i) 1)
    for(k in 1:length(task.split[[i]])){
      d=cmstarling3dd(birddisp,landscape,lat0,long0,generations,rate,
        plantprefs,carrying.cap,local.growth,long.distance,birds,num.long.dist,
        recordtimes=recordtimes)
      temp.reps[[k]]=d$timeseries
    }
    temp.reps
  }
  d.reps=unlist(temp,recursive=F)
(run.time=proc.time()-ptm)['elapsed']
```

```
## elapsed
##    26.2
```

This reduces computation time from 78.3s to 26.2s. The final results are summarized in figure 6.1, here we conducted the full simulation (with 100 replicates) for all of the above code versions.

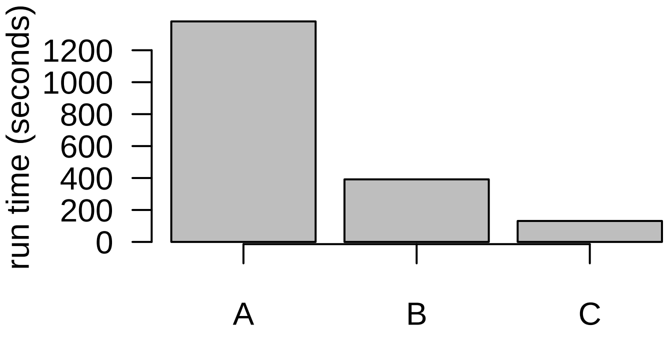

Figure 6.1: Comparison of run times for for 100 replicates of the simulation model (as in the original publication). Labels indicate A) original B) data.frame() converted to matrix(), and C) the parallelized version.
