# Supplementary material for: Speeding Up Ecological and Evolutionary Computations in R; Essentials of High Performance Computing for Biologists
Source: PLoS Comput Biol. 2015 Mar 26;11(3):e1004140. doi: 10.1371/journal.pcbi.1004140 (PMC4374948; doi:10.1371/journal.pcbi.1004140)
Supplement: S3 Text — (PDF) [file pcbi.1004140.s003.pdf]

S3 Text: Optimizing an evolutionary model

Marco D. Visser*[1,2], Sean M. McMahon†[3], Cory Merow‡[3,4], Philip Dixon§[5], Sydne Record¶[6], and Eelke Jongejans‖[1]

[1]Departments of Experimental Plant Ecology and Animal Ecology & Ecophysiology, Radboud University Nijmegen, the Netherlands
[2]Smithsonian Tropical Research Institute, Panama
[3]Smithsonian Environmental Research Center, Edgewater, USA
[4]Department of Ecology and Evolutionary Biology, University of Connecticut, USA
[5]Department of Statistics, Iowa State University, USA
[6]Department of Biology, Bryn Mawr College, USA

January 28, 2015

Contents

1 Model description **2**

2 Summary of the optimization **2**

3 Profiling the original code **3**

4 Parallel execution **12**

5 Re-factoring in C++ **14**

*m.visser@science.ru.nl
†mcmahons@si.edu
‡cory.merow@gmail.com
§pdixon@iastate.edu
¶srecord@fas.harvard.edu
‖e.jongejans@science.ru.nl

The following sections document the steps taken to optimize the code from Visser *et al.* (ref [11] in the main text), shown in Figure 3-D in the main text. The methods used to speed-up this example have been largely covered in the previous appendices (S1 Text, sections 1-6), with the exception of the use of *Rcpp* in the final sections. The interested reader is welcome to follow these steps as a case study.

1 Model description

Mast fruiting, or the intermittent production of large seed crops ("masts") is a reproductive strategy displayed by many plant species worldwide. It is hypothesized that this behavior results in starvation of seed predators between mast years and satiation during mast years which decreases seed predation and enhances regeneration. Visser *et al.* (ref [11] in the main text) built a stochastic population model which attempts to capture this reproductive behaviour for the large stature tropical tree species *Shorea leprosula.* Here, mast fruiting strategies are compared with an annual reproductive strategy by contrasting the costs of delaying reproduction with the benefits of greater seed survival through predator satiation. The model is a stochastic matrix population model where reproduction, growth and survival vary at random. Visser *et al.* (ref [11] in the main text) study the effects of interaction between delaying reproduction and seed predation on the population growth rate. They find that mast-fruiting is strongly favored over annual reproduction when seed predation pressure is strong.

This document will illustrates optimization of the original code, and recreates Figure 3 from Visser *et al.* (ref [11] in the main text). In this figure stochastic simulations were used to calculate the long-term stochastic growth rates (means of 15 replicate simulations) for all combinations of two variables: predation rate (50 levels) and mast frequency (50 levels). The resulting selection landscape predicted the optimal reproductive (mast) frequency for a given predation rate. The re-run of the original code, took 3.5 hours to complete, totalling 37 500 simulations of 10 000 years each.

We only slightly adapt the original code, replacing the stochastic nature of some vital rates with average rates and removing calculations of stochastic elasticities. This was done to decrease complexity and code length, easing comprehension of the code in this appendix. We assume that the reader has worked through Appendix A - our tutorial - and has read the main text before attempting to run these code examples.

2 Summary of the optimization

We optimized the original algorithm by;

1. Conducting profiling exercises which identified un-vectorized use of the function *ifelse()* as a major bottleneck, and we consecutively optimized

the code by replacing *ifelse()* with simple *if* statements. This resulted in an execution time reduction of 1.94 times.

2. Next, standard mathematical operations, such as matrix multiplication, took the most time (e.g. "%*%"). These could not be vectorized due to the stochastic nature of the model and the dependence on the previous state of the population. Therefore we used the Byte Compiler function in R, which resulted in a speed-up of 1.83 times above the previously optimized version.
3. Next, we refactored a critical part of the code in C++ using the *RcppArmadillo* package (which allows - amongst others - the use of C++ linear algebra libraries in R). The refactored section replaced 42 out of 429 lines of code (or 9.8% of the original length). This improved the execution time further by a factor 35.8.

3 Profiling the original code

We start with profiling the original code. We will be using the package *aprof* for this example. In the following sections, we assume that the code below is saved as a file called original.R in the working directory. We will later need this file to profile our code. The code below contains two functions *"onesim"* which completes one simulation of the model over n years and *shormat*, a helper function, which conducts multiple simulations.

```
############################### Shormat #########################
# Simulates population growth by mast fruiting with a stochastic
# matrix population model
##################################################################

############################# CODE START ########################

onesim=function(n=1000,cutp=500,fmat=f.matrix,mat=mat,
mprb=mast.prob,predpen=6,rep.stages=6:7)
{
##################################################################
# Function to run single simulation of n time steps with the
# previously loaded matrices. In the function a fruiting matrix
# (fmat) is stochastically varied with a zero fecundity matrix,
# with prob. mprb, the reproductive elements of fmat are a
# function of the time elapsed since the previous fruiting.
# The long-term average is used to calculate the population
# growth rate (lambda).
# [Note: this is the simplified version no variation in
# growth, survival - and no calculation of stochastic
# elasticities]
```

```
# n = number of time-steps
# cutp = cutoff point for analysis, to discard an x number of
# years to correct for any transient effects
# (following Caswell 2001)
# fmat & mat = fruiting matrix (with average yearly rates, for
# fecundity!) and non fruiting matrix, respectively.
# return.data = logical, returns vector containing years since
# the last fruiting event
# mprb = annual probability of a mast year
# predpen= fraction of seeds lost to predation
################################################################

########################## INITIALIZATION ######################

# Fruiting vector, fruiting vector indicates mast years

fvector <- c(rbinom(n,1,prob=mprb))

# Numerically simulated population growth is saved in N
# Initial population
N1 <- matrix(seq(1000,1,-(1000/dim(fmat)[1])))

# initial phase
# saving average yearly fruit production matrix
amat <- fmat


######## START NUMERICAL SIMULATION OF POPULATION GROWTH ########
i <- 1

# time elapsed since previous fruiting, defines accumulated
# reserves.
te <- 1

# first time step using initial population vector
New.N <- N1

# loop for steps 1:cutpoint (cut point is used to correct for
# transient effects)

for(i in 1:cutp){

        # set fecundity after predation
        fmat[1,rep.stages] <- (amat[1,rep.stages]*te)-
        (predpen*amat[1,rep.stages])
```

```
        #make sure fecundity is not lower than 0
        fmat[1,rep.stages] <- ifelse(fmat[1,rep.stages]<0,
        0,fmat[1,rep.stages])

        # Conduct matrix multiplication
        if(fvector[i]==0) {New.N <- mat%*%New.N;te=te+1}
        else {New.N <- fmat%*%New.N;te=1}

}

N.cutpoint <- New.N

# loop for steps cutpoint to n (n years)

for(i in (cutp+1):n){

        # set fecundity after predation
        fmat[1,rep.stages] <- (amat[1,rep.stages]*te)-
        (predpen*amat[1,rep.stages])

        #make sure fecundity is not lower than 0
        fmat[1,rep.stages] <- ifelse(fmat[1,rep.stages]<0,
        0,fmat[1,rep.stages])

        if(fvector[i]==0) {New.N <- mat%*%New.N;te=te+1}
        else {New.N <- fmat%*%New.N;te=1}
}

N.nyears <- New.N

######## END NUMERICAL SIMULATION OF POPULATION GROWTH #########

# stochastic lambda (long run growth rate)

L <- exp((1/(n-cutp))*(log(sum(N.nyears))-log(sum(N.cutpoint))))

return(L)
}


shormat=function(m=10,n=1500,cutp=500,fmat=f.matrix,
mast.prob=0.15,predpen=0,rep.stages=6:7){
################################################################
# Calculates population growth with onesim()
# for a series of m simulations for n number of time steps
```

```
# m = number of simulations
# n = number of time steps
# cutp = cutoff point for analysis, to discard an x amount
#  of years to correct for any transient effects
# (following Caswell 2001)
# fmat & mat = fruiting matrix (with yearly averages) and
# non fruiting matrix resp.
# mast.prob = annual probability of a mast year
# (equiv. of exp. mast frequency)
# predpen= fraction of seeds lost to predation
##############################################################

Results <- rep(NA,m)

#set zero fecundity matrix
mat<-fmat
mat[1,rep.stages]=0

        for(i in 1:m){
        Results[i] <- onesim(fmat=fmat,mat=mat,
        mprb=mast.prob,n=n,cutp=cutp,
        rep.stages=rep.stages,predpen=predpen)
        }

return(Results)
}
```

Next, let's profile the program, and see where it is spending the most of its time. However, first we need a matrix defining the transitions of individuals over the life cycle of *Shorea leprosula* (the study species).

```
# define matrix for simplified simulations
f.matrix <- structure(c(0.650655890403144, 0.014640764537846,
0, 0, 0, 0, 0, 0, 0.815783111999429, 0.0583850537923953, 0, 0,
0, 0, 0, 0, 0.798164997610948, 0.13163411890922, 0, 0, 0, 0, 0,
 0, 0.92083524123849, 0.0421190583490148, 0, 0, 0, 0, 0, 0,
 0.931474808615322, 0.0479076141721554, 0, 32.0679073140385,
 0, 0, 0, 0, 0.967900035808755, 0.0164662971550416,
 190.014411283517, 0, 0, 0, 0, 0, 0.979252500646358),
  .Dim = c(7L, 7L),  .Dimnames = list(NULL, c("V1", "V2",
   "V3", "V4", "V5", "V6", "V7")))
```

The profiling is done with the next piece of code. In this case it is very important that we switch on line profiling.

```
# Load original.R
source("original.R")

# set seed to reproduce exactly these results
set.seed(1)

# Start the profiling exercise
Rprof(file="original.out",line.profiling=TRUE)
original <- shormat(m=15,n=10000)
Rprof(append=F)
```

Next let's inspect where the program spends most of its time:

```
# load aprof package
require(aprof)

## Loading required package:  aprof

#make aprof object
modelprofile <- aprof("original.R","original.out")
modelprofile

##
## Source file:
## original.R (125 lines).
##
##  Call Density and Execution time per line number:
##
##        Line  Call Density  Time Density (s)
##  [1,]  56    1             0.02
##  [2,]  62    10            0.2
##  [3,]  79    174           3.48
##  [4,]  74    2             0.04
##  [5,]  81    24            0.48
##  [6,]  65    3             0.06
##  [7,]  59    4             0.08
##  [8,]  82    6             0.12
##  [9,]  77    73            1.46
##
##  Totals:
##  Calls  298
##  Time (s)  6.12  (interval =   0.02 (s))
```

We see that there are some clear bottlenecks in a only few lines of code. Then when we summarize the *aprof* object we see that the most promising line for optimization is line 120:

```
summary(modelprofile)

## Largest attainable speed-up factor for the entire program
##
##          when 1 line is sped-up with factor (S):
##
##   Speed up factor (S) of a line
##               1     2     4     8     16    S -> Inf**
## Line*: 79 :  1.00  1.40  1.74  1.99  2.14  2.32
## Line*: 77 :  1.00  1.14  1.22  1.26  1.29  1.31
## Line*: 81 :  1.00  1.04  1.06  1.07  1.08  1.09
## Line*: 62 :  1.00  1.02  1.03  1.03  1.03  1.03
## Line*: 82 :  1.00  1.01  1.01  1.02  1.02  1.02
## Line*: 59 :  1.00  1.01  1.01  1.01  1.01  1.01
## Line*: 65 :  1.00  1.00  1.01  1.01  1.01  1.01
## Line*: 74 :  1.00  1.00  1.00  1.01  1.01  1.01
## Line*: 56 :  1.00  1.00  1.00  1.00  1.00  1.00
##
## Lowest attainable execution time for the entire program when
##
##               lines are sped-up with factor (S):
##
##   Speed up factor (S) of a line
##               1      2      4      8      16
## All lines    6.120  3.060  1.530  0.765  0.383
## Line*: 79 :  6.120  4.380  3.510  3.075  2.858
## Line*: 77 :  6.120  5.390  5.025  4.842  4.751
## Line*: 81 :  6.120  5.880  5.760  5.700  5.670
## Line*: 62 :  6.120  6.020  5.970  5.945  5.933
## Line*: 82 :  6.120  6.060  6.030  6.015  6.007
## Line*: 59 :  6.120  6.080  6.060  6.050  6.045
## Line*: 65 :  6.120  6.090  6.075  6.068  6.064
## Line*: 74 :  6.120  6.100  6.090  6.085  6.083
## Line*: 56 :  6.120  6.110  6.105  6.103  6.101
##
##     Total sampling time:  6.12  seconds
##  *  Expected improvement at current scaling
##  ** Asymtotic max. improvement at current scaling
```

A *targetedSummary* of this line shows us that the function *onesim* is called most often, which in turn calls line 79 the most (*onesim* is the parent call of line 79):

```
head(targetedSummary(target=120,modelprofile,findParent=TRUE))

##   Function Parent Calls Time
```

```
## 1   onesim   L120   297 5.94
## 2      L79 onesim   175 3.50
## 3   ifelse    L79   166 3.32
## 4      L77 onesim    74 1.48
## 5      L81 onesim    24 0.48
## 6      %*%    L81    17 0.34
```

Upon investigation we will see that this line (79) contains the following code:

```
ifelse(fmat[1,rep.stages]<0,0,fmat[1,rep.stages])
```

Which in turn calls the *ifelse* function most often:

```
targetedSummary(target=79,modelprofile,findParent=TRUE)

##    Function Parent Calls Time
## 1    ifelse    L79   158 3.16
## 2       any ifelse    10 0.20
## 3         < ifelse     7 0.14
## 4 is.atomic ifelse     2 0.04
## 5    length ifelse     2 0.04
## 6     is.na ifelse     1 0.02
```

The *ifelse* function is a vectorized function, however, here it was used in a non-vectorized fashion. As *ifelse* contains some "overhead" compared to alternative functions, and its non-vectorized use will incur a large penalty. We can therefore replace the *ifelse* lines with a much simpler statement, which has the same effect.

```
fmat[fmat<0]=0
```

Next we replace lines 79 (and 62 which contains an identical *ifelse* statement) with the above statement. We then save the file as "optimized.R". The reader will have to do this to keep on following these examples. Once this is done, we can see what we have gained and whether the results are identical:

```
# Load optimized.R
source("./optimized.R")

#set seed to reproduce exactly these results
set.seed(1)

# time the execution
system.time(optimized <- shormat(m=15,n=10000))

##    user  system elapsed
##   2.164   0.000   2.169
```

```
# test if the results are really the same
identical(optimized,original)

## [1] TRUE
```

It seems that by simply changing two lines of code we have already achieved a speed-up of about 200%! Next lets see what else can be optimized and whether it is worthwhile to optimize:

```
# Load optimized.R
source("optimized.R")

#set seed to reproduce exactly these results
set.seed(1)

# Start the profiling exercise
Rprof(file="optimized.out",line.profiling=TRUE)
optimized <- shormat(m=15,n=10000)
Rprof(append=F)
```

```
# Look at projected returns
summary(aprof("optimized.R","optimized.out"))

## Largest attainable speed-up factor for the entire program
##
##         when 1 line is sped-up with factor (S):
##
##   Speed up factor (S) of a line
##              1     2     4     8     16    S -> Inf**
## Line*: 77 :  1.00  1.31  1.55  1.71  1.80  1.90
## Line*: 79 :  1.00  1.13  1.20  1.24  1.27  1.29
## Line*: 81 :  1.00  1.09  1.15  1.18  1.19  1.21
## Line*: 62 :  1.00  1.01  1.02  1.02  1.02  1.03
## Line*: 35 :  1.00  1.01  1.01  1.02  1.02  1.02
## Line*: 82 :  1.00  1.01  1.01  1.02  1.02  1.02
## Line*: 65 :  1.00  1.00  1.01  1.01  1.01  1.01
##
## Lowest attainable execution time for the entire program when
##
##              lines are sped-up with factor (S):
##
##   Speed up factor (S) of a line
##              1      2      4      8      16
## All lines    2.320  1.160  0.580  0.290  0.145
## Line*: 77 :  2.320  1.770  1.495  1.358  1.289
```

```
## Line*: 79 :  2.320  2.060  1.930  1.865  1.833
## Line*: 81 :  2.320  2.120  2.020  1.970  1.945
## Line*: 62 :  2.320  2.290  2.275  2.268  2.264
## Line*: 35 :  2.320  2.300  2.290  2.285  2.283
## Line*: 82 :  2.320  2.300  2.290  2.285  2.283
## Line*: 65 :  2.320  2.310  2.305  2.303  2.301
##
##     Total sampling time:  2.32  seconds
##  *  Expected improvement at current scaling
##  ** Asymtotic max. improvement at current scaling
```

We see that no single line jumps out as highly promising. Therefore, lets use the following code to get a general summary of the functions taking the most time.

```
summaryRprof("optimized.out")

## $by.self
##              self.time self.pct total.time total.pct
## "onesim"          1.48    67.89       2.18    100.00
## "<"               0.18     8.26       0.18      8.26
## "*"               0.16     7.34       0.16      7.34
## "%*%"             0.16     7.34       0.16      7.34
## "-"               0.08     3.67       0.08      3.67
## "("               0.04     1.83       0.04      1.83
## "+"               0.04     1.83       0.04      1.83
## ".External"       0.04     1.83       0.04      1.83
##
## $by.total
##              total.time total.pct self.time self.pct
## "onesim"           2.18    100.00      1.48    67.89
## "shormat"          2.18    100.00      0.00     0.00
## "<"                0.18      8.26      0.18     8.26
## "*"                0.16      7.34      0.16     7.34
## "%*%"              0.16      7.34      0.16     7.34
## "-"                0.08      3.67      0.08     3.67
## "("                0.04      1.83      0.04     1.83
## "+"                0.04      1.83      0.04     1.83
## ".External"        0.04      1.83      0.04     1.83
## "rbinom"           0.04      1.83      0.00     0.00
##
## $sample.interval
## [1] 0.02
##
## $sampling.time
## [1] 2.18
```

The output of *summaryRprof()* shows us that most time is spent within *"onesim()"* executing basic mathematical operations. In cases like these the only options for increased speed would be to conduct the computations in parallel or to re-factor the key parts of the code (i.e. rewrite most lines returned by *aprof()* above in a lower-level language like C or Fortran). We give an example on how to do both in the next sections. However, we should try to use R's Byte compiler first (see the main document or online text S1 for details). This is a highly simple procedure, as shown in the next section of code, where we byte compile the function *"onesim()"*, time its execution and test whether the results are identical to our first code example.

```
# Byte compile onesim
onesim <- compiler::cmpfun(onesim)

# Time the code execution
set.seed(1)
system.time(bytecompiled<- shormat(m=15,n=10000))

##    user  system elapsed
##   1.536   0.000   1.539

# test if the results are identical
identical(bytecompiled,original)

## [1] TRUE
```

We see that the Byte Compiler improved the code even further from roughly 3 seconds to 1.5.

4 Parallel execution

We can further speed-up calculations by replacing the *shormat()* function with a parallel version. Details on parallel computing can be found in the main text and in online text S1. The parallel code we used to replace the original *shormat()* was (note that we are using parallel execution through forking which is not compatible with windows - see online text S1 for a windows example):

```
shormat <- function(m=10,n=1500,cutp=500,fmat=f.matrix,
mast.prob=0.15,predpen=0,rep.stages=6:7,ncores=2){

# start dividing problem for parallel computing
Workload <- table(cut(1:m,ncores,labels=F))

# Ensuring independence of Random number sequences
# set RNG to "L'Ecuyer-CMRG"
RNGkind("L'Ecuyer-CMRG")
```

```
# Calling the following will make
# runs from mcparallel reproducible
mc.reset.stream()

# make object to store child id's
childs<-vector("list", ncores)

        for(i in 1:ncores){

        # start parallel streams
        childs[[i]]<-mcparallel(runonesim(
                                        m=Workload[i],fmat=fmat,
                                mat=mat,mprb=mast.prob,
                                n=n,cutp=cutp,
                                rep.stages=rep.stages,
                                predpen=predpen))
                }

# collect results
final<-mccollect(childs)

# Reset RNG to default
RNGkind("Mersenne-Twister")

# reshape final and make useful
return(unlist(final))
}


# The following function will be run in parallel
# within shormat

runonesim <- function(m,fmat,mat,mprb,n,cutp,
        rep.stages,predpen){

Results=rep(NA,m)

mat<-fmat
mat[1,rep.stages]=0

        for(i in 1:m){
        Results[i]=onesim(fmat=fmat,mat=mat,mprb=mprb,n=n,
        cutp=cutp,rep.stages=rep.stages,predpen=predpen)

        }
```

```
return(Results)
}
```

Now that we have replaced *shormat()* with a parallel version, let see how much speed we have gained:

```
# load parallel package
require(parallel)

## Loading required package:  parallel

# Time the code execution with 4 cores
system.time(parallel <- shormat(m = 15, n = 10000, ncores = 4))

##    user  system elapsed
##   0.414   0.414   0.419
```

With the parallel execution we see that we have cut execution time down from 1.5 to 0.414 seconds. Note that we did not test whether our results were identical. This is because it is good practice to use a special random number generator (RNG) when conducting parallel calculations (see online text S1 and the main document for details). As the RNG is different here we should not be surprised that the results are not identical. Closer evaluation, however, reveals that the mean values of the simulations are indistinguishable.

5 Re-factoring in C++

In the next sections we use the *RcppArmadillo* package (Eddelbuettel & Sanderson, 2013) and replace the core simulation code in the function *onesim()* with a function written in C. Readers who would like to learn more about the below code are advised to read the *Rcpp* and *RcppArmadillo* documentation - which are highly accessible (Eddelbuettel & François, 2011; Eddelbuettel & Sanderson, 2013).

In the below example we reload our optimized serial R version, this is so we can later confirm that our C++ function returns identical results to our first program (we can't do this with the parallel version as we use a different random number generator in that example). It should be clear that before the next code can be run, a working installation of *RcppArmadillo* and all its dependencies are needed.

```
# load optimized
source("optimized.R")

# create Rccp function
# define function
require(Rcpp)
```

```
## Loading required package:  Rcpp

require(RcppArmadillo)

## Loading required package:  RcppArmadillo

require(inline)

## Loading required package:  inline
## Loading required package:  methods
##
## Attaching package:  'inline'
##
## The following object is masked from 'package:Rcpp':
##
##    registerPlugin

 src <- '
  arma::mat A = Rcpp::as<arma::mat>(amat);
  arma::mat F = Rcpp::as<arma::mat>(fmat);
  arma::mat N = Rcpp::as<arma::mat>(NNew);
  Rcpp::NumericVector FV = Rcpp::NumericVector(fvec);
  Rcpp::NumericVector PR = Rcpp::NumericVector(pred);
  int n = FV.size();

  double te = 1,fr1 = F(0,5), fr2 = F(0,6);  // Return to R with N

   for (int i=1; i<n; i++) {

    F(0,5) = (fr1 * te)-(fr1*PR[0]);
    F(0,6) = (fr2 * te)-(fr2*PR[0]);
    if(F(0,5)<0) { F(0,5) = 0; }
    if(F(0,6)<0) { F(0,6) = 0; }

    if(FV[i]==1){
    N = F*N;
    te = 1;
    }

   else {
    N = A*N;
    te++;
   }

  }
  return Rcpp::wrap(N);  // Return to R with N
'
```

```
## create the compiled function
rcppNsim <- cxxfunction(signature(amat="numeric",fmat="numeric",
                                  NNew="numeric",fvec="numeric",
                                  pred="numeric"),
                        src,plugin="RcppArmadillo")
```

Next we replace our old version of *onesim* with one where the key calculations are conducted by the above function.

```
onesim=function(n=1000,cutp=500,fmat=f.matrix,mat=mat,
mprb=mast.prob,predpen=6,rep.stages=6:7)
{
#################### INITIALIZATION ######################

# Fruiting vector, fruiting vector indicates mast years

fvector <- c(rbinom(n,1,prob=mprb))

# Numerically simulated population growth is saved in N
# Initial population
N1 <- matrix(seq(1000,1,-(1000/dim(fmat)[1])))

# initial phase
# saving average yearly fruit production matrix
amat <- fmat

#make zero fruiting matrix for use in rcppNsim
zmat <- fmat
zmat[rep.stages] <- 0

###### START NUMERICAL SIMULATION OF POPULATION GROWTH #####

# first time step using initial population vector
New.N <- N1

# Use Rcpp function to replace loop for steps 1
# to cutpoint
New.N <- rcppNsim(zmat,fmat,N1,
                              fvector[1:cutp],predpen)

N.cutpoint <- New.N

# loop for steps cutpoint to n (n years)
```

```
# Use Rcpp function to replace loop for steps
# cutpoint to n (n years)
New.N <- rcppNsim(zmat,fmat,N.cutpoint
                        ,fvector[(cutp+1):n],predpen)

N.nyears <- New.N

###### END NUMERICAL SIMULATION OF POPULATION GROWTH #########

# stochastic lambda (long run growth rate)

L <- exp((1/(n-cutp))*(log(sum(N.nyears))-log(sum(N.cutpoint))))

return(L)
}
```

Finally, let's time our code to see if all our efforts have paid-off.

```
system.time(Rcppversion<-shormat(m=15,n=10000))

##    user  system elapsed
##   0.024   0.000   0.024
```

We see that it has most certainly paid-off: we started with an execution time of 5.7 seconds and end-up with about 0.03, that is a speed-up of 190 times! Using the new code we recreated Figure 3 from Visser *et al.* 2011 (Figure 5.1). In the original document the amount of time required to create the figure in panel **A** was 217.7 minutes, using the new code we were able to increase the resolution and number of simulations greatly from a total of 37 500 to 13 500 000 before the run time was similar to the original (214.4 minutes). This amounts to an increase in speed where we are now able to conduct 63054.7 simulations per minute (panel b) compared to only 172.3 simulations per minute using the original code. In total no more than 10% of the code was altered.

References

Eddelbuettel, D. & François, R. (2011) Rcpp: Seamless r and c++ integration. *Journal of Statistical Software*, **40**, 1–18.

Eddelbuettel, D. & Sanderson, C. (2013) Rcpparmadillo: Accelerating r with high-performance c++ linear algebra. *ComputationalStatistics and Data Analysis*, **in press**.

Visser, M.D., Jongejans, E., van Breugel, M., Zuidema, P.A., Chen, Y.Y., Kassim, A.R. & de Kroon, H. (2011) Strict mast fruiting for a tropical dipterocarp tree: a demographic cost-benefit analysis of delayed reproduction and seed predation. *Journal of Ecology*, **99**, 1033–1044.

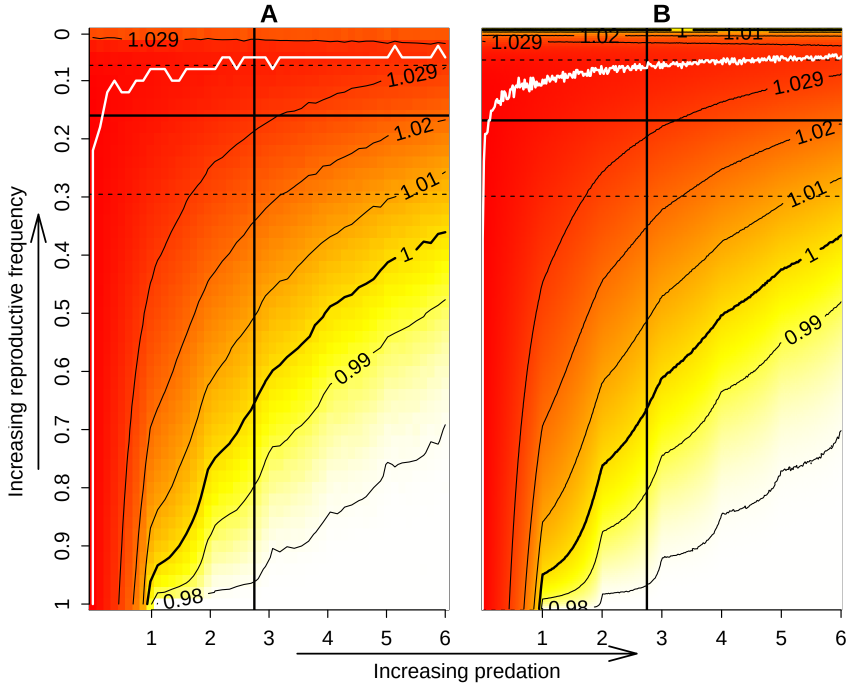

Figure 5.1: Re-calculation of the model in Visser *et al* (2011), where the optimal reproductive frequency (y-axis) is calculated at various seed predations rates (x-axis). The graphs show contour plots depicting the joint relationship of seed predation (x) and delayed reproduction (y) on the population fitness. Within each graph the white line shows the 'crest' of the fitness landscape or the maximum value for each combination of x and y (hereafter "pixel"). The vertical and horizontal solid lines are the estimates of the observed reproductive frequency and seed predation (see Visser *et al.* 2011 for details). Panel **A** is the the result rerunning the original code for 2500 (50 x 50) iterations in x and y, with each pixel value being the mean of 15 simulations (totalling 37 500 simulations). Creation of panel **A** took 217.7 minutes. In panel **B** exactly the same simulations are run with an optimized version of the code. Here we increased the detail by a factor of 360. This was done in such a way that the total execution time, here 214.1 minutes, closely matches that of panel **A**. Panel **B** has 90 000 pixels (300 x 300) compared to 2500 in panel **A**, while the total number of completed simulations was 13 500 000 (each pixel is the mean of 150 simulations) compared to 37 500 simulations in panel **A**.
